# The effectiveness of various computer-based interventions for patients with chronic pain or functional somatic syndromes: A systematic review and meta-analysis

**DOI:** 10.1371/journal.pone.0196467

**Published:** 2018-05-16

**Authors:** Miel A. P. Vugts, Margot C. W. Joosen, Jessica E. van der Geer, Aglaia M. E. E. Zedlitz, Hubertus J. M. Vrijhoef

**Affiliations:** 1 Tranzo Scientific Center for Care and Welfare, Tilburg School of Social and Behavioral Sciences, Tilburg University, Tilburg, the Netherlands; 2 Department of Health Medical and Neuropsychology, Leiden University, Leiden, the Netherlands; 3 Department of Patient & Care, Maastricht University Medical Center, Maastricht, the Netherlands; 4 Department of Family Medicine and Chronic Care, Vrije Universiteit Brussel, Brussels, Belgium; 5 Panaxea B.V., Amsterdam, the Netherlands; Southeast University Zhongda Hospital, CHINA

## Abstract

Computer-based interventions target improvement of physical and emotional functioning in patients with chronic pain and functional somatic syndromes. However, it is unclear to what extent which interventions work and for whom. This systematic review and meta-analysis (registered at PROSPERO, 2016: CRD42016050839) assesses efficacy relative to passive and active control conditions, and explores patient and intervention factors. Controlled studies were identified from MEDLINE, EMBASE, PsychInfo, Web of Science, and Cochrane Library. Pooled standardized mean differences by comparison type, and somatic symptom, health-related quality of life, functional interference, catastrophizing, and depression outcomes were calculated at post-treatment and at 6 or more months follow-up. Risk of bias was assessed. Sub-group analyses were performed by patient and intervention characteristics when heterogeneous outcomes were observed. Maximally, 30 out of 46 eligible studies and 3,387 participants were included per meta-analysis. Mostly, internet-based cognitive behavioral therapies were identified. Significantly higher patient reported outcomes were found in comparisons with passive control groups (standardized mean differences ranged between -.41 and -.18), but not in comparisons with active control groups (*SMD* = -.26 - -.14). For some outcomes, significant heterogeneity related to patient and intervention characteristics. To conclude, there is a minority of good quality evidence for small positive average effects of computer-based (cognitive) behavior change interventions, similar to traditional modes. These effects may be sustainable. Indications were found as of which interventions work better or more consistently across outcomes for which patients. Future process analyses are recommended in the aim of better understanding individual chances of clinically relevant outcomes.

## Introduction

Computer-based interventions (CBIs) may be a particularly accessible means for improving health outcomes in patients with chronic pain (CP) or functional somatic syndromes (FSS) [1, 2]. CP is diagnosed in individuals seeking health care for pain symptoms that persist beyond a usual 3- to 6-month duration of organic recovery [3]. FSS are defined by functional disturbances and chronic somatic symptoms without a satisfactory explanation by organ pathology or disease [4]. The difference between CP and FSS is the “mandatory” presence of disturbing pain symptoms in CP that can accompany a disease (i.e., arthritis) and does not need to be present for the diagnosis of FSS (i.e., chronic fatigue syndrome, tinnitus). However, there is a vast overlap between CP and FSS. Both conditions cover a variety of bodily symptoms and involve organic systems, and several diagnoses fall under both definitions (i.e., fibromyalgia, chronic low back pain, and irritable bowel syndrome [IBS]) [4, 5]. Furthermore, evidence supports bio-psychosocial management strategies based on a stepped-care approach for both CP and FSS [4, 6]. Efficacious interventions for reducing experienced symptoms and functional interference, in order of increasing intensity, include conservative medical treatment, physical therapy, psychotherapy, or multidisciplinary rehabilitation in primary or specialized care settings [4, 6, 7]. Herein, CBIs could offer independent patient access to stand-alone programs or complementary elements for further-reaching, stronger, or more enduring effects by increasing independent engagement and/or preventing relapse [8, 9].

Research and development of CBIs is motivated by the large burden of these disorders that are due to a high prevalence (e.g., 20–30% for CP [10, 11], 1–3% for chronic fatigue syndrome [12], and 10–15% for tinnitus [13]), co-morbid psychological distress, loss of productivity, absence of strongly effective medical treatment, and limited access to specialized health care [3, 4, 11]. CBIs may involve the use of the Internet [14], interactive voice response [9], mobile/smart phone applications [15], CD-ROM/DVD, or handheld computers as a delivery route [16].

### Literature overview

Previous systematic reviews and meta-analyses investigated the impact of CBIs across populations with chronic disease or mental health problems, including CP and FSS conditions [2, 14, 17–33]. Internet-delivered cognitive behavioral therapy (CBT) is effective compared to waiting-list or usual care conditions and may be equivalent to traditional (face-to-face) delivery formats [14, 18, 34]. This was suggested for CP patients specifically in a systematic review and meta-analysis by Buhrman et al. [23] that included 22 randomized controlled trials (RCTs) (five were published by the first author). Applied Internet-based CBT was described in narratives and modest effect size estimates were found for patients’ reported pain intensity (Hedges’ g = -.33), functional interference or disability (g = -.39), catastrophizing (g = -.49), and depression (g = -.26), which replicated earlier meta-analytic findings [14]. A study on the impact of self-help modalities (including CBIs) in patients with IBS found medium sized effects on somatic symptoms (standardized mean difference [*SMD*] = -.72), and a large effect on quality of life (*SMD* = -.84) that did not differ between computer-based or face-to-face formats [27]. Conclusions on the internal and external validity of these findings were drawn with caution due to a limited amount of high-quality randomized clinical trials (RCTs). It was suggested that future studies should focus more on methodological uniformity and quality, outcomes at long-term follow-up, direct one-to-one comparisons with various active treatments, and/or extended variety in participants and treatment settings [18, 24, 26].

Furthermore, there are knowledge gaps with regard to what works for whom, and when [34–40]. It remains unclear if CBI effects vary by intervention, patient (e.g., demographics), and context factors (e.g., the degree to which e-health trials resemble routine applications) [14, 15, 41, 42]. To meet the presumed potential of CBIs, developers and (clinical and policy) decision makers yet require knowledge about which CBIs will be effective for which patients with CP or FSS in actual health care settings [28, 43, 44]. Process analyses embedded in clinical trials can offer the best evidence on these matters and can be complemented with meta-analytic tests [40, 45]. Statistically significant moderators of outcome improvement were found in CP patients after Internet-delivered CBT in comparison with controls, but there was no consistent moderating factor across outcome domains [39]. Several studies suggested similar degrees of CBI effectiveness across sub-populations, but participant (self) selection could have restricted the observed amount of patient variation [35, 36, 38, 39]. Two meta-analyses, comprising a diversity of self-management interventions and patients with musculoskeletal pain, explored moderators of program effectiveness [34, 37]. One found that professional guidance and psychological components were associated with better outcomes [34]. The other one showed stronger effects in older participants but guarded against definite conclusions based on a limited amount of data [37].

### Objectives

In order to aid decision makers in choosing the appropriate intervention strategy for specific populations and individuals, and to aid CBI developers in constructing effective interventions, the objectives of this meta-analysis were to establish the efficacy of CBIs and to elucidate patient and intervention characteristics. In light of ongoing accumulation of empirical data and the possibility of pooling the results from CBI effect studies for the largely homogeneous conditions of CP and FSS, the questions of this study where thus: (1) To what extent do CBIs result in better health outcomes after treatment and at follow-up experienced by patients with CP or FSS as compared to passive control conditions (i.e. waiting-list, usual or standard care, discussion boards, or standard patient information) and active treatment conditions?; (2) What are the characteristics of patients for whom computer-based interventions are most and least effective?; and (3) What are the characteristics of the most and least effective computer-based interventions? Based on existing evidence about CBIs, general positive effects, but no specific moderating patient or intervention factors were expected.

These objectives include consideration of the strength of evidence that depends on methodological threats to internal and external validity [46]. Important health outcomes for CP and FSS are patient-reported somatic symptom intensity, health related quality of life (HRQOL), functional interference (or disability, handicap, impact, or disturbance of activities due to somatic symptoms), catastrophizing (or acceptance, self-efficacy, or any other targeted cognitive process of outcome improvement) [47], and depression (as a commonly reported aspect of emotional distress) [33].

## Methods

The Cochrane Handbook of systematic reviews of interventions [45] was used to prepare the study protocol which was preregistered at PROSPERO (2016:CRD42016050839). Reporting was then guided by the PRISMA statement [48].

### Inclusion and exclusion criteria

Study inclusion and exclusion were based on: patient (P), intervention (I), comparison (C), outcome (O), and study type (S) criteria (PICOS) [48]. Eligible studies focused on adult participants (17–67 years of age) with a CP or a FSS condition. CP or FSS was identified through case-finding (with valid diagnostic instruments) or clinician assessment (either during the study or before, as a basis for self-reference). This included somatic symptom disorders and medically unexplained physical symptoms (MUPS). Eligible studies investigated a computer-based intervention; in comparison with one or more control conditions of any kind (passive or active); for its effects on relevant health outcomes; in a RCT, quasi-experiment, or mixed-method study. Measurements were taken at baseline and post-intervention and/or at follow-up. CBIs are defined as programs that require patient contributions by using a computer platform for direct access to personally relevant information and support in behavioral change and/or decision-making for health issues [49].

Studies were excluded: (1) if patient eligibility focused specifically on pediatric or geriatric populations, factitious disorders, a specific organic disease (e.g., migraine, multiple sclerosis, osteoarthritis) or psychiatric illness as a complication thereof, hypochondriasis [50], or individuals who did not report chronic somatic symptoms (e.g., individuals at risk targeted in primary prevention); (2) if experimental programs were not CBIs (e.g., if a program did not target patients themselves, was designed to be used exclusively with professional assistance, regarded participants as passive recipients, or only provided a means for distant communication with care providers); (3) if outcomes other than relevant health outcomes were prioritized (i.e., feasibility or technology acceptance), or narrow focus on somatic symptom outcomes (the only type of outcome reported); and (4) if study types were non-empirical, fully qualitative, uncontrolled, or not published as a full-bodied article in a peer-reviewed scientific journal.

### Search strategy

On June 16th and July 1st, 2016, MEDLINE, EMBASE, PsychINFO, Cochrane Central Register of Controlled Trials (CENTRAL), and Web of Science were searched for relevant studies published since January 1990 without language constraints. Search terms relating to the patient populations [51, 52], computer-based and behavioral interventions [49, 53], and study types [49] from previously published Cochrane reviews were listed using the Boolean operator ‘or’ and combined using the Boolean operator ‘and’. The search string was adapted for usage across bibliographic databases with available interfaces. The full search strategy for EMBASE is added in Table A in S1 Appendix. As additional methods to obtain an exhaustive set of peer-reviewed and published journal articles, references of previous systematic reviews and meta-analyses on related topics were checked [2, 14, 17–20, 22–29, 31, 32, 34, 54–57], and backward and forward citations of eligible studies were checked in Web of Science [58]. Grey literature was not searched.

### Study selection

A two-step selection protocol had been piloted, used, and refined. First, potentially eligible studies were identified by titles and/or abstract screenings. Two authors (MV and HV or MJ) independently screened half of the studies. After comparison, discussions revealed that none of the authors had excluded potentially eligible articles. Thus, MV screened the remaining half. Second, MV and MJ independently assessed the final full-text assessment of all potentially eligible titles, and discrepancies were resolved by discussions involving HV. Each study with relevant outcome data was eligible for meta-analysis.

### Data extraction and management

A data-extraction form was composed, piloted, and discussed *a priori*. General and patient items were extracted by MV and checked by AZ. Relevant items for generalizability to routine applications according to the CONSORT statement for E-health [46] were among these general items: year of publication, setting (by continent), type of control group, methods of recruitment (“open” or “closed” population, participant screening methods), participant compensation, type of human involvement, and use of prompts/reminders. Patient items were (baseline) average age, proportion of females, the duration of symptoms, education level (proportion that completed tertiary education), employment, sick leave, depression, and somatic symptom intensity.

Intervention duration, compliance, and characteristics were independently extracted by MV and JG. Disagreements were resolved after discussion with AZ. Theoretical basis, mode of delivery and behavior change techniques (BCTs) were classified based on intervention descriptions using the uniform taxonomies from Webb et al. [59]. Accordingly, the 11 items on use of theory were clustered into three categories: referencing to underpinning theory, targeting of relevant theoretical constructs, and selecting recipients or tailoring interventions. The 11 items on mode of delivery were clustered into automated functions, communicative functions, and supplementary modes. For classifying BCTs, we used the updated Behavior Change Technique Taxonomy version 1 (BCTTv1) [25, 60]. This is a hierarchically structured taxonomy of 93 distinct techniques that are grouped into 16 categories such as “goals and planning” and “social support”. Both coders were trained in the accurate application of the BCTTv1.

Two authors (MV and JG) extracted and double-checked all outcome information, including the administered self-assessment instruments, means, standard deviations, and sample-sizes for two “time points”: post-treatment and/or 6 months or more at follow-up. If multiple measures were available for the same outcome category, the following measures were preferred: visual analogue or numerical rating scales of pain intensity (current) for somatic symptom intensity, HRQOL total scores (general subjective health or mental health composite subscales if totals were not reported), (pain) interference for functional interference (otherwise disability, handicap, or disease impact), and catastrophizing (or acceptance/self-efficacy). Standard errors were converted into standard deviations. Baseline values were imputed for missing standard deviations for outcomes post-treatment or at follow-up (i.e., if only change scores were reported).

### Risk of bias rating

Quality assessment of the studies was performed by MV and JG based on the 13 risks of bias criteria recommended by the Cochrane Collaboration Back Review group [61]. Discussions with HV enhanced the objectivity and consistency of this assessment. The columns of Table B in S1 Appendix detail the 13 criteria. Subsequently, the 13 criteria were combined into seven major categories of the general Cochrane risk of bias tool: selection bias, attrition bias, reporting bias, performance bias, incomplete data extraction bias, detection bias, and other risks [62]. Methodological limitations that pose a general threat for this type of intervention studies (lack of blinding) were ruled out in this categorization. A category was scored “high risk” if high risk was scored for one or more underlying criteria, assuming that a single source of risk could bias the results of a trial completely [63]. “Low risk” was scored if all underlying criteria were “low risk”.

### Determining the efficacy of computer-based interventions

To estimate CBI outcome levels against controls, pooled effect sizes were calculated by using the Review Manager Software package (RevMan 5.3) [45, 64]. Comparisons were categorized as CBI versus passive controls (e.g., “waiting-list”, “usual/standard care”); or CBI versus active controls (e.g., same content in face-to-face format). Outcome data were inserted such that negative numbers represented lessening of symptoms and overall that lower numbers represent more favorable outcomes for intervention group participants. If a comparison had to be chosen from multiple relevant options within a study (with multiple CBIs), the newest and/or most elaborate CBIs (i.e., “third-wave” CBT, or with more BCTs and/or delivery modes) were designated as experimental, while the simplest and most traditional interventions were chosen as controls. Twenty primary meta-analyses were performed for the two comparison types and five outcome categories by the two time-points.

For each meta-analysis, RevMan operations were set for inverse variance methods of estimating random effects on the basis of standardized differences between intervention and control group means (*SMD*s), anticipating on heterogeneous estimates in continuous outcomes and the use of different questionnaire instruments across studies. Chi-squared tests indicated if there was significant heterogeneity of *SMD*s across studies (cut-point: *p* < .05), and the I^2^ statistic indicated the extent to which heterogeneity affected the pooled result. Applicable thresholds for (rough) interpretations of I^2^, with 0% to 40% as potentially unimportant, 30% to 60% as modest, 50% to 90% as substantial and 75% to 100% as considerable heterogeneity were conservatively applied [45]. Funnel-plots were visually inspected for indications of publication bias. Further analyses, by calculating *SMDs*, on risk of bias sensitivity were performed only for studies that were assessed low risk of bias for each category. Hereto, it was also checked if study level sources of risk were similar on the outcome level (i.e., if unbalanced baseline group scores were a risk for a particular outcome). Similar sensitivity analyses were conducted based on source of recruitment (“open” versus “closed”) to explore effects by differences in health care settings [46].

### Determining patient and intervention characteristics of effective computer-based interventions

Per meta-analysis with statistically significant and “potentially important” heterogeneity, two sub-sets of studies were created: one for the 25% highest study group differences (*SMD*s) and one for the 25% lowest *SMD*s. Within each set, patient and intervention characteristics (potential effect modifiers) were described by a summery statistic (count, proportion, or mean). To reduce the number of plausible sub-group analyses, characteristics were deemed ‘distinctive’ and tested (χ^2^) if they differed substantially between the two sets, and/or from expected values (within sets of all studies or comparison types). Analyses were only conducted if 10 or more studies were available for analysis [45]. Study level associations (Chi-square tests, Kendall’s Tau, or Pearson correlations) were calculated between intervention and patient factors to examine potential confounding of modifiers (using SPSS 22).

## Results

### Search

**Fig 1:**
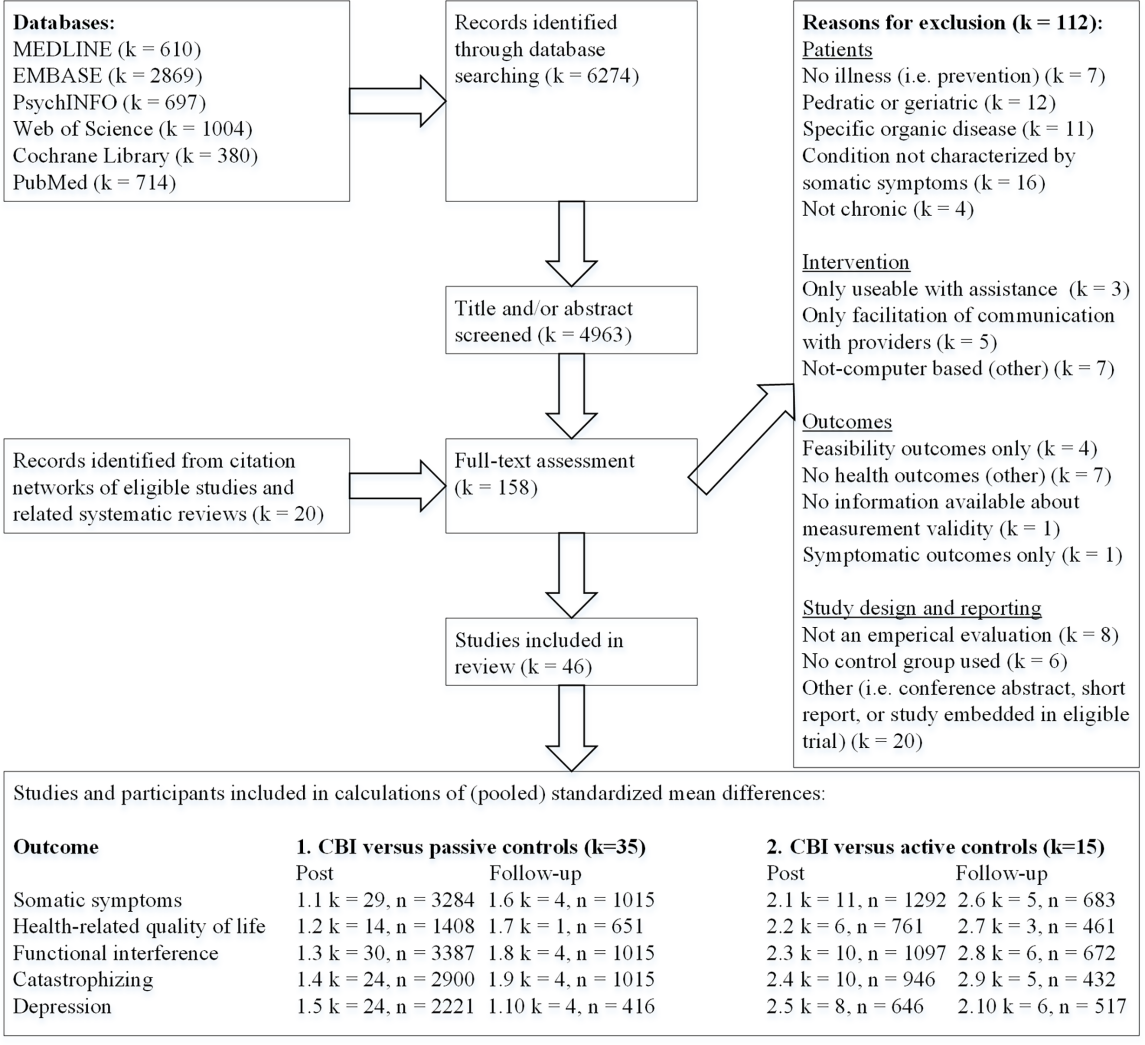
PRISMA flow-diagram of studies. Abbreviations and symbols: k = number of studies, *n* = number of study participants, OC = outcome, SS = Somatic Symptoms, HRQOL = Health Related Quality Of Life, FI = Functional Interference, CAT = Catastrophizing, DEP = Depression.

Search and study selection procedures are summarized in the PRISMA flow-diagram (Fig 1). In total, 4,963 unique hits were identified from the databases. Twenty additional studies were found in the citation networks of eligible studies or references of systematic reviews or meta-analyses on related topics. After tentative steps of title and abstract screening, 158 studies remained, nine of which were short reports or conference abstracts. Therefore, 149 full-bodied peer-reviewed articles were assessed full-text on the alleged inclusion and exclusion criteria. The final set consisted of 46 eligible studies (k) [9, 16, 65–108].

### Study characteristics

Over time an increase in eligible publications was found. Whereas in the period 2000–2007 only zero to one article per year had been published, this number rose to six to seven per year in the years 2013–2015. Of the included studies, only one study was not an RCT. Thirty-seven studies compared a CBI with a passive control condition (waiting-list, k = 14, usual or standard care, k = 9, message/discussion board, k = 8, provision of information, k = 6), whilst 16 studies compared a CBI with an active control condition (a simpler version of the same CBI, k = 9, active treatment without the additional CBI, k = 3, or face-to-face CBT group, k = 4). Six studies had multiple arms including comparisons with passive as well as active conditions. Three studies (two passive and one active comparisons) did not contain sufficient information for extracting means and standard deviations. Twenty-nine studies were based in Europe, 13 in the US, three in Australia, and one in Asia. In 28 studies, participants were recruited from a general “open” population (e.g., web-site enrollment) and screened for eligibility using web-forms (k = 13), additional telephone interviews (k = 8), or face-to-face interviews (k = 6). Seventeen studies recruited exclusively from “closed” clinical or work settings. Seven studies recruited from open as well as closed populations, and one did not report recruitment source. Most studies explicitly selected participants with the ability to use the required computer technology, including the Internet (k = 32), touch key telephone (k = 1), or smartphone (k = 1). More implicit selection procedures were present in 12 studies, of which three studies used a run-in period. In six studies, monetary compensation was provided for study participation. About 60% of the included subjects completed the interventions (proportion on average was .59, *SD* = .23, range = .21–1, k = 31). Table C in S1 Appendix contains an overview of the questionnaire instruments for which data were extracted across outcome categories.

### Participants

Patient conditions targeted by CBIs were mixed chronic pain (k = 15), chronic (low) back pain (k = 6), chronic widespread pain/fibromyalgia (k = 6), mixed or tension headache (k = 3), IBS (k = 7), chronic fatigue (k = 1), interstitial cystitis (k = 1), non-cardiac chest pain (k = 1), and tinnitus (k = 6). Participants were on average 45.4 years of age (*SD* = 5.2, k = 44). Average proportions of patient characteristics showed that 71% of the participants were female (*SD* = .22, k = 45), 42% had completed tertiary education (*SD* = .16, k = 25), 67% were employed (*SD* = .19, k = 21), and 36% were on sick leave (*SD* = .27, k = 15). Somatic symptoms prior to treatment were reported for a mean duration of 115 months (*SD* = 31, k = 26), and studies that reported HADS depression at baseline (k = 14) generally found no indication of depressive disorders (mean = 6.7, *SD* = 1.3).

### Intervention characteristics

Experimental CBIs had an average duration of 10.5 weeks (range = 3–52, *SD* = 8.9) and were mostly (k = 30) guided by one or more health professionals (mode = 1, median = 3, range = 1–16; master’s level psychologists, k = 12; clinically trained, k = 14). Most studies made use of prompts or reminders (k = 31) that were sent occasionally depending on compliance (k = 21) and/or scheduled automatically (k = 13). Behaviors mostly targeted by CBIs included exercise, sleep hygiene, relaxation, and leisure activities.

#### Theoretical basis and use of theory

Table D in S1 Appendix presents the complete coding results for use of theory (1). To summarize, CBT approaches prevailed (k = 33) across the studies that mentioned or referred to a theory about relationships among relevant concepts (item 1, k = 39). Sixteen studies explicitly described their approach as CBT. Seven studies specifically mentioned third-wave CBT approaches, including Acceptance and Commitment Therapy (ACT) and mindfulness-based therapies. Others mentioned a combination of CBT with third-wave (k = 6) or other conceptualizations (k = 4). The remaining studies either referred to the empowerment model (k = 3) or a model constructed by the author (k = 3). Targeted constructs from the theory were mentioned as a predictor of behavior (k = 11) and/or for selection of intervention techniques (k = 21). Theory or predictors were rarely used to select recipients for the intervention (k = 1) or tailor the intervention to recipients (k = 1). Explicit descriptions of links between techniques and relevant constructs were identified in 19 studies.

#### Behavioral change techniques

**Fig 2:**
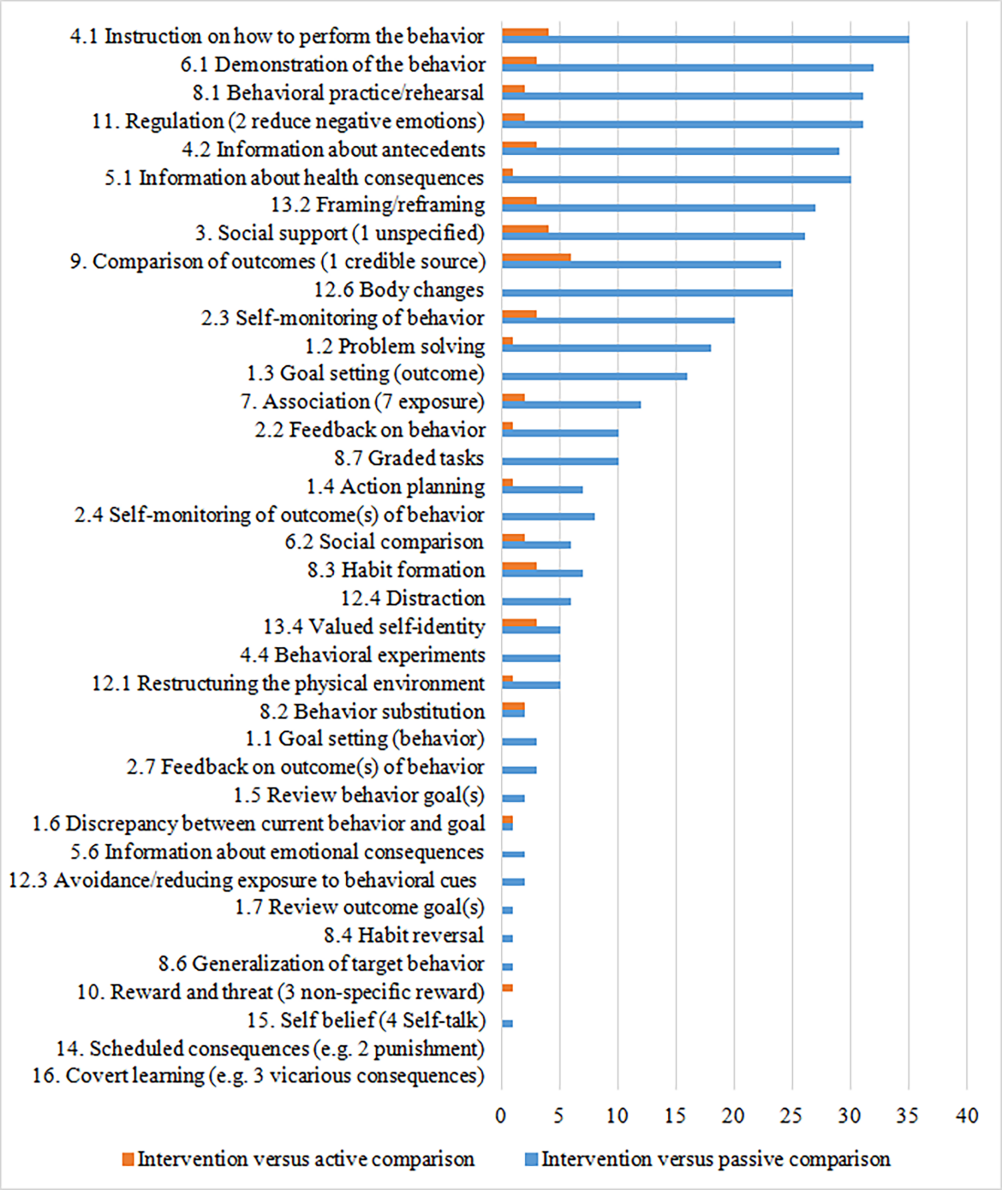
Numbers of studies in which behavioral change techniques were identified by comparison.

Fig 2 summarizes for how many studies certain BCTs were coded by each comparison. Table D in S1 Appendix (2) fully describes the study and comparison level coding results after listing the precise interpretations of the coders of the 93 BCTs across the 16 categories. Techniques implied by the description of ‘relaxation’ or ‘meditation’ were coded most often (k = 31–37). Those techniques included the codes of performance instructions (BCT code 4.1), demonstration (6.1), prompting practice and rehearsal (8.1), and reduction of negative emotions (11.2). Therefore, these were the most prevalently coded BCTs. Body changes (12.6) was coded as implied by the description of relaxation (k = 25), but not of meditation. Intervention descriptions often mentioned that change support was delivered by trained professionals over the internet (k = 27). Herein the BCTs unspecified social support (3.1) and credible source (9.1) were coded. When CBT approaches were described along with a specification of a treatment rationale that induced coding 4.2 and 5.1; clarifying relationships of behaviors with antecedents and their health consequences. Descriptions of cognitive restructuring or defusion led to coding 13.2; the framing or re-framing perspectives on behavior to change cognitions or emotions about it. Other regularly coded techniques (k > = 10) were self-monitoring of behavior (2.4), problem solving (1.2), outcome goal setting (1.3), exposure (7.7), and setting and performing graded tasks (8.7).

#### Mode of delivery

Table D in S1 Appendix also contains complete coding results for use of theory (3). It shows that studies described various automated functions of their CBIs, including tailored feedback based on individual progress monitoring (k = 37, 76.1%), an enriched information environment (k = 25, 52.2%), and/or automated follow-up messages (k = 32, 67.4%). Less often, interventions descriptions mentioned (two-way) communicative functions, such as communicating with an advisor through scheduled contact (k = 24), access to an advisor for advice (k = 4), and/or peer-to-peer access (k = 11). Most studies mentioned the Internet (k = 41), followed by e-mail (k = 31), telephone (k = 12), and SMS (k = 7) as supplementary modes.

### Risk of bias

As presented in Table 1, none of the 46 studies were coded low risk of bias within all categories. Selection bias was coded low in 11 studies, meeting the three criteria of random sequence generation, concealment of allocations, and group similarity at baseline. Ten studies were assessed as low risk, while 25 studies were assessed as high risk of attrition bias. Thirty-four studies were classified as having an unclear risk of reporting bias through selective outcome reporting, because a study protocol was either not available or registered after the study was completed. For performance bias, 12 studies scored high risk and 18 studies low risk, which depended on differences in compliance and co-interventions between groups. Only one study scored high risk for detection bias. Four studies were assessed as high risk of bias due to incomplete reporting and analysis according to group allocation, because findings differed between intention-to-treat and complete case analyses. This was unclear if no results of intention-to-treat analyses were reported (k = 11). Table B in S1 Appendix elaborates on the reasons authors agreed upon for assigning high, low, or uncertain risk by criteria.

**Table 1:** Risk of bias assessment by the 7 key categories of the Cochrane risk of bias tool.

First author, year of publication	Selection bias	Attrition bias	Reporting bias	Performance bias	Incomplete analysis according to group allocation	Detection bias	Other bias
Abbott, 2009	high risk	high risk	Unclear	high risk	low risk	low risk	low
Andersson, 2002	Unclear	high risk	Unclear	low risk	Unclear	low risk	high
Andersson, 2003	Unclear	high risk	Unclear	low risk	Unclear	low risk	high
Boer, de, 2014	high risk	high risk	Unclear	Unclear	high risk	low risk	high
Brattberg, 2006	low risk	high risk	Unclear	Unclear	Unclear	low risk	low
Buhrman, 2004	high risk	high risk	Unclear	low risk	Unclear	low risk	low
Buhrman, 2011	low risk	Unclear	Unclear	low risk	low risk	low risk	low
Buhrman, 2013a	high risk	high risk	Unclear	Unclear	low risk	low risk	low
Buhrman, 2013b	Unclear	high risk	Unclear	Unclear	low risk	low risk	low
Buhrman, 2015	high risk		Unclear	high risk	low risk	low risk	low
Camerini, 2012	Unclear	high risk	Unclear	high risk	Unclear	low risk	low
Carpenter, 2012	Unclear	high risk	Unclear	low risk	Unclear	low risk	low
Chiauzzi, 2010	high risk	high risk	Unclear	Unclear	low risk	low risk	low
Davis, 2013	low risk	Unclear	Unclear	high risk	low risk	low risk	low
Dear, 2013	Unclear	low risk	low risk	low risk	low risk	low risk	low
Dear, 2015	low risk	Unclear	low risk	low risk	low risk	low risk	low
Devenini, 2005	Unclear	high risk	Unclear	Unclear	Unclear	Unclear	low
Dowd, 2015	Unclear	high risk	Unclear	high risk	low risk	low risk	low
Everitt, 2013	high risk	Unclear	low risk	high risk	low risk	low risk	low
Hesser, 2012	low risk	low risk	Unclear	low risk	low risk	low risk	low
Hunt, 2009	Unclear	high risk	Unclear	high risk	high risk	low risk	high
Hunt, 2015	Unclear	high risk	Unclear	Unclear	high risk	low risk	high
Janse, 2016	Unclear	low risk	low risk	high risk	low risk	high risk	low
Jasper, 2014	Unclear	low risk	low risk	low risk	low risk	low risk	low
Kaldo, 2008	Unclear	low risk	Unclear	low risk	low risk	low risk	high
Krein, 2013	low risk	low risk		Unclear	low risk	low risk	low
Kristjánsdóttir, 2013	low risk	high risk	Unclear	low risk	low risk	low risk	low
Lee, 2014	Unclear	high risk	Unclear	Unclear	Unclear	low risk	low
Ljotsson, 2010	Unclear	low risk	Unclear	low risk	low risk	low risk	low
Ljotsson, 2011a	low risk	low risk	Unclear	Unclear	low risk	low risk	low
Ljotsson, 2011b	low risk	high risk	Unclear	Unclear	low risk	low risk	low
Lorig, 2008	Unclear	high risk	low risk	Unclear	low risk	low risk	low
Menga, 2014	Unclear	high risk	Unclear	Unclear	Unclear	low risk	high
Moessner, 2014	Unclear	high risk	Unclear	Unclear	low risk	low risk	low
Mourad, 2016	high risk	low risk	low risk	low risk	low risk	low risk	high
Naylor, 2008	low risk	low risk	Unclear	low risk	low risk	low risk	low
Oerlemans, 2011	high risk	Unclear	high risk	low risk	Unclear	low risk	low
Riva, 2014	low risk	low risk	Unclear	Unclear	low risk	low risk	low
Ruehlman, 2012	Unclear	high risk	Unclear	Unclear	low risk	low risk	low
Schulz, 2007	high risk	low risk	high risk	high risk	low risk	low risk	high
Strom, 2000	Unclear	high risk	Unclear	high risk	Unclear	low risk	high
Trompetter, 2015	Unclear	high risk	low risk	high risk	low risk	low risk	low
Vallejo, 2015	Unclear	Unclear	Unclear	low risk	low risk	low risk	high
Weise, 2016	Unclear	low risk	low risk	low risk	low risk	low risk	low
Williams, 2010	Unclear	Unclear	Unclear	low risk	low risk	low risk	low
Wilson, 2015	Unclear	high risk	Unclear	high risk	high risk	low risk	low

The 13 risk of bias criteria of the Cochrane Collaboration Back Review Group were combined into these 7 major categories of the general Cochrane risk of bias tool.

### Meta-analyses

Multiple meta-analyses were conducted for assessing the 20 direct effects of CBIs, which is too much for presenting each here in full detail. Tables F-Y, and Figs A-AB in S1 Appendix contains full information on the direct effect estimates numbered per comparison, outcome type, and time of measurement (passive = 1., active = 2.; and .1 = symptom intensity post treatment, .2 = HRQOL, .3 = functional interference, .4 = catastrophizing, and .5 = depression, .6 - .10 = same subsequent outcomes at follow-up of 6 months or longer). For each estimate, information is given on the *SMD* pooled for all eligible studies with its 95% confidence interval and heterogeneity statistics (I2, P-value). Furthermore, the same statistics are presented for sub-sets of studies with low risk across sources of bias, and for study sets that recruited patients from open or closed populations (sensitivity analyses). In addition, forest plots (providing detailed study level outcome information in a single overview) and funnel plots (visualizing study estimates relative to their sample sizes for detecting potential publication bias) are presented. Table 2 presents (per comparison, outcome type, and time of assessment) the pooled *SMD*s, appurtenant confidence intervals, and heterogeneity statistics and references to the 25% sub-sets of studies with highest and lowest *SMD*s. In the text, a factual summary is given, mentioning key information that is in S1 Appendix and not in Table 2.

**Table 2:** Overview of meta-analyses results for direct effects based on all eligible studies with relevant data.

Outcome [study references]	Effect size estimate		Heterogeneity		Study references	
	SMD*	95% CI	I^2^	P*	High 25% *SMD*	Low 25% *SMD*
*CBI vs*. *passive controls*						
Symptom intensity post* [16, 65, 67–73, 75, 76, 79–81, 83, 85–87, 90, 92, 93, 96, 101, 103, 104, 107, 108]	-.35	-.48 - -.22	65%	< .01	[81, 85–87, 92–94]	[65, 67, 71, 72, 83, 101, 108]
HRQOL post [65, 68, 70–73, 83–86, 92–94, 96]	-.32	-.55 - -.10	70%	< .01	[85, 86, 94]	[65, 83, 92]
Functional interference post [65, 67–73, 75, 76, 79–82, 84, 87, 88, 90, 92–94, 96, 97, 101, 103–108]	-.35	-.45 - -.25	45%	< .01	[68, 70, 72, 80, 84, 97, 106]	[65, 76, 92, 101, 103, 105, 108]
Catastrophizing post [16, 69–73, 75, 76, 79, 80, 82, 84–86, 88, 90, 93, 94, 96, 101, 104–106, 108]	-.41	-.50 - -.31	28%	.1	n.a.*	n.a.
Depression post [65, 67–73, 76, 79, 80, 82–84, 87, 88, 94, 99, 101, 103, 104, 106–108]	-.18	-.28 - -.07	29%	.1	n.a.	n.a.
Symptom intensity f-u* [76, 82, 96, 104]	-.18	-.30 - -.05	0%	.52	n.a.	n.a.
HRQOL f-u [96]	.13	-.02 - .28	/	/	n.a.	n.a.
Functional interference f-u [76, 82, 96, 104]	-.18	-.30 - -.06	0%	.62	n.a.	n.a.
Catastrophizing f-u [76, 82, 96, 104]	-.32	-.47 - -.17	19%	.30	n.a.	n.a.
Depression f-u [76, 82, 104, 109]	-.29	-.48 - -.10	0%	.59	n.a.	n.a.
*CBI vs active controls*						
Symptom intensity post [9, 66, 78, 79, 83, 89, 91, 95, 98, 100, 104]	-.16	-.35 - .02	56%	.01	[9, 104]	[91, 100]
HRQOL post [9, 78, 83, 84, 95, 98]	-.17	-.48 - .14	74%	< .01	[9]	[83]
Functional interference post [9, 66, 79, 84, 88, 89, 91, 98, 104, 105]	-.15	-.27 - -.03	0%	.7	n.a.	n.a.
Catastrophizing post [9, 66, 78, 79, 84, 88, 91, 95, 104, 105]	-.26	-.41 - -.10	21%	.25	n.a.	n.a.
Depression post [66, 84, 88, 89, 91, 95, 104, 105]	-.14	-.37 - .09	47%	.07	n.a.	n.a.
Symptom intensity f-u [88, 91, 95, 98, 104]	-.15	-.40 - .10	60%	.04	[95]	[98]
HRQOL f-u [84, 95, 98]	-.04	-.37 - .30	66%	.05	n.a.	n.a.
Functional interference f-u [84, 88, 91, 98, 104, 105]	-.20	-.44 - .05	56%	.05	[105]	[84]
Catastrophizing f-u [84, 88, 91, 95, 105]	-.27	-.56 - .02	53%	.08	n.a.	n.a.
Depression f-u [84, 88, 91, 95, 104, 105]	-.31	-.78 - .16	85%	< .01	[105]	[84]

*SMD = Standardized Mean Difference

CI = Confidence interval; *P* = P-value for Chi^2^ test of Tau^2^ (heterogeneity); post = outcome measurement shortly after treatment; HRQOL = Health-related Quality Of Life; n.a. = not applicable, because the degree of heterogeneity was statistically insignificant or unimportant, or because fewer than 4 studies reported outcome information in this category; f-u = measured at follow-up

#### Computer-based interventions versus passive controls

After treatment, observed differences between CBI and control group means (*SMD*s) were significant and small- to medium-sized, ranging from -.18 for depression to -.41 for catastrophizing. For functional interference, somatic symptom intensity, and HRQOL heterogeneous estimates between studies were found (in the range for classification as ‘modest’ to ‘substantial’), which could be further explored to its sources. Sub-group analyses of study sub-sets by risks of bias only showed significantly stronger (*SMD* = -.49 - -.53) functional interference outcomes after CBI versus controls in trials with low risk due to attrition (χ^2^ = 7.97, p < .01) and performance (χ^2^ = 5.10, p = .02). Inspection of funnel plots, most clearly those for (post treatment) somatic symptom intensity and HRQOL, showed a lack of observations at the bottom-right corner (small studies with negative effect estimates) unlike the bottom-left corner (small studies with positive estimates).

At 6 or more months after treatment, small significant effect sizes (*SMD* = -.18 -—.32) were maintained for all outcomes except for HRQOL (k = 1). There were too few studies available (1 < k < 6) for sensitivity analyses on follow-up results.

#### Computer-based interventions versus active controls

In comparisons of CBIs with active control groups, small positive significant outcome differences (*SMD* = -.15 - -.26) were only found for catastrophizing and functional interference outcomes after treatment. For both estimates, the between study heterogeneity estimate (I^2^) was in the range of ‘not important’. Within this comparison type, there were only enough data (k = 10) to observe that the estimated effect on catastrophizing was significant and positive (*SMD* = -.33, 95% CI = [-.49, -.17]) within the sub-set of studies assessed with low risk of selection bias (k = 5). At follow-up, no significant differences between CBI and active controls were observed (3 < k < 6, *SMD* = -.04 - -.031). Significant heterogeneity in the range of ‘modest’ or ‘substantial’ was observed for symptom intensity and HRQOL at both times of assessment, and for functional interference and depression at follow-up. Only for symptom intensity at post there were enough studies (k = 11) for further exploration of sources of heterogeneity. For depression, heterogeneity was accompanied by an apparently outlying observation from a small study [105]. Further, researchers have not noticed anything unusual in the funnel plots of the smaller numbers of studies within this comparison.

#### Patient characteristics of effective computer-based interventions

Table Z in S1 Appendix presents results of the first (intermediate) step in exploring patient and intervention factors. Herein 6 table columns represent study sets: studies with passive and active comparison types and studies with the 25% highest and 25% lowest effect sizes (*SMD*s) within meta-analyses with sufficient studies (k = 10) and significant heterogeneity (somatic symptoms at post and follow-up, and HRQOL and functional interference at post). Rows list control conditions and characteristics of patients and interventions. Cells contain corresponding statistics. Table AA in S1 Appendix completely presents the 42 sub-group analyses that were chosen to be conducted, along with their corresponding sub-group operationalization and test statistics (χ^2^, *P*-value, and I^2^). A full overview of associations between study characteristics is available upon the author’s request. Here, significant findings are mentioned.

**Fig 3:**
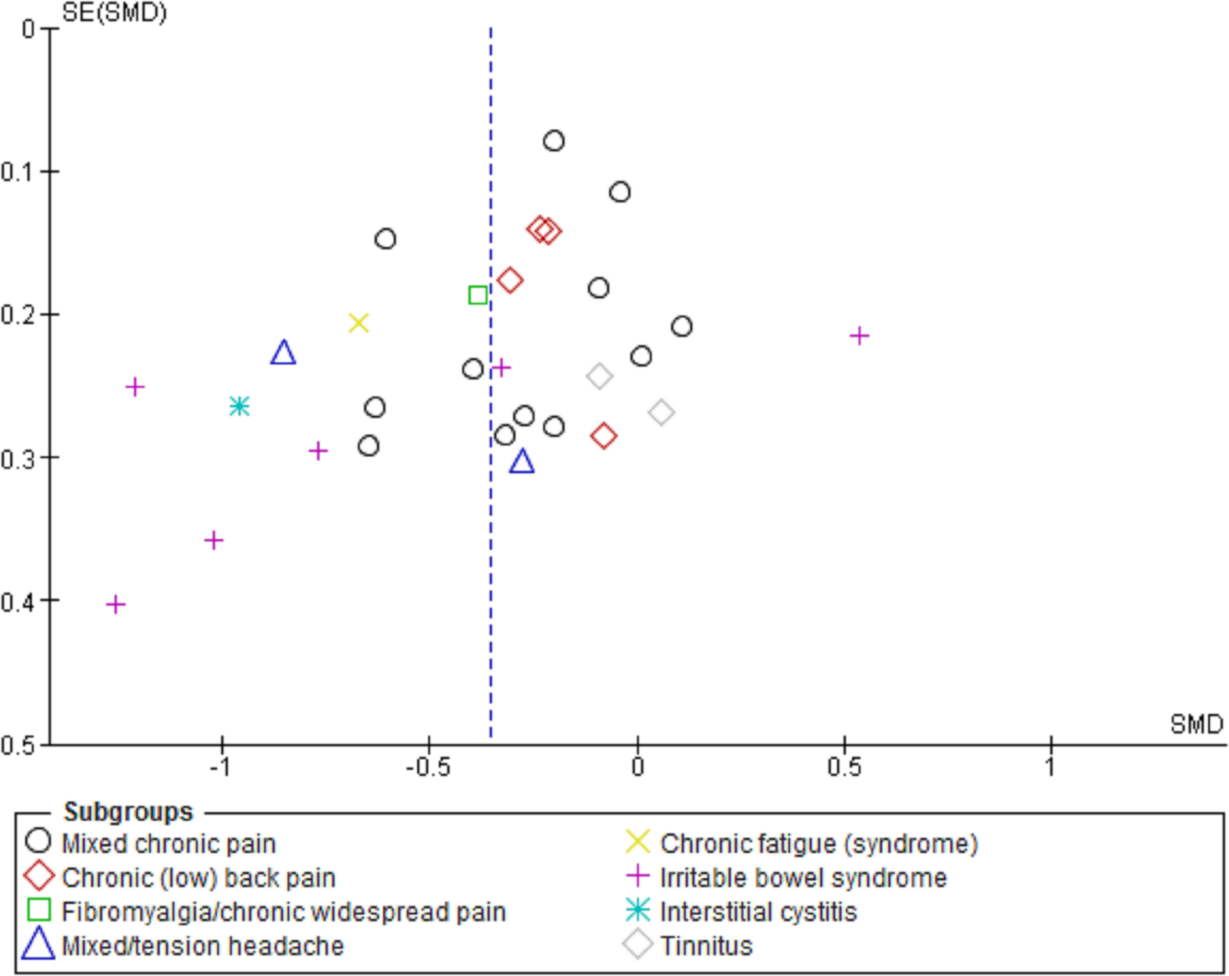
Funnel plot for symptom severity scores post treatment by various patient conditions. *SE* = Standard Error, *SMD* = Standardized Mean Difference.

Between the 8 different sub-groups by patient conditions, different *SMD*s were seen for somatic symptoms after CBIs in comparison with passive controls (χ^2^ = 15.62, p = .03). When comparing only the sub-group of IBS studies (k = 6) with mixed CP studies (k = 12) within the same meta-analysis, higher estimates are observed after also excluding one study on IBS patients with outlying (negative) results (k = 17, χ^2^ = 9.60, p < .01; Fig 3) [83]. For studies with a relatively lower average participant age (< 42.5 years), higher estimates were seen (at post) for somatic symptoms and HRQOL (χ^2^ = 11.45–15.11, p < .01). Estimated effects on somatic symptoms (at post) were higher in study sub-groups with higher average proportions of female study participants (3 groups split by 2/3 and 4/5, k = 29, χ^2^ = 9.19, p < .01), or with a higher proportion of participants with a completed tertiary education (2 groups split by 40%, k = 17, χ^2^ = 5.46, p = .02). Estimated CBI effects on functional interference (after treatment, versus passive controls) were higher for a subset of studies (k = 4) with higher depression scores at baseline (Hospital Anxiety and Depression Scale > 7) (k = 11, χ^2^ = 5.32, p = .02), and a subset (k = 3) with more than 50% absenteeism among study participants (k = 11, χ^2^ = 3.73, p = .05). Finally, even without removal of a statistical outlier [83], estimates for a sub-group of studies (k = 19) with explicit participant computer literacy selection criteria were lower than for studies (k = 10) with unclear or implicit criteria (k = 29, χ^2^ = 4.79, p = .03).

#### Intervention characteristics of effective computer-based interventions

**Fig 4:**
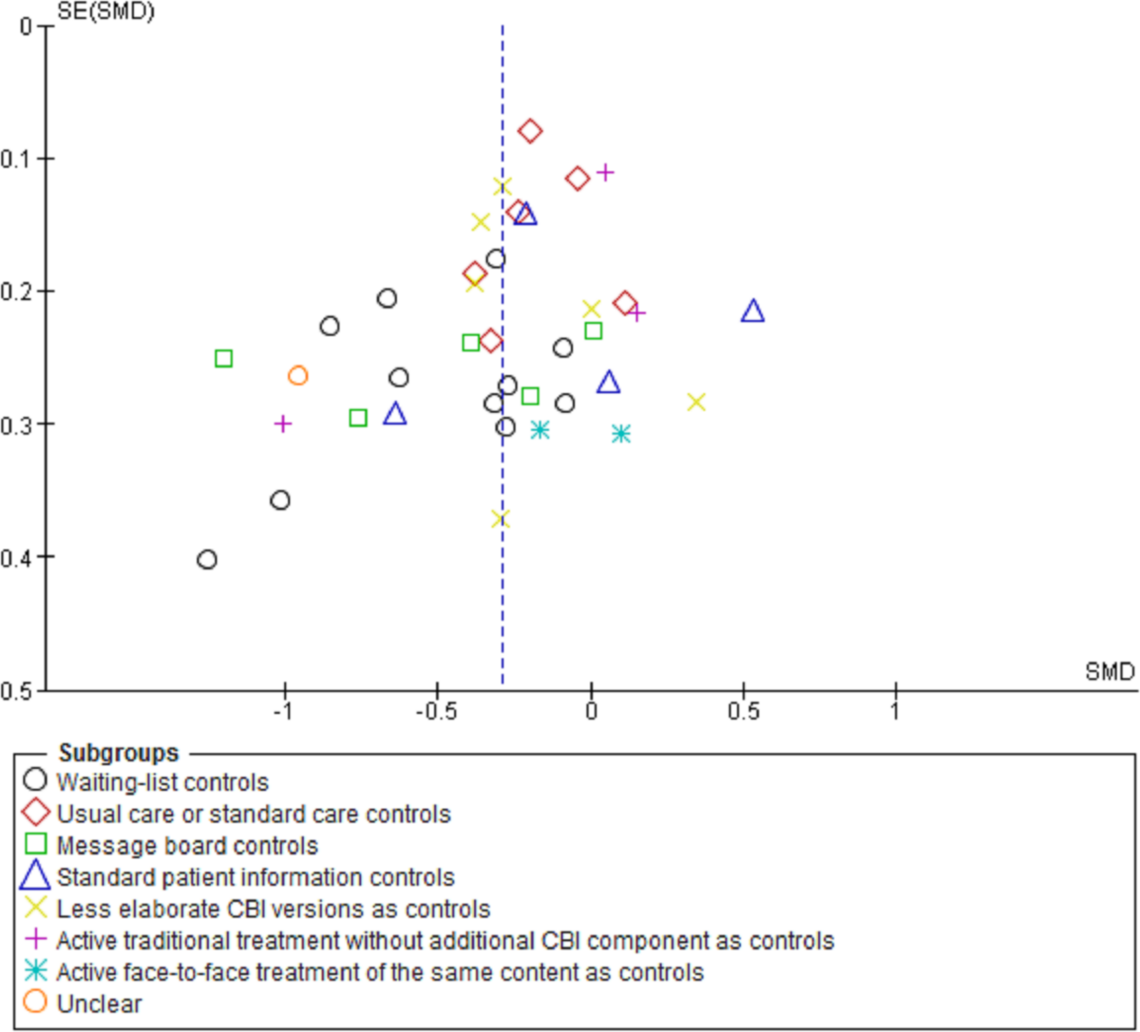
Funnel plot for symptom severity scores post treatment by various types of control groups. *SE* = Standard Error, *SMD* = Standardized Mean Difference. Comments: The meta-analysis presented here included the results for active comparisons (not the passive ones) from Trompetter et al. (2015) and Dear et al. (2015) to avoid double entries. Online discussion was facilitated for control group participants while being on a waiting list for receiving the experimental CBI.

Efficacy estimates also varied by several sub-groups of intervention characteristics. Differences in *SMD*’s by the 4 types of passive control groups were found for somatic symptom, HRQOL, and functional interference outcomes at post treatment (χ^2^ = 12.79–22.73, p = < .005. Fig 4). More specifically, efficacy estimates of studies on comparisons of CBIs with care as usual (*SMD* = -.04 - -.17) instead of waiting list controls (*SMD* = -.79 - -.43) were smaller (14 < k < 21, χ^2^ = 10.78–11.06, p = < .001). Furthermore, differences of *SMD*s in functional interference outcomes were found by the presence of guidance or its levels of professionalism (k = 30, χ^2^ = 9.84, p = .02). Effects were generally small when guidance was absent (k = 11, *SMD* = -.24), larger at master’s level (k = 7, *SMD* = -.38), and largest at clinical level (k = 11, *SMD* = -.49; χ^2^ = 9.84, p = .02). Post treatment *SMD*s in HRQOL for the sub-group of studies that reported the used of theory for the selection of intervention techniques (k = 7, *SMD* = -.62) versus the studies that did not (k = 7, *SMD* = -.08) were relatively higher (k = 14, χ^2^ = 5.79, p = 0.02). For studies for which “exposure” (7.7) was coded versus those for which it was not, *SMD*’s in (post-treatment) somatic symptom (*SMD* = -.67 versus -.26) and functional interference (SMD = -.50 versus -.28) were higher (χ^2^ = 3.72–6.26, p = < .05). For somatic symptom outcomes, it also appeared that higher *SMD*s between CBI and (both passive and active) controls were higher for sub-groups of studies that reported fewer (subsequently less than 5 or 2) rather than more modes of delivery (χ^2^ = 5.11–6.34, p = < .04). *SMD*s in somatic symptom outcomes (at post) were higher (*SMD* = -.52 versus -.18) in studies reporting a 50% or higher CBI completion rate (k = 20, χ^2^ = 4.55, p = .03). Completion rate, on its turn, was associated with the use of occasional (instead of absent or scheduled) reminders (one-way ANOVA: F = 3.06, p = .045).

## Discussion

### Summary of evidence

This study questioned (1) the short and long-term efficacy of CBIs compared with passive and active controls for (self-reported) outcomes in patients with CP or FSS, as well as (2) patient and (3) intervention factors by which efficacy is high versus low. Generally, small effects were found when CBI was compared to passive controls. But when CBI was compared to active control groups, no significant differences in treatment effects were found. Small levels of efficacy were maintained for 6 months or longer. Strength of evidence varied by follow-up terms and type of comparison groups. Additionally, explorative analyses provided several (interdependent) possible patient and intervention characteristics that showed marked differences in treatment effects.

First, regarding efficacy, a small positive average effect of CBI is found in comparison with passive controls across all outcomes at post-treatment (i.e. somatic symptom intensity, HRQOL, functional interference, catastrophizing, and depression). CBIs predominantly included typical contents of CBT. This agrees with the up-to-medium sized effects observed in previous meta-analyses on the efficacy of Internet CBT in populations with CP [1, 14] and mental or chronic somatic symptoms at large [22, 32, 110, 111]. Additional meta-analytic evidence is presented (except for HRQOL) in support of the hypothesis that CBI efficacy is retained for 6 months or longer. This strengthens the previous suggestion that CBI effects would last at least up to three months [14]. Moreover, similar meta-analytic results were previously found for depression [110]. Our meta-analyses do not suggest that CBIs have additional effects when complementing (during or after) face-to-face delivered multidisciplinary programs (k = 3), or when substituting traditional (group) formats of CBT (k = 4). These findings concur with previous studies that suggested equivalence between computer- or group-based CBT across psychiatric and chronic somatic disorders [14, 18, 34]. In sum, CBIs offering complementary behavioral change content have, on average, small and prolonged effects on self-reported health in patients with CP or FSS.

Secondly, this study explored characteristics of patients with CP or FSS for whom CBIs are most and least effective. Different characteristics of patients included in studies were sometimes associated with significantly higher or lower CBI effects on somatic symptom, HRQOL, and functional interference outcomes. Even though effect estimates (i.e., for somatic symptom outcomes) could not be considered equal across different CP and FSS diagnoses, no particular diagnosis stood out. Unfortunately, the number of studies per diagnosis was too small to perform 1 on 1 comparisons between all diagnoses (all k < 5 except IBS; k = 6). For IBS, effect estimates were relatively high (medium sized), but one study had a deviating low effect, so that a difference with other diagnostic groups cannot be suggested on statistic grounds. This study by Everitt et al. (2013) differed from other IBS studies (but not from other included studies) by setting (“closed”) patient factors (higher age and lower education level), intervention factors (lower compliance), and risk of bias criteria (low risk of reporting bias) [83]. It was also the only study that tested combinations of CBI and drug treatments that, according to the authors, could have affected patient expectations. Mechanisms of heterogeneous effects of IBS (self) management interventions have been largely unclear [36, 112]. Future research should clarify whether distinctive CBI efficacy has to do with differentiating characteristics of IBS, or other differences in interventions studied, patients, and/or context.

Exploration of other patient factors of CBI efficacy resulted in positive findings by demographics and health status. More favorable post-treatment somatic symptoms and HRQOL outcomes of CBI versus passive controls were observed for adult patient samples with a relatively lower (adult) age. Previous studies (process- and meta-analyses) of CBIs or self-help also found more favorable outcomes in relatively younger patients with chronic somatic or psychiatric conditions (for somatic symptoms and HRQOL [96, 113], and for functional interference, cognition, and depression [37, 39, 40]). Thus, several powerful studies have now suggested (subtly) better effects of CBI in patients of relatively young adult age. Furthermore, this study found higher estimates of average CBI efficacy for somatic symptom intensity by higher proportions of females and highly educated patients, and for functional interference by initial depressed mood (mean HADS > 7) and sick leave (> 50% of the sample). Higher efficacy estimates and proportions of highly educated patients came with absent reporting of explicit eligibility criteria for computer literacy. Authors of included trials expressed their uncertainty about whether outcomes were influenced by their methods of participant inclusion and could only refer to a single trial with depressed patients to contest this [88, 114]. Some previous studies also reported better HRQOL outcomes by gender [37], and better depression and/or functional interference outcomes by higher education, depression, and disability [35, 37, 39, 113]. In all, this and previous studies have been inconsistent about the significance, but consistent about the characteristics of CP or FSS patients for whom CBIs are more or less effective. This should not be seen as a reason to offer CBI only to certain patients, because effect sizes are significant for several outcomes in any sub-group. Rather, sensitivity to individual differences in responding may help to achieve the full potential of CBI in practice.

Third, explorations provided insight into characteristics by which CBI is most or least effective. Overall, this study shows that experimental CBIs were quite similar in terms of theoretical basis, behavioral change techniques, and delivery modes. Some aspects that varied between CBIs, including the use of theory for selecting intervention techniques, exposure techniques, and a limited amount of delivery modes were associated with relatively higher effects. Efficacy estimates in this study are relatively high for included studies that referred to third-wave CBT-models (Mindfulness and Acceptance Commitment Therapy) (i.e., for functional interference), but there was no statistically significant moderation effect by different theoretical (CBT) models mentioned. A previous study on the efficacy of face-to-face delivered third-wave therapies in CP suggested that it is a good alternative rather than superior to ‘‘traditional” CBT models [115]. This applies to CBI as well. Furthermore, efficacy estimates for functional interference increased with the presence and level of training of care providers. The importance of professional guidance was stressed before in reviews on CBI for health conditions and depression, and self-help for chronic back pain [34, 110, 116]. Efficacy in somatic symptom outcomes was raised by compliance, which appeared to increase with occasional reminders. Therefore, proficient guidance and compliance feedback may improve efficacy for some outcomes. No significant moderation was found by intervention duration, but efficacy seems highest in studies with a program duration of 7 to 10 weeks. Previous meta-analyses were inconsistent in this regard [32, 34, 117]. It seems reasonable to expect that users need substantial time to process contents that are relevant to them and are likely to lose interest when a program takes too long to complete [117]. Such differences would inform decision makers, clinicians, and developers about design qualities by which CBIs are comparatively efficacious or plausibly equivalent to active intervention through conventional means (face-to-face), i.e. if inaccessible or too costly.

### Strengths and limitations

Study limitations were, first, that grey literature had not been searched and authors were not contacted for missing data. Thereby, a risk has been taken that relevant (negative) results are neglected. Secondly, moderator analyses were explorative and intended to inform hypotheses formulation. Since there were statistically significant associations between computer-literacy selection criteria, age, education level, IBS-diagnosis and reporting of 'exposure' techniques, it should be emphasized that subgroup analyses are not suitable for unraveling (spurious) relationships amongst (heterogeneous) outcomes and its factors. These exploratory analyses also overlooked sensitivity by risk of bias and did not enable to control factors for one another (as in meta-regression). Third, the performance of sensitivity analyses by extracted information about generalizability is not a well-established procedure. It is hindered by the novelty of reporting standards for E-health (since 2011) [46]. Because of understandable limitations in reporting at this time, authors agreed that converting reporting items (e.g. participant compensation, co-interventions, numbers and research engagement of providers) to classifications was too ambiguous. Nonetheless, this study has several particular strengths. An extensive search strategy was used, creating a good chance that found studies are exhaustive with regard to eligible full-bodied, peer-reviewed, and published articles. The pooling of outcomes for multiple “overlapping” adult patient conditions increased the number of included studies and meta-analytic power. Classification of intervention content with uniform and empirically supported taxonomies was applied in a consistent, transparent, and independent way on the basis of intervention descriptions in study articles and research protocols [59, 60]. Finally, this is the first study in the field, by knowledge of the authors that included meta-analyses by independent assessment of risk of bias criteria, behavioral intervention content, and relevant items for generalizability.

It is due to these strengths that plenty of information is provided on outcome-level strengths and weaknesses. Regarding our first research question, strength of evidence for short-term CBI outcomes in comparison with passive controls is supported by the robustness of sensitivity analyses across most sources of bias risk. Nonetheless, studies of low risk of bias were minorities across criteria. Concerns for placebo effects also remain [118], because blinding of participants and staff are generally impractical, and different effect estimates were found by variety in control group interventions and their credibility as attentional placebos. Furthermore, inspected funnel plots did not fully contradict risk of reporting and/or publication bias. This was seen in a previous meta-analysis on self-help for MUPS, but not in meta-analyses on Internet CBT for CP [1, 118]. Therefore, explanations for funnel plot shapes could also be found in other methodological factors, such as the scale of CBI deployment (studies from the United States were typically larger scaled, less often professionally guided and reported relatively lower functional interference outcomes than European studies) [1, 45, 118]. Evidence for internal validity should be considered weak for longer follow-up terms or active comparison groups, because sensitivity analyses were undermined by the scarcity of high quality studies. Indications that a significant degree of effectiveness might be less certain when CBIs are offered to patients in closed clinical or work setting do not contribute to external validation (in routine deployment).

Regarding the second and third research question, important patient and/or intervention factors may be omitted that primary studies did not measure or measured by using different instruments (e.g., previous observations of better effects by higher levels of initial somatic symptoms and self-efficacy could not be replicated [36, 39, 113, 119]). In addition, the use of taxonomies for coding behavioral intervention content could only have led to valid data in so far as accurate descriptions were available. Moreover, findings on relationships between CBI content and outcomes are limited by lacking information about fidelity, despite that standardized delivery is considered to be an important strength of CBI [107, 120, 121]. Overall, the evidence for patient and intervention factors of the heterogeneous efficiency of CBI in some outcomes remains weak.

### Conclusions

In general there is a minority of decent quality information in support of a small positive average effect of CBI compared to passive control conditions on relevant (subjective) outcomes in patients with CP or FSS. There is weaker evidence that effects of CBIs last for 6 months or longer and similar to 'traditional' active treatment conditions. Evidence on CBIs complementary to active treatments are to scarce and diverse to draw conclusions. Therefore, the clinical relevance of CBI effects is generally limited for many individual patients with CP or FSS. Moreover, no certainty can be given that effects are generalizable to patients receiving CBIs in work or clinical settings.

Furthermore, there are indications that CBIs that facilitate compliance and “exposure” through specific delivery methods and expert guidance work best for relatively young, highly educated female patients with depressed mood choosing CBIs. However, which of these interdependent patient and intervention factors is decisive (and why) is not clear. More evidence is needed in support of indications that effects on other outcomes (symptom intensity, quality of life, and functional interference) can vary in consistency and strength, depending on whether interventions include theory-based change techniques, “behavioral exposure” specifically, or guidance by (schooled) professionals, or depending on whether (self-selecting) patients are younger, female, highly educated, absent from work, and/or experience more depressed mood. CBIs may not be more or less effective for emotional functioning (catastrophizing and depressed mood outcomes) with certain intervention or patient characteristics. More in-depth explanation is needed to better understand these factors across settings. On the basis of such information, clinicians and policy makers can improve decisions concerning CBIs in development, tailoring, quality assessment, and allocation to patients. Ideally, individual patients who are offered a CBI in regular patient care will get better chances of experiencing clinically relevant outcomes.

### Future research

Ideally, the efforts of this study are continued by enrichment and refinement of the extracted data for updates. This could be done by collecting information firsthand from authors on intervention factors in compliance with the standard taxonomies and reporting guidelines. Tweaking the search string for improving balance between sensitivity and length would also be helpful. A network meta-analysis of the data could also provide more insight into the relative effectiveness of CBIs in relation to alternatives and each other. However, most progress may be achieved with additional primary studies and embedded process analyses. Future trials should focus on methodological quality, select common measurement instruments, investigate and report completely on selection processes during the recruitment stage, consider more information about patients related to self-selection (e.g. socio-economic status, self-efficacy in general or for using computers), include follow-up terms of at least 6 months, compare active interventions 1-on-1 (i.e., Internet CBT with vs. without exposure techniques), report intervention features and fidelity transparently and uniform. Finally, research should better explain under which conditions individual patients with CP or FSS have a better chance of achieving clinically relevant benefit from behavioral change through CBI than small average group effects imply.

## Supporting information

S1 Checklist PRISMA. (PDF)

S1 Appendix Additional details on methods and results. (PDF)

S1 Protocol PROSPERO. (PDF)
