# Supplementary material for: The effectiveness of various computer-based interventions for patients with chronic pain or functional somatic syndromes: A systematic review and meta-analysis
Source: PLoS One. 2018 May 16;13(5):e0196467. doi: 10.1371/journal.pone.0196467 (PMC5955495; doi:10.1371/journal.pone.0196467)
Supplement: S1 Appendix — (PDF) [file pone.0196467.s002.pdf]

S1 Appendix: Additional details on methods and results

Table of Contents

Table A: Search string for EMBASE & MEDLINE via OVID 2
Table B: Assessments of high (red), unclear (orange), or low (green) risk of bias by all criteria 5
Table C: Outcome measures extracted by studies for each outcome category 7
Table D: Coding results for use of theory (1), individual BCTs (2), and modes of delivery (3) 9
1. Use of theory 9
2. Behavioral change techniques (BCTs) 11
3. Modes of delivery 13
Overview of meta-analyses; *SMD*s, 95% confidence intervals, heterogeneity statistics, forest plots and funnel plots), and sensitivity analyses 14
Comparison 1: Computer-based interventions versus passive controls (tables F-O, figs A-N) 14
Outcome 1.1: Symptom intensity (post) 14
Outcome 1.2: Health-related Quality Of Life (post) 17
Outcome 1.3: Functional interference (post) 20
Outcome 1.4: Catastrophizing (post) 23
Outcome 1.5: Depression (post) 26
Outcome 1.6: Symptom intensity (6 or more months at follow-up) 29
Outcome 1.7 HRQOL (6 or more months at follow-up) 30
Outcome 1.8 Functional interference (6 or more months at follow-up) 31
Outcome 1.9 Catastrophizing (6 or more months at follow-up) 32
Outcome 1.10: Depression (6 or more months at follow-up) 33
Comparison 2: Computer based versus active control (tables P-Y, and figs O-AB) 34
Outcome 2.1: Symptom intensity (post) 34
Outcome 2.2: Health-related Quality Of Life (post) 37
Outcome 2.3: Functional interference (post) 40
Outcome 2.4: Catastrophizing (post) 43
Outcome 2.5: Depression (post) 46
Outcome 2.6: Symptom intensity (6 or more months at follow-up) 49
Outcome 2.7: HRQOL (6 or more months at follow-up) 50
Outcome 2.8: Functional interference (6 or more months at follow-up) 51
Outcome 2.9: Catastrophizing (6 or more months at follow-up) 52
Outcome 2.10: Depression (6 or more months at follow-up) 53
Table Z: Characteristics of sub-sets of studies with the 25% highest and 25% lowest SMD estimates 54
Table AA: Overview of sub-group analyses 56

Table A: Search string for EMBASE & MEDLINE via OVID

<patient condition>

1 Somatoform Disorder/

2 Psychosomatics/

3 Neurasthenia/

4 somati#ation.ti,ab.

5 somatoform.ti,ab.

6 neurasthen$.ti,ab.

7 neurasthen$.ti,ab.

8 psychophysiologic$.ti,ab.

9 psychosomat$.ti,ab.

10 psychogen$.ti,ab.

11 (non organic$ or nonorganic$).ti,ab.

12 (unexplain$ adj1 medical$).ti,ab.

13 (unexplain$ adj1 (sympt$ or problem$ or condition$ or complain$)).ti,ab.

14 ((non specific or nonspecific) adj2 (sympt$ or problem$ or condition$ or complain$)).ti,ab.

15 ((unexplain$ or inexpl$) and (health$ or medical$ or physical$) and (sympt$ or problem$ or condition$ or complain$)).ti,ab.

16 (high utilis$ or high utiliz$).ti,ab.

17 (functional somatic adj2 (sympt$ or syndr$)).ti,ab.

18 Fibromyalgia/

19 fibromyalgi$.ti,ab.

20 chronic widespread pain.ti,ab.

21 Fatigue Syndrome, Chronic/

22 fatigue syndrome.ti,ab.

23 ((non cardiac or noncardiac or non specific or nonspecific) adj2 chest pain).ti,ab.

24 NCCP.ti,ab.

25 Irritable Colon/

26 (IBS or irritable bowel syndrome$).ti,ab.

27 multiple chemical sensitivity.mp.

28 idiopathic environmental intolerance.ti,ab.

29 Premenstrual Syndrome/

30 (premenstrual adj2 (syndrome$ or tension$)).ti,ab.

31 ((non ulcer nonulcer or functional) adj2 dyspepsia).ti,ab.

32 exp Cumulative Trauma Disorders/

33 cumulative trauma disorder$.ti,ab.

34 repe$ strain injur$.ti,ab.

35 ((tension type or idiopathic or psychogenic) adj2 headache$).ti,ab.

36 Temporomandibular Joint Disorders/

37 ((temporomandibular joint or TMJ) adj2 (disease$ or disorder$ or dysfunction$)).ti,ab.

38 HYPOCHONDRIASIS/

39 NEUROCIRCULATORY ASTHENIA/

40 (somati#ing or somati#ed or somatic symptom$ or somatic syndrome$ or symptom syndrome$ or multisomat$ or hypochondria$).ti,ab.

41 ((medic$ adj3 (unexplain$ or inexplic$)) or unexplained symptom$).ti,ab.

42 (((frequent or high) adj1 attend$) or high utili#er$ or repeat$ present$).ti,ab.

43 functional symptoms.ti,ab.

44 reattribution.ti,ab.

45 exp ABDOMINAL PAIN/

46 stomach ache$.ti,ab.

47 exp BACK PAIN/

48 COLONIC DISEASES, FUNCTIONAL/

49 CYSTITIS, INTERSTITIAL/

50 painful bladder syndrome.ti,ab.

51 urethral syndrome.ti,ab.

52 cardiac neuros$.ti,ab.

53 ((non cardiac or noncardiac or non-cardiac) adj chest pain).ti,ab.

54 ((nonorganic or non organic or non-organic) adj pain).ti,ab.

55 effort syndrome.ti,ab.

56 DIZZINESS/
57 myalgic encephalomyel$.ti,ab.
58 ((post viral or postviral or post-viral) adj (fatigue or syndrome)).ti,ab.
59 exp HEADACHE/
60 exp HEADACHE DISORDERS/
61 exp HYPERVENTILATION/
62 exp HYSTERIA/
63 Briquet* syndrome.ti,ab.
64 IRRITABLE BOWEL SYNDROME/
65 MULTIPLE CHEMICAL SENSITIVITY/
66 exp PELVIC PAIN/
67 PSYCHOPHYSIOLOGIC DISORDERS/
68 (psychalgia or psychogenic or psychoseizure$ or psychosomatic).ti,ab.
69 TEMPOROMANDIBULAR JOINT DYSFUNCTION SYNDROM/
70 or/1-69

<Intervention>

71 exp COMPUTER/ or exp MICROCOMPUTER/ or exp INTERNET/ or INTERNET-PROTOCOL/ or LOCAL-AREA-NETWORK/ or COMPUTER-NETWORK/ or MEDICAL-INFORMATICS/ or EDUCATIONAL-TECHNOLOGY/ or AUDIOVISUAL-EQUIPMENT/ or DECISION-MAKING/ or DECISION-SUPPORT-SYSTEM/ or DECISION-TREE/ or DECISIONTHEORY/ or COMPUTER-PROGRAM/ or exp TELECOMMUNICATION/ or exp MULTIMEDIA/ or COMPACT-DISK/ or COMPUTER-ASSISTED-THERAPY/ or COMPUTER-PROGRAM/ or HUMAN-COMPUTER-INTERACTION/ or COMPUTER-INTERFACE/ or COMPUTER-NETWORK/ or ONLINE-SYSTEM/ or ONLINE-SYSTEM/ or MEDICAL-INFORMATICS/ or MOBILE-PHONE/ or COMPUTER-GRAPHICS/ or VIRTUAL-REALITY/ (561689)

72 (COMPUTER* or INTERNET or CD-ROM or CDROM or (CELLULAR adj PHONE) or (CELLULAR adj TELEPHONE) or (MOBILE adj PHONE) or (MOBILE adj TELEPHONE) or ((ELECTRONIC adj MAIL) or EMAIL or E-MAIL) or HYPERMEDIA or (VIDEO adj GAME*) or (VIDEO adj RECORDING) or DVD or (WORLD adj WIDE adj WEB) or WORLD-WIDE-WEB or (WORLD-WIDE adj WEB) or (WORLDWIDE adj WEB) or (WEB adj SITE) or WEBSITE or (ONLINE or ON-LINE) or (CHAT adj ROOM) or CHATROOM or BLOG* or WEB-LOG* or WEBLOG* or (BULLETIN adj BOARD*) or BULLETINBOARD* or MESSAGEBOARD* or (MESSAGE adj BOARD*) or (INTERACTIVE adj HEALTH adj COMMUNICATION*) or (INTERACTIVE adj (TELEVIS* or VIDEO or TECHNOLOGY or MULTIMEDIA)) or E-HEALTH or EHEALTH or EHEALTH or (ELECTRONIC adj HEALTH) or (CONSUMER adj HEALTH adj INFORMATIC*) or (VIRTUAL adj REALITY) or (SURF* and (WEB* or INTERNET))).ti. (115754)

73 71 or 72

74 exp Self Care/ or exp Patient Education/ or exp Patient Participation/ or exp Consumer/ or exp EMPOWERMENT/ or exp REHABILITATION/ or exp Daily Life Activity/ or exp Social Support/ or exp Coping Behavior/ or exp Behavior Therapy/

75 (((self or symptom*) adj (care or help or manag* or directed or monitor* or efficacy or admin*)) or ((health or patient*) adj2 (educat* or information)) or ((patient* or consumer*) adj part*) or (holistic or wholistic) or rehab* or (activit* adj2 daily adj living) or (social adj (support or network*)) or (support adj system*) or (psychologic* adj (adjust* or adapt*)) or (cope or copes or coping) or (adapt* adj behav*) or (behav* adj (thera*P* or intervention*))).ti.

76 74 or 75

77 exp Abreaction/ or abreaction.mp. or exp Adaptation, Psychological/ or (Psychological adj Adaptation).mp. or exp aromatherapy/ or aromathera*P*.mp. or exp art therapy/ or exp autogenic training/ or (autogenic adj train*).mp. or exp autosuggestion/ or exp Aversive Therapy/ or exp behavior therapy/ or exp bibliotherapy/ or bibliothera*P*.mp. or exp biofeedback, psychology/ or Biofeedback.mp. or exp catharsis/ or catharsis.mp. or exp conditioning/ or conditioning.mp. or exp conditioning, classical/ or (classical adj conditioning).mp. or exp conditioning, operant/ or (operant adj conditioning).mp.

78 exp Cognitive Therapy/ or exp color therapy/ or exp Counseling/ or counsel?ing.mp. or exp Couples Therapy/ or exp crisis intervention/ or (crisis adj intervention).mp. or exp dance therapy/ or exp Desensitization, Psychologic/ or Desensiti?ation.mp. or exp Early Intervention/ or Early Intervention.mp. or exp Exercise Therapy/ or exp Eye Movement Desensitization Reprocessing/ or (Eye Movement adj2 (Desensiti?ation or Reprocessing)).mp. or exp Family Therapy/ or exp feedback, psychological/ or exp free association/ or (free adj association).mp. or exp gestalt therapy/ or exp hypnosis/ or hypnosis.mp. or exp imagery/ or imagery.mp. or exp implosive therapy/ or exp Intervention Studies/ or exp marital therapy/ or exp meditation/ or meditation.mp. or exp milieu therapy/ or exp music therapy/ or exp nondirective therapy/

79 exp play therapy/ or exp psychoanalytic therapy/ or exp psychodrama/ or psychodrama.mp. or exp psychotherapeutic processes/ or (psychothera*P* adj process*).mp. or exp psychotherapy/ or psychothera*P*.mp. or exp psychotherapy, brief/ or exp Psychotherapy, Group/ or exp psychotherapy, multiple/ or exp psychotherapy, rational-emotive/ or exp reality therapy/ or exp residential treatment/ or (residential adj treatment?).mp. or exp socioenvironmental therapy/ or exp suggestion/ or exp systems theory/ or exp therapeutic community/ or exp transactional analysis/ or (transactional adj analysis).mp.

80 ((Acceptance commitment or Art or Assertive or autosuggestion or Aversive or Behav$ or Client cent$ or Cognitive or Colo?r or Compassion$ or couples or dance or Directive or Exercise or Family or gestalt or Human Givens or Humanistic or

implosive or Interpersonal or marital or mentali?ation or milieu or music or nondirective or patient cent$ or play or psychoanalytic or rational? emotive or reality or socio?environmental or suggestion or systemic or systems or therapeutic community) adj2 therap$).mp.
81 (Behav$ modification or Compassionate Mind Train$ or Emotional freedom tapping or Flooding or Mindfulness or Psychodynamic or Rewind technique? or Stress manag$).mp.
82 74 or 75 or 77 or 78 or 79 or 80 or 81

<Study type:>
83. COMPARATIVE-STUDY/
84. FOLLOW-UP/
85. PROSPECTIVE-STUDY/
86 (CONTROL$ or PROSPECTIV$ or VOLUNTEER$).ti,ab.
87. factorial$.ti,ab.
88. random$.ti,ab.
89. (crossover$ or cross over$ or cross-over$).ti,ab.
90. placebo$.ti,ab.
91. (doubl$ adj blind$).ti,ab.
92. (singl$ adj blind$).ti,ab.
93. assign$.ti,ab.
94. allocat$.ti,ab.
95. volunteer$.ti,ab.
96. crossover procedure.sh.
97. double blind procedure.sh.
98. randomized controlled trial.sh.
99. single blind procedure.sh.
100. (CONTROL$ or PROSPECTIV$ or VOLUNTEER$).ti.
101. CONTROLLED-CLINICAL-TRIAL/
102. CLINICAL-TRIAL/
103. exp RANDOMIZATION/
104. (CLINIC$ adj25 TRIAL$).ti,ab.
105. (COMPARATIVE adj STUDY).ti.
106. exp evaluation/
107. ((time adj series) or (pre test or pretest or (post test or posttest))).tw.
108. exp animal/ or nonhuman/ or exp animal experiment/
109. exp human/
110. or/83-109
111. 108 and 109
112. 108 not 111
113. 110 not 112
114. 70 and 73 and 82 and 113

Table B: Assessments of high (red), unclear (orange), or low (green) risk of bias by all criteria

"High risk" or "low risk" were assigned if available information shows that a criterion of the risk of bias tool had or had not been met, and "unclear" was assigned if information was insufficient for objective assessment. The following agreements were made based on a (further) objectification of the 13 criteria:

- **Dissimilarity at baseline** was assessed by the results of statistical tests based on the following variables; primary outcome, severity/duration of somatic symptoms, age, gender, and employment/education.
- Low risk was scored if **attrition** rates were under 5% (post) or 10% (follow-up). Plausible standardized mean differences for missing outcome observations could not be established.
- For interpretation of the "acceptability" of **compliance**, it was agreed (a priori) to consider program duration, proportions of allocated participants that completed (at least 80%) the intervention for each group, and if compliance was monitored such that inadequate use could be observed. Objective assessment was complicated by differences in program duration and how usage/compliance was reported. After discussion, reasons for assigning high instead of low risk were: important differences in compliance between groups, a large number (more than half) of the intervention group participants stopped before completing (at least 80%) of a 3-12 week CBI. "Unclear" is assessed when compliance could not be judged by comparable standards (a CBI of longer duration was completed by less than half of the participants, if there was no monitoring, or usage was indicated in a completely different way).
- "Preregistered" as in the criterion for risk of reporting bias due to **selective outcome reporting** was interpreted as; registered before the end of data collections (note that some studies registered or updated a protocol between the start and end of data collection).
- Risk of bias due to **incomplete reporting and analysis** according to group allocation was assessed for:
    - Primary analysis performed: "low risk" means intention-to-treat analysis were presented (or sensitivity analysis showed that primary complete case analyses results did not differ);
    - data available for extraction (low risk is assigned if means and standard deviations are based on all participants that were allocated to the experimental groups (e.g. after adequate imputation of missing data)
- High risk due to different co-interventions between groups is assigned if intervention group participants were offered general information or training for using technology (not specific to the program under investigation). Other possible co-interventions, e.g. medication, were not considered.
- Other bias was coded high for studies with a small sample size (n<50).

https://back.cochrane.org/sites/back.cochrane.org/files/public/uploads/PDF/ROB%20criteria_Aug2011.pdf

**First author, year of publication**	Random sequence generation	Allocation concealment	Group similarity at baseline	**Selection bias**	**Attrition bias**	**Reporting bias** (selective outcome reporting)	Blinding of participants	Blinding of personnel/care providers	Similarity of co-interventions across groups	Compliance with interventions across groups	**Performance bias**	Bias due to incomplete analysis according to group allocation	Bias due to **incomplete reporting** according to group allocation	Blinding of outcome assessor	Timing of outcome assessment	**Detection bias**	**Other bias** (n<50)
Abbott, 2009																	
Andersson, 2002																	
Andersson, 2003																	
Boer, de, 2014																	
Brattberg, 2006			*1														

Buhrman, 2004																	
Buhrman, 2011																	
Buhrman, 2013																	
Buhrman, 2013b																	
Buhrman, 2015																	
Camerini, 2012																	
Carpenter, 2012																	
Chiauzzi, 2010																	
Davis, 2013																	
Dear, 2013																	
Dear, 2015																	
Devenini, 2005																	
Dowd, 2015																	
Everitt, 2013																	
Hesser, 2012			*2														
Hunt, 2009																	
Hunt, 2015																	
Janse, 2016																	
Jasper, 2014																	
Kaldo, 2008																	
Krein, 2013																	
Kristjánsdóttir, 2013																	
Lee, 2014																	
Ljotsson, 2010																	
Ljotsson, 2011a																	
Ljotsson, 2011b																	
Lorig, 2008																	
Menga, 2014																	
Moessner, 2014																	
Mourad, 2016																	
Naylor, 2008																	
Oerlemans, 2011																	
Riva, 2014																	
Ruehlman, 2012																	
Schulz, 2007																	
Strom, 2000																	
Trompetter, 2015																	
Vallejo, 2015			*3														
Weise, 2016																	
Williams, 2010																	
Wilson, 2015																	

*[1,2,3] High/unclear risk only for particular outcomes: 1; Health-related quality of life, Functional interference (FI), 2; FI, 3; FI, depression.

Table C: Outcome measures extracted by studies for each outcome category

Study, year of publication	Symptom intensity	Health-related quality of life	Functional interference	Catastrophizing	Depression
Abbott 2009	VAS Loudness	WHOQOL	TRQ		DASS
Andersson 2002	VAS Loudness		TRQ		HADS
Andersson 2003	Headache intensity diary 4x 0-5		HDI		HADS
Brattberg 2006	SF-36 bodily pain	SF-36 general health	SF-36 Role-physical		HADS
Buhrman 2004	MPI pain severity		MPI interference	CSQ cat.	HADS
Buhrman 2011	MPI pain severity	QOLI	MPI interference	CSQ cat.	HADS
Buhrman 2013	MPI pain severity	QOLI	MPI interference	CSQ cat.	HADS
Buhrman 2013a	MPI pain severity	QOLI	MPI interference	CSQ cat.	HADS
Buhrman 2015	MPI pain severity	QOLI	MPI interference	PCS	MADRS-S
Camerini 2012					
Carpenter 2012	VAS pain intensity		RMDQ	PCS rumination	
Chiauzzi 2010	BPI current		ODQ	PCS	DASS
de Boer 2014	VAS pain	RAND-36 General Health	VAS interference	PCS	
Dear 2013	WBPQ average pain		RMDQ	PRSS cat.	PHQ-9
Dear 2015	WBPQ average pain		RMDQ	CPAQ	PHQ-9
Deveneni 2005	Headache Index		HDI		CES-D
Dowd 2015	BPI pain right now		BPI interference	PCS	HADS (total)
Everitt 2013	IBS SSS	IBS QOL			HADS
Hesser 2012		QOLI	THI	TAQ	HADS
Hunt 2009	GSRS	IBS QOL		ASI–GI	
Hunt 2015	GSRS	IBS QOL		VSI	
Janse 2016	CIS fatigue severity		SIP 8		SCL-90 (total)
Jasper 2014			THI	TAQ	HADS
Kaldo 2008	VAS loudness		THI		HADS
Knoop 2008	CIS fatigue severity		SIP 8		
Krein 2013	VAS pain		RMDQ	TSK	
Kristjánsdóttir 2013	VAS Pain		FIQ	CPAQ	GHQ emotional distress
Lee 2014	Pain VAS	SF36 general health	SF36 role physical		
Ljotsson 2011	GSRS-IBS	IBS QOL	Sheehan Disability Scales	VSI	
Ljotsson 2011a	GSRS-IBS	IBS QOL		VSI	
Ljótsson 2010	GSRS-IBS	IBS QOL	Sheehan disability scales	VSI	MADRS-S
Lorig 2008	Pain NRS	Self-reported global health	Disability (National Health Survey)	ASES	
Menga 2014			FIQ		
Moessner 2014	Pain NRS	SF-36	RMDQ		
Mourad 2016					PHQ-9
Naylor 2008	MPQ pain now		TOPS Total Pain Experience	CSQ	
Oerlemans 2011	Abdominal pain (0-5)			PCS	
Riva 2014	CPGS				
Ruehlman 2012	PCP-S severity		PCP-S interference		
Schulz 2007					
Ström 2000	Headache diary peak intensity		HDI		

Trompetter 2015	Pain NRS		MPI interference	PCS	HADS
Vallejo 2015			FIQ	PCS	BDI
Weise 2016			THI	TAQ	HADS
Williams 2010	BPI		SF-36 physical functioning		CES-D
Wilson 2015	BPI pain intensity		BPI interference	PSEQ	PHQ8

VAS; Visual Analogue Scale, SF; Short-form Health Survey, MPI; Multidimensional Pain Inventory, BPI; Brief Pain Inventory, WBPQ; Wisconsin Brief Pain Questionnaire, IBS-SSS; Irritable Bowel Syndrome Symptom Severity Score, GSRS; Gastrointestinal Symptom Rating Scale, CIS; Checklist Individual Strength, MPQ; McGill Pain Questionnaire, CPGS: Chronic Pain Grading Scale, PCP-S; Profile of Chronic Pain – Screen, NRS; Numerical Rating Scale, WHOQOL; World Health Organization quality of life assessment, QOLI; Quality of Life Inventory, IBS QOL; Irritable Bowel Syndrome Quality of Life Instrument, TRQ; Tinnitus Reaction Questionnaire, HDI; Headache Disability Index, RMDQ; Roland-Morris Disability Questionnaire, THI; Tinnitus Handicap Inventory, SIP; Sickness Impact Profile, FIQ; Fibromyalgia Impact Questionnaire, TOPS; Treatment Outcomes of Pain Survey, CSQ cat.; Coping Strategies Questionnaire catastrophizing subscale, PCS; Pain Catastrophizing Scale, PRSS cat; Pain Related Control Scales catastrophizing subscale, TAQ; Tinnitus Acceptance Questionnaire, TSK; Tampa Scale of Kinesiophobia, ASI; Anxiety Sensitivity Index, VSI; Visceral Sensitivity Index, CPAQ; Chronic Pain Acceptance Questionnaire, ASES; Arthritis Self-Efficacy Scale, PSES; Pain Self-Efficacy Scale, DASS; Depression Anxiety Stress Scales, HADS; Hospital Anxiety and Depression Scale, MADRS-S; Montgomery–Åsberg Depression Rating Scale, PHQ; Patient Health Questionnaire, CES-D; Center for Epidemiological Studies Depression Scale, SCL; Symptoms Checklist, GHQ; GHQ; General Health Questionnaire, BDI; Beck Depression Inventory

Table D: Coding results for use of theory (1), individual BCTs (2), and modes of delivery (3)

1. *Use of theory*

A First author name	A Year	1	2	3	4	5	6	7	8	9	1	11	theory	Theory explicit link construct and intervention
Abbott	2009	1								1			1	1
Andersson	2002	1				1			1				1	1
Andersson	2003												0	0
Brattberg	2006												0	0
Buhrman	2004	1				1							1	0
Buhrman	2011	1											6	0
Buhrman	2015	1	1			1	1						6	0
Buhrman(a)	2013	1				1			1			1	3	1
Buhrman(b)	2013	1	1			1							6	0
Camerini	2012	1							1				4	1
Carpenter	2012	1				1							6	0
Chiauzzi	2010	1											1	0
Davis	2013	1											3	0
de Boer	2014	1	1			1				1			1	1
Dear	2015	1											2	0
Dear	2013	1											1	0
Devenini	2005												0	0
Dowd	2015	1											6	0
Everitt	2013	1											1	0
Hesser	2012	1											3	0
Hunt	2015	1	1			1			1				1	1
Hunt	2009	1	1			1			1				1	1
Janse	2016	1	1			1			1				1	1
Jasper	2014	1											1	0
Kaldo	2008	1								1			1	1
Krein	2013	1				1				1			5	1
Kristjánsdóttir	2013	1	1			1			1				3	1
Lee	2014												0	0
Ljotsson	2011	1	1			1			1				6	1
Ljotsson	2010	1	1			1			1				3	1
Ljotsson	2011	1	1			1			1				3	1
Lorig	2008	1	1										4	0
Menga	2014	1											2	0
Moessner	2014												0	0
Mourad	2016	1											1	0
Naylor	2008	1				1				1			2	1
Oerlemans	2011	1				1			1				1	1
Riva	2014	1											4	0
Ruehlman	2012	1				1							5	0
Schulz	2007												0	0
Strom	2000												0	0

Trompetter	2015	1			1	1		1			1		3	1
Vallejo	2015	1											2	0
Weise	2016	1								1			1	1
Williams	2010	1				1		1					1	1
Wilson	2015	1				1							5	0

Column explanations:

- 1. Theory/model of behavior mentioned
- 2. Targeted construct mentioned as predictor of behavior
- 3. Intervention based on single theory
- 4. Use of theory predictors to select recipients for the intervention
- 5. Use of theory predictors to select/develop intervention techniques.
- 6. Use of theory predictors to tailor intervention techniques to recipients.
- 7. All intervention techniques are linked to theory
- 8. At least one of the intervention techniques is linked to theory
- 9. Group of techniques are linked to a group of constructs/predictors
- 10. All theory-relevant constructs are linked to intervention techniques
- 11. At least one of the theory-relevant constructs is linked to an intervention technique
- Categories of “theory”: 0 = none, 1 = simply CBT, 2 = CBT combination with other, 3 = third wave, 4 = other, 5 = author constructed, 6 = third wave inspired CBI combination.
- Explicit links between constructs and intervention = item 7 OR 8 OR 9

2. *Behavioral change techniques (BCTs)*

Full detail on the Behavioral Change Technique Taxonomy version 1 (BCTTv1) is available to users via: http://www.bct-taxonomy.com/. Considerations during application of the BCTTv1:

- BCTs specified in earlier protocols or study reports from the first author are assumed to be present (unless the later study conveys that it is not).
- The provision of information about antecedents (4.2) and information about health consequences (5.1) were automatically coded if participants received an explanation of the treatment rationale within a CBT.
- Interventions based on Acceptance and Commitment Therapy (ACT) included “cognitive defusion” and “values”. If these were mentioned, the BCTs “framing/reframing” (13.2) and “valued self-identity” (13.4) were coded (subsequently).

Study	Comparison	1.1	1.2	1.3	1.4	1.5	1.6	1.7	2.2	2.3	2.4	2.7	3.1	4.1	4.2	4.4	5.1	5.6	6.1	6.2	7.7	8.1	8.2	8.3	8.4	8.6	8.7	9.1	10.3	11.2	12.1	12.3	12.4	12.6	13.2	13.4	15.4
Abbott09	p			1					1				1	1	1		1		1		1	1					1	1		1	1		1	1	1		
Andersson02	p		1										1	1	1		1		1			1						1		1	1			1	1		
Andersson03	a		0							0				0		0	0		0			0						0		0				0			
de Boer14	a												0	0	0		0		0			0					0	0		0			0	0	0		
Brattberg06	p												1							1								1									
Buhrman04	p		1	1						1			1	1	1		1		1			1					1	1		1				1	1		
Buhrman11	p			1	1					1			1	1	1		1		1			1					1	1		1				1	1		
Buhrman13a	p		1	1						1	1		1	1	1		1		1			1						1		1				1	1	1	
Buhrman13b	p		1	1	1					1			1	1	1		1		1			1						1		1		1		1	1		
Buhrman15	p		1	1	1					1			1	1	1	1	1		1		1	1						1		1		1		1	1	1	
Camerini12	a												0	1			1		1									1									
Carpenter12	p			1	1								1	1	1		1	1	1			1								1				1	1	1	
Chiauzzi10	p		1	1										1														1		1							
Davis13	p													1	1		1		1			1								1					1		
Dear13	p		1	1						1	1		1	1	1		1		1	1	1	1					1			1				1	1		
Dear15	p		1	1						1	1		1	1	1		1		1	1	1	1					1	1		1				1	1		
Dear15	a		0	0									1	0	0		0		0	0	0	0					0	1						0	0		
Devenini05	p									1				1	1		1		1			1				1				1				1	1		
Dowd15	p													1					1			1								1							
Everitt13	p	1	1		1	1				1	1		1	1	1		1		1			1						1		1				1	1		
Everitt13	a	0	0		0	0				0	0		1	0	0		0		0			0						1		0				0	0		
Hesser12	p			1					1				1	1	1		1		1		1	1						1		1	1		1	1	1	1	
Hesser12	a			0					0				0	0	0		0		0		0	0						0		0	0		0	0	0	1	
Hunt15	p								1	1				1	1	1	1		1		1	1		1						1				1	1		
Hunt09	p								1	1			1	1	1	1	1		1		1	1						1		1				1	1		
Janse16	p	1	1	1						1			1	1	1		1										1	1					1		1		
Jasper14	p												1	1	1		1		1			1		1				1		1	1		1	1	1		
Jasper14	a		-1											0					0		1	0		1						0	1		1	0	0		
Kaldo08	a		0	0				0	0				0	0	0		0		0		0	0						0		0	0		0	0	0		
Krein13	p	1				1	1		1				1	1					1	1		1						1									
Kristjánsdóttir13	a				1		1		1	1			1	1	1							1						1	1	1					1	1	
Lee14	p									1				1			1																				

Ljotsson10	p		1										1	1	1		1		1		1	1	1					1		1					1		
Ljotsson11a	a														1						1		1												1		
Ljotsson11b	p		1						1	1			1	1	1		1		1		1	1	1					1		1					1		
Lorig08	p		1	1	1					1			1	1					1			1								1			1	1			1
Menga14	p		1											1	1		1		1			1												1	1		
Moessner14	a		1							1													1					1									
Mourad16	p			1						1	1	1	1	1	1	1	1		1		1	1					1	1		1				1			
Naylor08	a									1														1				1									
Oerlemans11	p		1		1				1	1	1				1		1	1						1	1			1		1					1		
Riva14	a												1	1					1	1																	
Ruehlman12	p								1	1	1	1		1	1		1		1			1		1			1			1				1	1		
Schulz07	p												1	1					1	1								1									
Strom00	p		1											1		1			1			1								1				1			
Trompetter15	p												1	1	1		1		1	1		1		1						1					1	1	
Trompetter15	a												0	1	1		0		1	1		1		1						1					1	1	
Vallejo15	p												1	1	1		1		1			1		1				1		1				1	1		
Vallejo15	a												0	0	0		0		0			0		0				0		0				0	0		
Weise16	p		1	1				1	1				1	1	1		1		1		1	1						1		1	1		1	1	1		
Williams10	p		1	1						1			1	1	1		1		1			1					1	1		1				1	1		
Wilson15	p								1	1	1	1		1	1		1		1			1		1			1			1				1	1		

Cell explanations: 1 = BCT is present in experimental versus control group, 0 = BCT is present in experimental and control group, -1 = BCT is present in control group and not in experimental group.

Column explanations:

- Comparison A = active, p = passive
- Remaining columns are the techniques of the total 93 that were coded. They are categorized 1-16:
  - 1. Goals and planning: 1.1 Goal setting (behavior), 1.2 Problem solving, 1.3 Goal setting (outcome), 1.4 Action planning, 1.5 Review behavior goal(s), 1.6 Discrepancy between current behavior and goal, 1.7 Review outcome goal(s), 1.8 Behavioral contract, 1.9 Commitment
  - 2. Feedback and monitoring: 2.1 Monitoring of behavior by other without feedback, 2.2 Feedback on behavior, 2.3 Self-monitoring of behavior, 2.4 Self-monitoring of outcome(s) of behavior, 2.5 Monitoring of outcome(s) of behavior without , 2.6 Biofeedback, 2.7 Feedback on outcome(s) of behavior
  - 3. Social support: 3.1 Social support (unspecified), 3.2 Social support (practical), 3.3 Social support (emotional)
  - 4. Shaping knowledge: 4.1 Instruction on how to perform the behavior, 4.2 Information about antecedents, 4.3 Re-attribution, 4.4 Behavioral experiments
  - 5. Natural consequences: 5.1 Information about health consequences, 5.2 Salience of consequences, 5.3 Information about social and environment consequences, 5.4 Monitoring of emotional consequences, 5.5 Anticipated regret, 5.6 Information about emotional consequences
  - 6. Comparison of behavior: 6.1 Demonstration of the behavior, 6.2 Social comparison, 6.3 Information about others" approval
  - 7. Associations: 7.1 Prompts/cues, 7.2 Cue signaling reward, 7.3 Reduce prompts/cues, 7.4 Remove access to the reward, 7.5 Remove aversive stimulus, 7.6 Satiation, 7.7 Exposure, 7.8 Associative learning
  - 8. Repetition and substitution: 8.1 Behavioral practice/rehearsal, 8.2 Behavior substitution, 8.3 Habit formation, 8.4 Habit reversal, 8.5 Overcorrection, 8.6 Generalization of target behavior, 8.7 Graded tasks
  - 9. Comparison of outcomes: 9.1 Credible source, 9.2 Pros and cons, 9.3 Comparative imagining of future outcomes
  - 10. Reward and threat: 10.1 Material incentive (behavior), 10.2 Material reward (behavior), 10.3 Non-specific reward, 10.4 Social reward, 10.5 Social incentive, 10.6 Non-specific incentive, 10.7 Self-incentive, 10.8 Incentive (outcome), 10.9 Self-reward, 10.10 Reward (outcome), 10.11 Future punishment
  - 11. Regulation: 11.1 Pharmacological support, 11.2 Reduce negative emotions, 11.3 Conserving mental resources, 11.4 Paradoxical instructions
  - 12. Antecedents: 12.1 Restructuring the physical environment, 12.2 Restructuring the social environment, 12.3 Avoidance/reducing exposure to cues for the behavior, 12.4 Distraction, 12.5 Adding object to the environment, 12.6 Body changes
  - 13. Identity: 13.1 Identification of self as role model, 13.2 Framing/reframing, 13.3 Incompatible beliefs, 13.4 Valued self-identity, 13.5 Identity associated with changed behavior
  - 14. Scheduled consequences: 14.1 Behavior cost, 14.2 Punishment, 14.3 Remove reward, 14.4 Reward approximation, 14.5 Rewarding completion, 14.6 Situation-specific reward, 14.7 Reward incompatible behavior, 14.8 Reward alternative behavior, 14.9 Reduce reward frequency, 14.10 Remove punishment
  - 15. Self-belief: 15.1 Verbal persuasion about capability, 15.2 Mental rehearsal of successful performance, 15.3 Focus on past success, 15.4 Self-talk
  - 16. Covert learning: 16.1 Imaginary punishment, 16.2 Imaginary reward, 16.3 Vicarious consequences

3. *Modes of delivery*

First author	Year of publication	Comparison	1	2	3	4	5	6	7	8	9	1	11
Abbott	2009	Passive	1		1		1		1			1	
Andersson	2002	Passive	1				1		1			1	
Brattberg	2006	Passive		1		1		1	1				1
Buhrman	2004	Passive	1	1	1		1		1		1	1	1
Buhrman	2011	Passive	1		1		1		1			1	
Buhrman	2015	Passive	1		1		1		1		1	1	
Buhrman(a)	2013	Passive	1	1	1		1		1		1	1	
Buhrman(b)	2013	Passive	1				1		1			1	
Carpenter	2012	Passive	1	1	1				1			1	
Chiauzzi	2010	Passive	1	1	1				1			1	
Davis	2013	Passive	1	1	1				1			1	
Dear	2015	Passive	1		1		1		1		1	1	
Dear	2013	Passive	1		1		1		1		1	1	
Devenini	2005	Passive	1	1					1			1	
Dowd	2015	Passive		1	1				1			1	
Everitt	2013	Passive	1	1			1		1			1	
Hesser	2012	Passive	1	1	1		1		1		1		
Hunt	2015	Passive			1				1			1	
Hunt	2009	Passive	1		1		1		1			1	
Janse	2016	Passive	1				1					1	
Jasper	2014	Passive					1		1			1	
Krein	2013	Passive	1	1	1			1	1			1	
Lee	2014	Passive	1		1				1	1			
Ljotsson	2011	Passive	1		1		1	1	1			1	
Ljotsson	2010	Passive	1		1		1	1	1			1	
Lorig	2008	Passive	1	1	1		1	1	1				
Menga	2014	Passive		1					1				
Mourad	2016	Passive	1	1			1		1	1	1	1	
Oerlemans	2011	Passive	1		1					1			
Ruehlman	2012	Passive	1	1	1			1	1	1		1	
Schulz	2007	Passive		1	1	1		1	1				
Strom	2000	Passive	1		1				1			1	
Trompetter	2015	Passive	1	1			1		1			1	
Vallejo	2015	Passive	1	1	1		1		1				
Weise	2016	Passive	1	1	1		1		1			1	
Williams	2010	Passive		1	1				1		1	1	
Wilson	2015	Passive	1	1	1			1	1	1		1	
Andersson	2003	Active									1		
Camerini	2012	Active		1		1		1	1				
de Boer	2014	Active	1	1	1		1		1		1	1	
Dear	2015	Active					1				1		
Everitt	2013	Active					1					1	
Hesser	2012	Active		1									
Jasper	2014	Active					1		1			1	
Kaldo	2008	Active	1		1		1		1			1	
Kristjánsdóttir	2013	Active	1		1				1	1	1		
Ljotsson	2011	Active											
Moessner	2014	Active	1				1	1	1				
Naylor	2008	Active	1								1		
Riva	2014	Active		1									
Trompetter	2015	Active		1			1						
Vallejo	2015	Active	1	1	1		1		1				

Column explanations: 1-11 are the items of the taxonomy. Here mentioned per category:

- Automated Functions: 1. Automated tailored feedback, 2. Enriched information environment, 3. Automated follow-up messages
- Communicative Functions: 4. Access to advisor to request advice, 5. Scheduled contact with advisor, 6. Peer-to-peer access
- Supplementary modes: 7. Internet, 8. Text message (SMS), 9. Telephone, 10. Email, 11. CD-ROM

Overview of meta-analyses; *SMD*s, 95% confidence intervals, heterogeneity statistics, forest plots and funnel plots), and sensitivity analyses

Comparison 1: Computer-based interventions versus passive controls (tables F-O, figs A-N)

Outcome 1.1: Symptom intensity (post)
Number of eligible studies reporting the outcome: 29
Total number participants: 3284

Table F

	SMD	**95% CI**	**I^2**	***P***
All eligible studies (k = 29)	-.35	[-.48, -.22]	65%	<.001
SENSITIVITY ANALYSES				
**Internal validity**				
Low risk selection bias (k = 4)	-.44	[-.69, -.20]	40%	.17
Low risk attrition bias (k = 5)*	-.57	[-.93, -.22]	71%	.009
Low risk reporting bias (k = 6)	-.31	[-.61, -.01]	80%	<.001
Low risk performance bias (k = 9)	-.45	[-.65, -.24]	50%	.04
Low risk due to incomplete data extracted (k = 8)	-.39	[-.56, -.23]	28%	.20
Low risk detection bias (k = 27)	-.34	[-.47, -.21]	65%	<.001
Low risk other bias (k = 25)	-.33	[-.46, -.20]	66%	<.001
**External validity**				
Participants recruited from a general (open) population (k = 17)	-.35	[-.50, -.21]	53%	.006
Participants recruited from a clinical (closed) population (k = 7)	-.33	[-.68, .02]	79%	<.001

SMD = Standardized mean difference, *CI* = Confidence interval, *P* = P-value for a Chi-square test for Tau; a measure of heterogeneity of standardized mean differences
* Test for subgroup differences (attrition bias): $Chi^2 = 1.96$, df = 1 (P = 0.16), $I^2 = 49.0\%$

Figure A: Forest plot all studies

Study or Subgroup	Experimental Mean	Experimental SD	Experimental Total	Control Mean	Control SD	Control Total	Weight	Std. Mean Difference IV, Random, 95% CI
Hunt 2009	35	12	13	52	14	18	1.8%	-1.25 [-2.04, -0.47]
Ljótsson 2010	32.2	12.1	34	47.3	12.6	43	3.2%	-1.21 [-1.70, -0.72]
Hunt 2015	42	13.5	17	55	11.5	19	2.1%	-1.02 [-1.72, -0.32]
Lee 2014	3.03	1.9	33	5.13	2.42	32	3.0%	-0.96 [-1.47, -0.44]
Devineni 2005	18.6	13	39	30.6	14.7	47	3.5%	-0.85 [-1.30, -0.41]
Ljotsson 2011	31	10.2	23	40.9	14.5	27	2.7%	-0.77 [-1.34, -0.19]
Knoop 2008	38.9	12.1	85	46.4	8.7	86	0.0%	-0.71 [-1.02, -0.40]
Janse 2016	32.77	14.82	50	41.6	11.09	50	3.7%	-0.67 [-1.07, -0.27]
Dowd 2015	3.73	2.12	23	5.1	2.07	27	2.7%	-0.64 [-1.22, -0.07]
Dear 2013	4.68	1.7	30	5.81	1.85	30	3.0%	-0.63 [-1.15, -0.11]
Dear 2015	4.68	1.79	139	5.71	1.5	74	4.6%	-0.61 [-0.89, -0.32]
Buhrman 2013	3.72	1.1	36	4.18	1.21	36	3.3%	-0.39 [-0.86, 0.07]
Williams 2010	4.3	1.6	59	4.9	1.5	59	4.0%	-0.38 [-0.75, -0.02]
Oerlemans 2011	1.46	1.3	36	1.89	1.3	36	3.3%	-0.33 [-0.79, 0.14]
Buhrman 2004	34.3	16.8	22	39.6	16.3	29	2.8%	-0.32 [-0.87, 0.24]
Carpenter 2012	5.2	1.5	63	5.7	1.7	68	4.2%	-0.31 [-0.65, 0.04]
Ström 2000	49.78	26.38	20	56.26	19.54	25	2.6%	-0.28 [-0.87, 0.31]
Brattberg 2006	-50	30.1	27	-42.1	27.1	28	2.9%	-0.27 [-0.80, 0.26]
Krein 2013	4.7	2.1	101	5.2	2.1	106	4.7%	-0.24 [-0.51, 0.04]
Chiauzzi 2010	4.64	2.53	95	5.17	2.35	104	4.6%	-0.22 [-0.50, 0.06]
Lorig 2008	5.86	2.44	310	6.34	2.31	331	5.5%	-0.20 [-0.36, -0.05]
Buhrman 2015	3.75	1.05	28	3.95	0.93	24	2.9%	-0.20 [-0.74, 0.35]
Trompetter 2015	5.4	2.2	59	5.6	2.1	62	4.1%	-0.09 [-0.45, 0.26]
Andersson 2002	6.2	2.3	24	6.4	2.1	59	3.3%	-0.09 [-0.57, 0.38]
Buhrman 2011	3.15	2.2	23	3.35	2.6	27	2.8%	-0.08 [-0.64, 0.48]
Ruehlman 2012	22.75	4.14	162	22.93	4.25	143	5.0%	-0.04 [-0.27, 0.18]
Vallejo 2015	0	0	0	0	0	0		Not estimable
Weise 2016	0	0	0	0	0	0		Not estimable
Moessner 2014	0	0	0	0	0	0		Not estimable
Mourad 2016	0	0	0	0	0	0		Not estimable
Naylor 2008	0	0	0	0	0	0		Not estimable
Riva 2014	0	0	0	0	0	0		Not estimable
Schulz 2007	0	0	0	0	0	0		Not estimable
Kristjánsdóttir 2013	0	0	0	0	0	0		Not estimable
Ljotsson 2011a	0	0	0	0	0	0		Not estimable
Menga 2014	0	0	0	0	0	0		Not estimable
Kaldo 2008	0	0	0	0	0	0		Not estimable
Hesser 2012	0	0	0	0	0	0		Not estimable
Jasper 2014	0	0	0	0	0	0		Not estimable
Davis 2013	0	0	0	0	0	0		Not estimable
de Boer 2014	0	0	0	0	0	0		Not estimable
Andersson 2003	0	0	0	0	0	0		Not estimable
Camerini 2012	0	0	0	0	0	0		Not estimable
Buhrman 2013a	4.3	1.04	38	4.29	1	38	3.4%	0.01 [-0.44, 0.46]
Abbott 2009	4.58	1.7	28	4.48	1.7	28	3.0%	0.06 [-0.47, 0.58]
Wilson 2015	5.3	1.9	45	5.1	1.8	47	3.7%	0.11 [-0.30, 0.52]
Everitt 2013	208	83.2132	45	162.8	84.5446	45	3.6%	0.53 [0.11, 0.96]
**Total (95% CI)**			**1622**			**1662**	**100.0%**	**-0.35 [-0.48, -0.22]**

Heterogeneity: Tau² = 0.07; Chi² = 80.67, df = 28 (P < 0.00001); I² = 65%
Test for overall effect: Z = 5.37 (P < 0.00001)

Std. Mean Difference
IV, Random, 95% CI

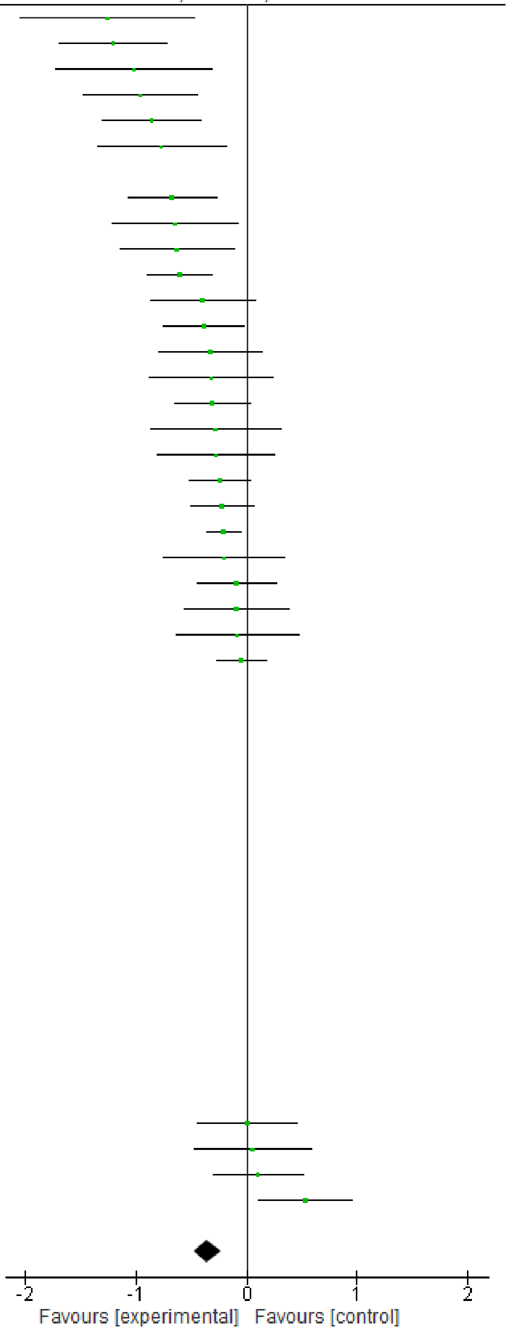

Figure B: Funnel plot all studies

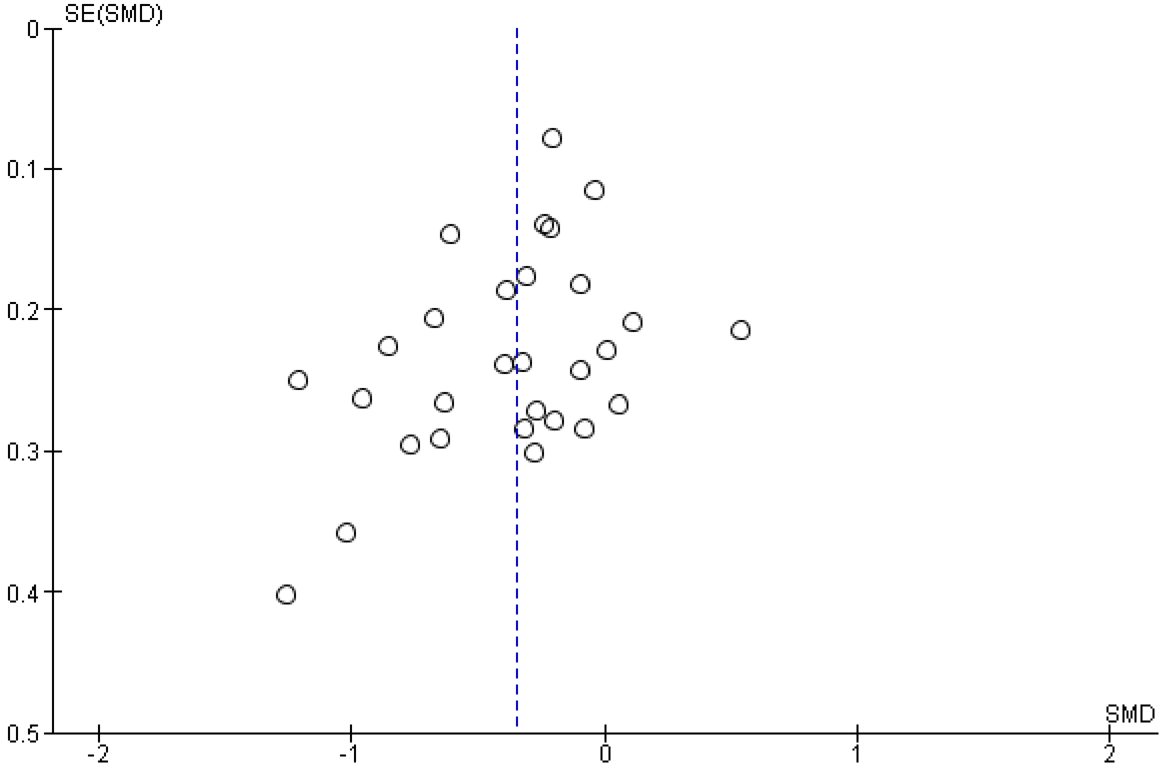

Outcome 1.2: Health-related Quality Of Life (post)
Number of eligible studies reporting the outcome: 14
Total number participants: 1408

Table G

	SMD	**95% CI**	I^2	***P***
All eligible studies (k = 14)	-.36	[-.58, -.13]	70%	<.001
SENSITIVITY ANALYSES				
**Internal validity**				
Low risk selection bias (k = 3)	-.50	[-.86, -.13]	27%	.25
Low risk attrition bias (k = 3)	-.38	[-.97, .21]	75%	.02
Low risk reporting bias (k = 1)	.14	[-.27, .56]	/	/
Low risk performance bias (k = 3)	-.51	[-.99, -.03]	62%	.07
Low risk due to incomplete data extracted (k = 3)	-.31	[-.59, -.03]	0%	.38
Low risk detection bias				
Low risk other bias (k = 12)	-.25	[-.46, -.05]	62%	.002
**External validity**				
Participants recruited from a general (open) population (k = 8)*	-.49	[-.75, -.23]	61%	.01
Participants recruited from a clinical (closed) population (k = 4)*	.02	[-.45, .48]	70%	.02

SMD = Standardized mean difference, *CI* = Confidence interval, *P* = P-value for a Chi-square test for Tau; a measure of heterogeneity of standardized mean differences
* Test for subgroup differences: $Chi^2 = 2.75$, df = 1 (P = 0.10), $I^2 = 63.7\%$

Figure C: Forest plot all studies

	Experimental			Control				Std. Mean Difference
Study or Subgroup	Mean	SD	Total	Mean	SD	Total	Weight	IV, Random, 95% CI
Hunt 2015	-66	16	17	-36	23	19	5.0%	-1.47 [-2.21, -0.72]
Hunt 2009	84	26	13	111	25	18	4.9%	-1.03 [-1.80, -0.27]
Ljótsson 2010	-72.8	19.9	34	-52.9	21.3	43	7.5%	-0.95 [-1.43, -0.48]
Ljotsson 2011	-82.6	13.4	23	-67.4	23.1	27	6.5%	-0.78 [-1.35, -0.20]
Brattberg 2006	-58.6	22.5	27	-45.9	18.2	28	6.8%	-0.61 [-1.15, -0.07]
Buhrman 2011	-1.7	1.4	23	-1.1	1.6	27	6.6%	-0.39 [-0.95, 0.17]
Buhrman 2013	-1.3	2.07	36	-0.61	1.65	36	7.6%	-0.36 [-0.83, 0.10]
Lorig 2008	0.515	0.456	310	0.598	0.483	331	10.8%	-0.18 [-0.33, -0.02]
Hesser 2012	-2.53	1.55	30	-2.27	1.5	32	7.3%	-0.17 [-0.67, 0.33]
Buhrman 2013a	-0.56	2.07	38	-0.39	1.77	38	7.8%	-0.09 [-0.54, 0.36]
Schulz 2007	0	0	0	0	0	0		Not estimable
Naylor 2008	0	0	0	0	0	0		Not estimable
Mourad 2016	0	0	0	0	0	0		Not estimable
Kristjánsdóttir 2013	0	0	0	0	0	0		Not estimable
Naylor 2010	0	0	0	0	0	0		Not estimable
Devineni 2005	0	0	0	0	0	0		Not estimable
Krein 2013	0	0	0	0	0	0		Not estimable
Moessner 2014	0	0	0	0	0	0		Not estimable
Dear 2015	0	0	0	0	0	0		Not estimable
Jasper 2014	0	0	0	0	0	0		Not estimable
Janse 2016	0	0	0	0	0	0		Not estimable
Ström 2000	0	0	0	0	0	0		Not estimable
Weise 2016	0	0	0	0	0	0		Not estimable
Vallejo 2015	0	0	0	0	0	0		Not estimable
Wilson 2015	0	0	0	0	0	0		Not estimable
Williams 2010	0	0	0	0	0	0		Not estimable
Trompetter 2015	0	0	0	0	0	0		Not estimable
Ruehlman 2012	0	0	0	0	0	0		Not estimable
Menga 2014	0	0	0	0	0	0		Not estimable
Buhrman 2004	0	0	0	0	0	0		Not estimable
Oerlemans 2011	0	0	0	0	0	0		Not estimable
Ljotsson 2011a	0	0	0	0	0	0		Not estimable
Dear 2013	0	0	0	0	0	0		Not estimable
Riva 2014	0	0	0	0	0	0		Not estimable
Dowd 2015	0	0	0	0	0	0		Not estimable
de Boer 2014	0	0	0	0	0	0		Not estimable
Camerini 2012	0	0	0	0	0	0		Not estimable
Carpenter 2012	0	0	0	0	0	0		Not estimable
Andersson 2003	0	0	0	0	0	0		Not estimable
Andersson 2002	0	0	0	0	0	0		Not estimable
Kaldo 2008	0	0	0	0	0	0		Not estimable
Chiauzzi 2010	0	0	0	0	0	0		Not estimable
Davis 2013	0	0	0	0	0	0		Not estimable
Buhrman 2015	-1.38	1.78	28	-1.39	1.59	24	6.8%	0.01 [-0.54, 0.55]
Everitt 2013	-67.6	12.3155	45	-69.4	12.3155	45	8.2%	0.14 [-0.27, 0.56]
Lee 2014	-52.48	23.28	33	-57.59	18.64	32	7.4%	0.24 [-0.25, 0.73]
Abbott 2009	-66.95	13.3	28	-72.37	13.7	23	6.7%	0.40 [-0.16, 0.95]
**Total (95% CI)**			**685**			**723**	**100.0%**	**-0.32 [-0.55, -0.10]**

Heterogeneity: $Tau^2 = 0.11$; $Chi^2 = 42.77$, df = 13 (P < 0.0001); $I^2 = 70\%$
Test for overall effect: Z = 2.83 (P = 0.005)

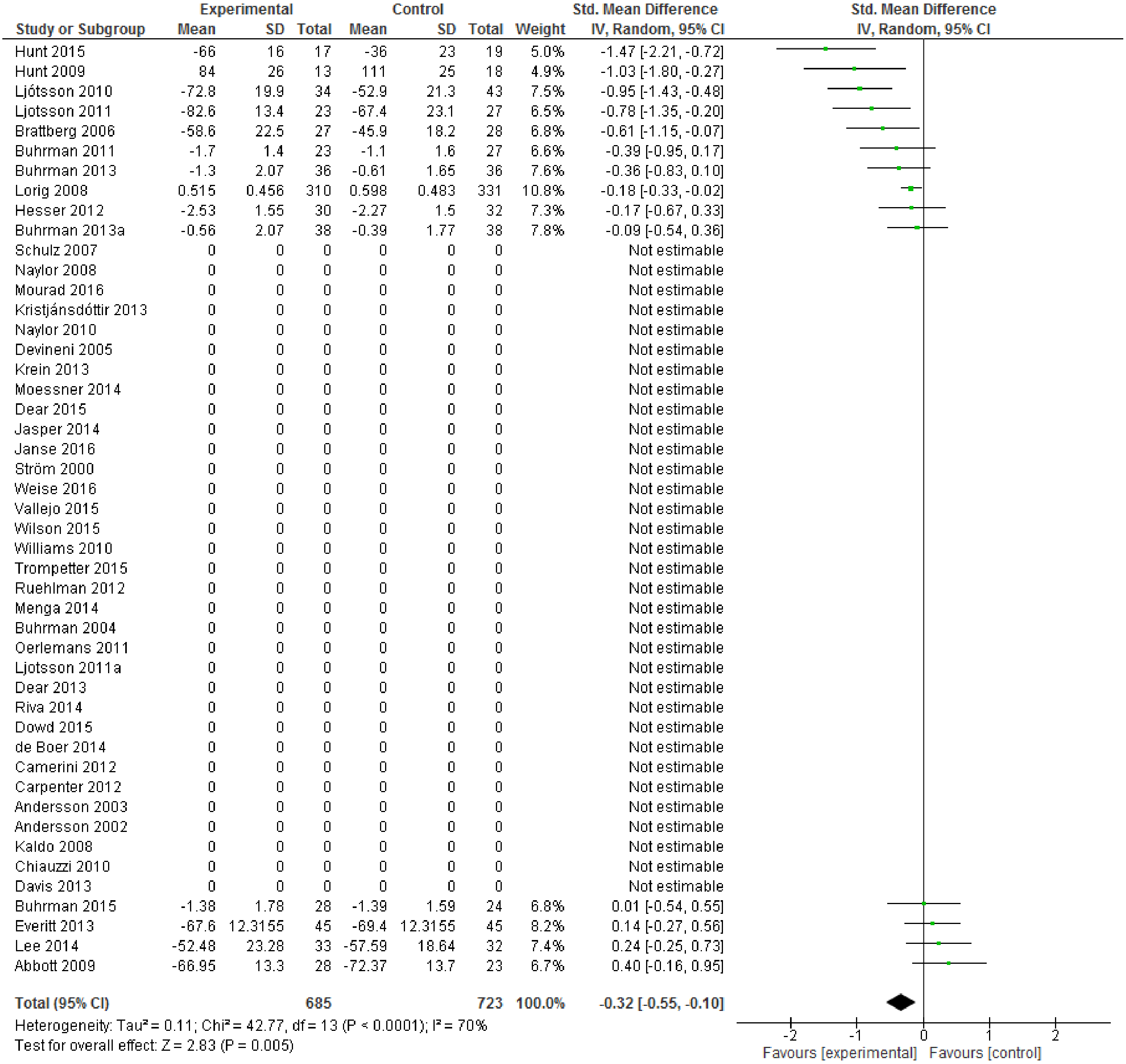

Figure D: Funnel plot all studies

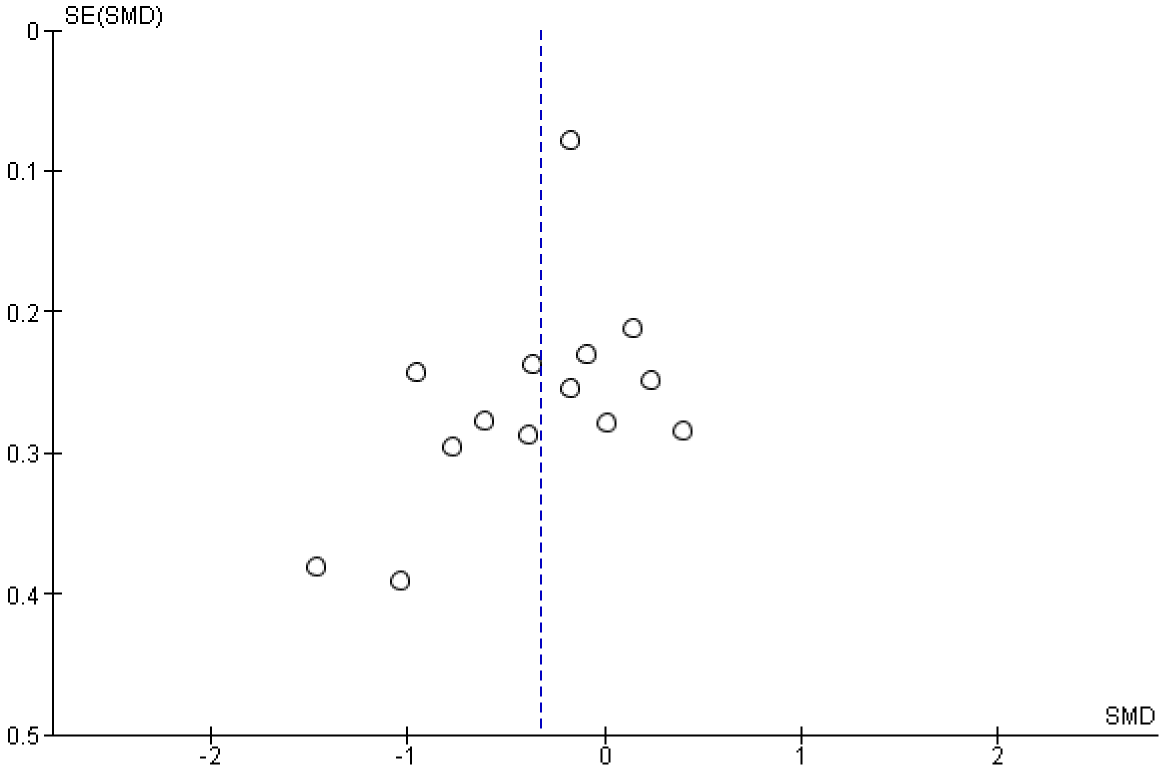

Outcome 1.3: Functional interference (post)

Number of eligible studies reporting the outcome: 30
Total number participants: 3387

Table H

	SMD	95% CI	I^2	*P*
All eligible studies (k = 30)	-.35	[-.45, -.25]	45%	.004
SENSITIVITY ANALYSES				
**Internal validity**				
Low risk selection bias (k = 4)	-.43	[-.61, -.25]	3%	.38
Low risk attrition bias (k = 8)*	-.53	[-.68, -.39]	0%	.44
Low risk reporting bias (k = 7)	-.50	[-.71, -.28]	66%	.007
Low risk performance bias (k = 12)	-.49	[-.61, -.36]	0%	.46
Low risk due to incomplete data extracted (k = 11)	-.48	[-.63, -.32]	40%	.08
Low risk detection bias				
Low risk other bias (k = 27)	-.35	[-.46, -.25]	48%	.004
**External validity**				
Participants recruited from a general (open) population (k = 18)	-.42	[-.56, -.28]	56%	.002
Participants recruited from a clinical (closed) population (k = 8)	-.28	[-.46, -.09]	26%	.22

SMD = Standardized mean difference, *CI* = Confidence interval, *P* = P-value for a Chi-square test for Tau; a measure of heterogeneity of standardized mean differences

* Test for subgroup differences: attrition bias $Chi^2 = 7.97$, df = 1 (P = 0.005), $I^2 = 87.5\%$, performance bias $Chi^2 = 5.10$, df = 1 (P = 0.02), $I^2 = 80.4\%$

Figure E: Forest plot

Study or Subgroup	Experimental Mean	Experimental SD	Experimental Total	Control Mean	Control SD	Control Total	Weight	Std. Mean Difference IV, Random, 95% CI
Menga 2014	43.9	16.4	17	58.6	16.1	27	1.9%	-0.89 [-1.53, -0.25]
Dear 2013	10.1	5.23	30	14.77	5.33	30	2.5%	-0.87 [-1.40, -0.34]
Weise 2016	32.56	16.5	62	45.77	15.06	62	4.0%	-0.83 [-1.20, -0.46]
Brattberg 2006	-35.2	37.5	27	-10.7	26.7	28	2.4%	-0.74 [-1.29, -0.20]
Buhrman 2013	3.62	1.08	36	4.32	1.12	36	2.9%	-0.63 [-1.10, -0.16]
Hesser 2012	38.93	19.72	33	49.94	16.09	32	2.7%	-0.60 [-1.10, -0.11]
Buhrman 2013a	4.37	1.09	38	4.94	0.93	38	3.1%	-0.56 [-1.02, -0.10]
Jasper 2014	26.67	20.75	41	37.46	18.94	44	3.3%	-0.54 [-0.97, -0.11]
Devineni 2005	38	19.5	39	49.6	23.1	47	3.3%	-0.53 [-0.97, -0.10]
Dear 2015	11.05	5.63	139	13.97	5.17	74	5.0%	-0.53 [-0.82, -0.24]
Janse 2016	458.36	576.88	50	731.58	455.34	50	3.6%	-0.52 [-0.92, -0.12]
Carpenter 2012	13.5	5.8	63	16.3	5.4	68	4.2%	-0.50 [-0.85, -0.15]
Ljótsson 2010	7.5	7.4	38	11.4	9	43	3.2%	-0.47 [-0.91, -0.02]
Dowd 2015	24.83	15.3	23	31.5	14.7	27	2.3%	-0.44 [-1.00, 0.12]
Buhrman 2015	3.31	1.29	28	3.8	1.21	24	2.4%	-0.38 [-0.94, 0.17]
Knoop 2008	1,079	690	85	1,319	619	86	0.0%	-0.36 [-0.67, -0.06]
Krein 2013	6.6	5	101	8.2	5	106	5.2%	-0.32 [-0.59, -0.04]
Trompetter 2015	28.7	12	59	32.1	12.3	62	4.1%	-0.28 [-0.64, 0.08]
Andersson 2002	29.5	22.2	24	35.4	23	48	2.8%	-0.26 [-0.75, 0.24]
Williams 2010	-41.1	8.7	59	-38.9	8.6	59	4.0%	-0.25 [-0.62, 0.11]
Buhrman 2004	3.2	1.4	22	3.5	1.2	29	2.4%	-0.23 [-0.79, 0.33]
Buhrman 2011	3.2	1.4	23	3.5	1.2	27	2.3%	-0.23 [-0.79, 0.33]
Ljotsson 2011	6.4	6.7	23	7.8	7.6	27	2.3%	-0.19 [-0.75, 0.37]
Lorig 2008	1.97	1.32	310	2.19	1.07	331	7.0%	-0.18 [-0.34, -0.03]
Vallejo 2015	56.99	18.17	20	60.35	17.72	20	2.0%	-0.18 [-0.80, 0.44]
Chiauzzi 2010	42.62	18.3	95	44.09	17.7	104	5.1%	-0.08 [-0.36, 0.20]
Wilson 2015	5.3	2.6	45	5.5	2.4	47	3.5%	-0.08 [-0.49, 0.33]
Lee 2014	-74.24	37.23	33	-72.66	30.69	32	2.8%	-0.05 [-0.53, 0.44]
de Boer 2014	0	0	0	0	0	0		Not estimable
Naylor 2008	0	0	0	0	0	0		Not estimable
Oerlemans 2011	0	0	0	0	0	0		Not estimable
Riva 2014	0	0	0	0	0	0		Not estimable
Moessner 2014	0	0	0	0	0	0		Not estimable
Davis 2013	0	0	0	0	0	0		Not estimable
Andersson 2003	0	0	0	0	0	0		Not estimable
Schulz 2007	0	0	0	0	0	0		Not estimable
Mourad 2016	0	0	0	0	0	0		Not estimable
Ljotsson 2011a	0	0	0	0	0	0		Not estimable
Kristjánsdóttir 2013	0	0	0	0	0	0		Not estimable
Camerini 2012	0	0	0	0	0	0		Not estimable
Everitt 2013	0	0	0	0	0	0		Not estimable
Kaldo 2008	0	0	0	0	0	0		Not estimable
Hunt 2015	0	0	0	0	0	0		Not estimable
Hunt 2009	0	0	0	0	0	0		Not estimable
Ruehlman 2012	22.31	8.61	162	21.85	8.47	143	5.9%	0.05 [-0.17, 0.28]
Ström 2000	40.55	15.57	11	36.4	22.07	20	1.5%	0.20 [-0.54, 0.94]
Abbott 2009	16.64	12.3	28	13.96	9.7	23	2.4%	0.24 [-0.32, 0.79]
**Total (95% CI)**			**1679**			**1708**	**100.0%**	**-0.35 [-0.45, -0.25]**

Heterogeneity: $Tau^2 = 0.03$; $Chi^2 = 52.95$, df = 29 ($P = 0.004$); $I^2 = 45\%$
Test for overall effect: $Z = 6.83$ ($P < 0.00001$)

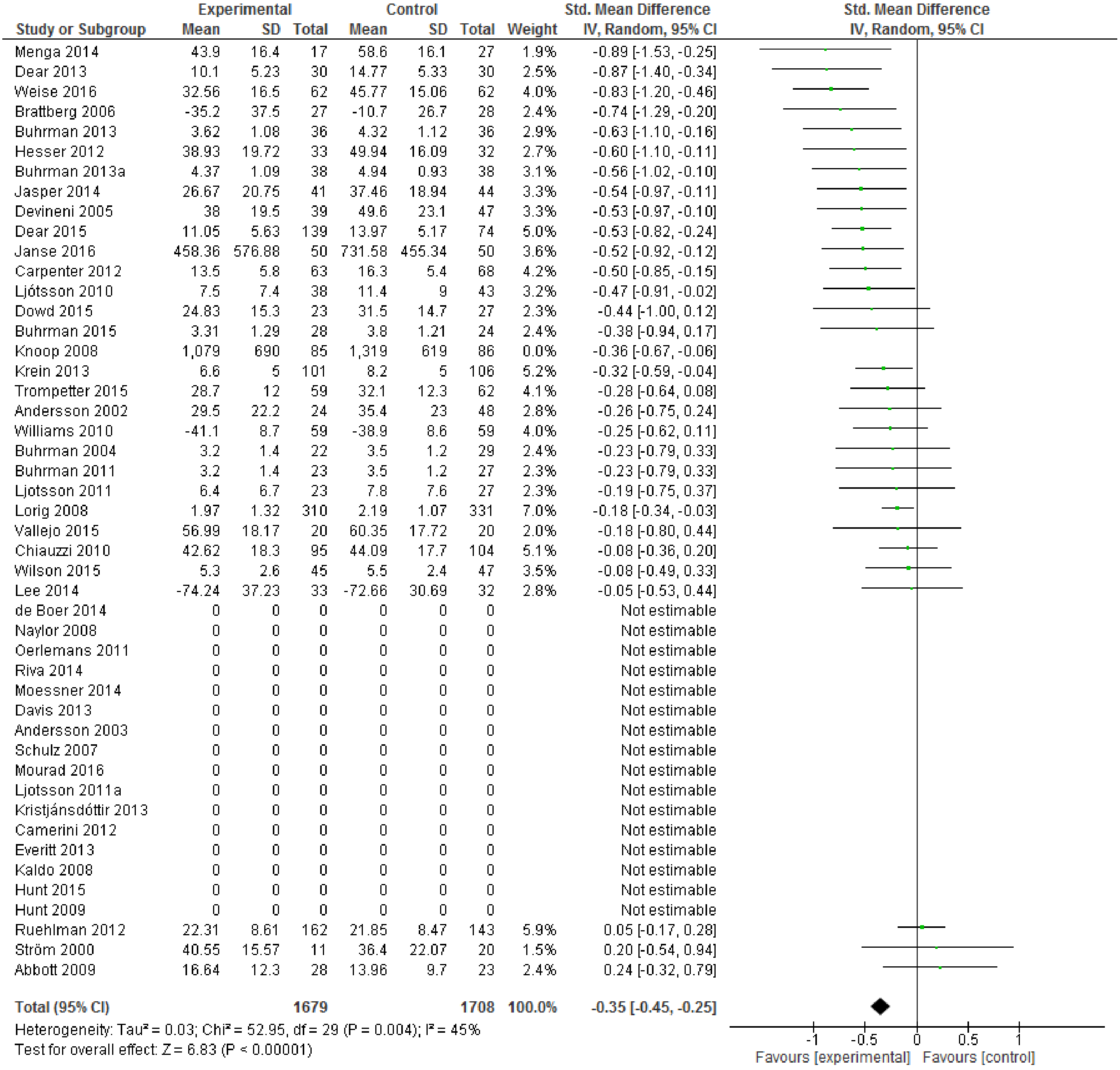

Figure F: Funnel plot all studies

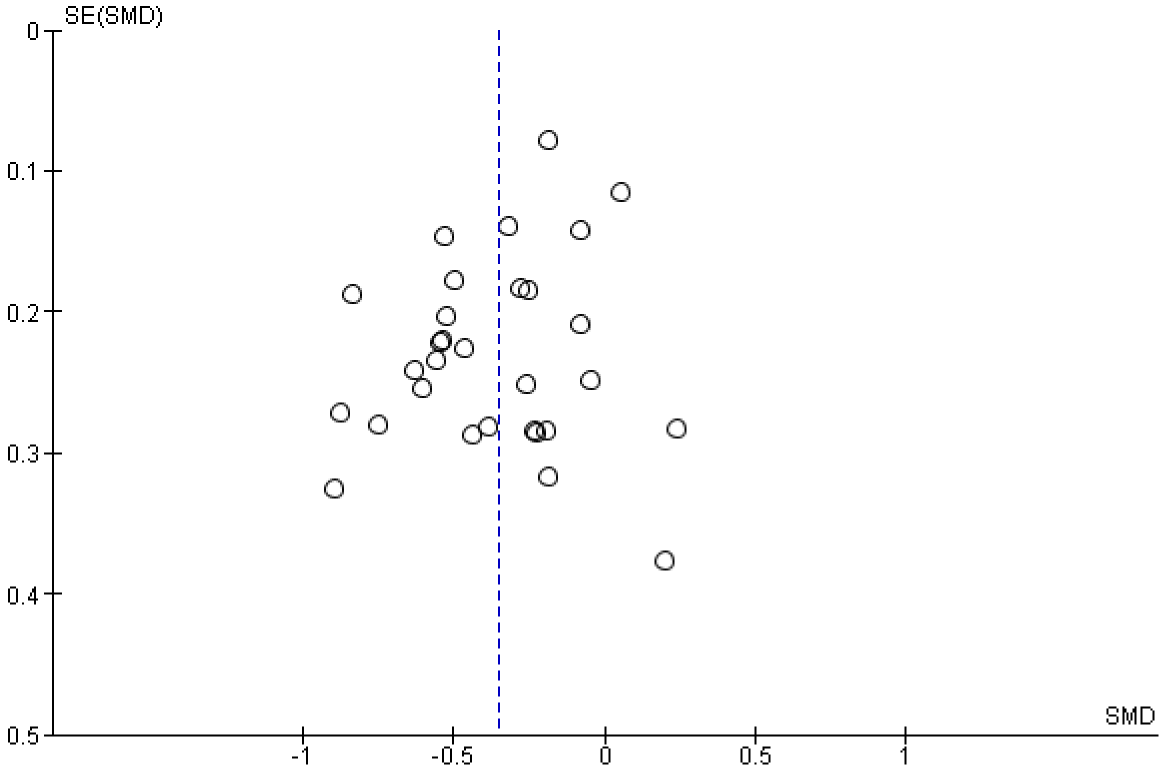

Outcome 1.4: Catastrophizing (post)

Number of eligible studies reporting the outcome: 24
Total number participants: 2900

Table I

	SMD	95% CI	I^2	*P*
All eligible studies (k = 24)	-.41	[-.50, -.31]	28%	.1
SENSITIVITY ANALYSES				
**Internal validity**				
Low risk selection bias (k = 4)	-.34	[-.59, -.1]	41%	.17
Low risk attrition bias (k = 7)	-.54	[-.77, -.31]	51%	.06
Low risk reporting bias (k = 6)	-.43	[-.59, -.26]	37%	.16
Low risk performance bias (k = 11)	-.5	[-.63, -.37]	0%	.46
Low risk due to incomplete data extracted (k = 10)	-.49	[-.62, -.35]	7%	.38
Low risk detection bias				
Low risk other bias (k = 21)	-.4	[-.5, -.3]	31%	.09
**External validity**				
Participants recruited from a general (open) population (k = 16)	-.44	[-.56, -.32]	34%	.09
Participants recruited from a clinical (closed) population (k = 3)	-.16	[.39, .07]	0%	.93

SMD = Standardized mean difference, *CI* = Confidence interval, *P* = P-value for a Chi-square test for Tau; a measure of heterogeneity of standardized mean differences

Figure G: Forest plot all studies

Study or Subgroup	Experimental Mean	Experimental SD	Experimental Total	Control Mean	Control SD	Control Total	Weight	Std. Mean Difference IV, Random, 95% CI
Hunt 2015	44	13.9	17	57	12.9	19	1.7%	-0.95 [-1.64, -0.26]
Buhrman 2015	14.49	9.49	28	22.94	11.65	24	2.4%	-0.79 [-1.36, -0.22]
Weise 2016	-44.02	9.31	62	-36.47	10.39	62	4.8%	-0.76 [-1.13, -0.40]
Hesser 2012	-44.27	9.69	33	-36.81	10.95	32	3.0%	-0.71 [-1.22, -0.21]
Dear 2013	2.01	0.9	30	2.7	1.05	30	2.8%	-0.70 [-1.22, -0.17]
Buhrman 2013	9.56	7.06	36	14.93	8.23	36	3.2%	-0.69 [-1.17, -0.22]
Ljótsson 2010	30.2	17.8	34	41.9	18.7	43	3.4%	-0.63 [-1.09, -0.17]
Hunt 2009	1.9	0.93	13	2.5	0.95	18	1.5%	-0.62 [-1.35, 0.11]
Carpenter 2012	1.6	1	63	2.2	1	68	5.1%	-0.60 [-0.95, -0.25]
Buhrman 2004	8.6	5.2	22	12.3	7.2	29	2.4%	-0.57 [-1.13, -0.00]
Buhrman 2013a	16.08	5.91	38	19	5.56	38	3.4%	-0.50 [-0.96, -0.05]
Wilson 2015	-28.6	12.9	45	-22.5	13.4	47	4.0%	-0.46 [-0.87, -0.05]
Chiauzzi 2010	14.92	12.14	95	21.08	15.2	104	6.8%	-0.44 [-0.73, -0.16]
Dear 2015	-26.79	6.69	139	-23.66	7.66	74	6.7%	-0.44 [-0.73, -0.16]
Trompetter 2015	13.5	11.3	59	17.8	11	62	4.9%	-0.38 [-0.74, -0.02]
Jasper 2014	-47.91	11.7	41	-43.99	13.51	44	3.8%	-0.31 [-0.73, 0.12]
Buhrman 2011	9.5	5.5	23	11.6	8.2	27	2.5%	-0.29 [-0.85, 0.27]
Lorig 2008	-5.69	2.07	310	-5.14	2.05	331	11.5%	-0.27 [-0.42, -0.11]
Vallejo 2015	24.63	5.11	20	26.77	10.33	20	2.1%	-0.26 [-0.88, 0.37]
Ljotsson 2011	6.4	6.7	23	7.8	7.6	27	2.5%	-0.19 [-0.75, 0.37]
Krein 2013	13.2	6	101	14	5.9	106	7.0%	-0.13 [-0.41, 0.14]
Dowd 2015	12.93	8.57	23	14.12	9.12	27	2.5%	-0.13 [-0.69, 0.42]
Ruehlman 2012	8.18	5.45	162	8.77	5.19	143	8.6%	-0.11 [-0.34, 0.11]
Oerlemans 2011	23.57	15.1618	36	24.13	15.0731	36	3.4%	-0.04 [-0.50, 0.43]
Menga 2014	0	0	0	0	0	0		Not estimable
Everitt 2013	0	0	0	0	0	0		Not estimable
Janse 2016	0	0	0	0	0	0		Not estimable
Kaldo 2008	0	0	0	0	0	0		Not estimable
Williams 2010	0	0	0	0	0	0		Not estimable
Mourad 2016	0	0	0	0	0	0		Not estimable
Naylor 2008	0	0	0	0	0	0		Not estimable
Riva 2014	0	0	0	0	0	0		Not estimable
Lee 2014	0	0	0	0	0	0		Not estimable
Kristjánsdóttir 2013	0	0	0	0	0	0		Not estimable
Ljotsson 2011a	0	0	0	0	0	0		Not estimable
Moessner 2014	0	0	0	0	0	0		Not estimable
Andersson 2002	0	0	0	0	0	0		Not estimable
Abbott 2009	0	0	0	0	0	0		Not estimable
Ström 2000	0	0	0	0	0	0		Not estimable
Schulz 2007	0	0	0	0	0	0		Not estimable
Brattberg 2006	0	0	0	0	0	0		Not estimable
Camerini 2012	0	0	0	0	0	0		Not estimable
Devineni 2005	0	0	0	0	0	0		Not estimable
Andersson 2003	0	0	0	0	0	0		Not estimable
Davis 2013	0	0	0	0	0	0		Not estimable
de Boer 2014	0	0	0	0	0	0		Not estimable
**Total (95% CI)**			**1453**			**1447**	**100.0%**	**-0.41 [-0.50, -0.31]**

Heterogeneity: Tau² = 0.01; Chi² = 32.10, df = 23 (P = 0.10); I² = 28%
Test for overall effect: Z = 8.37 (P < 0.00001)

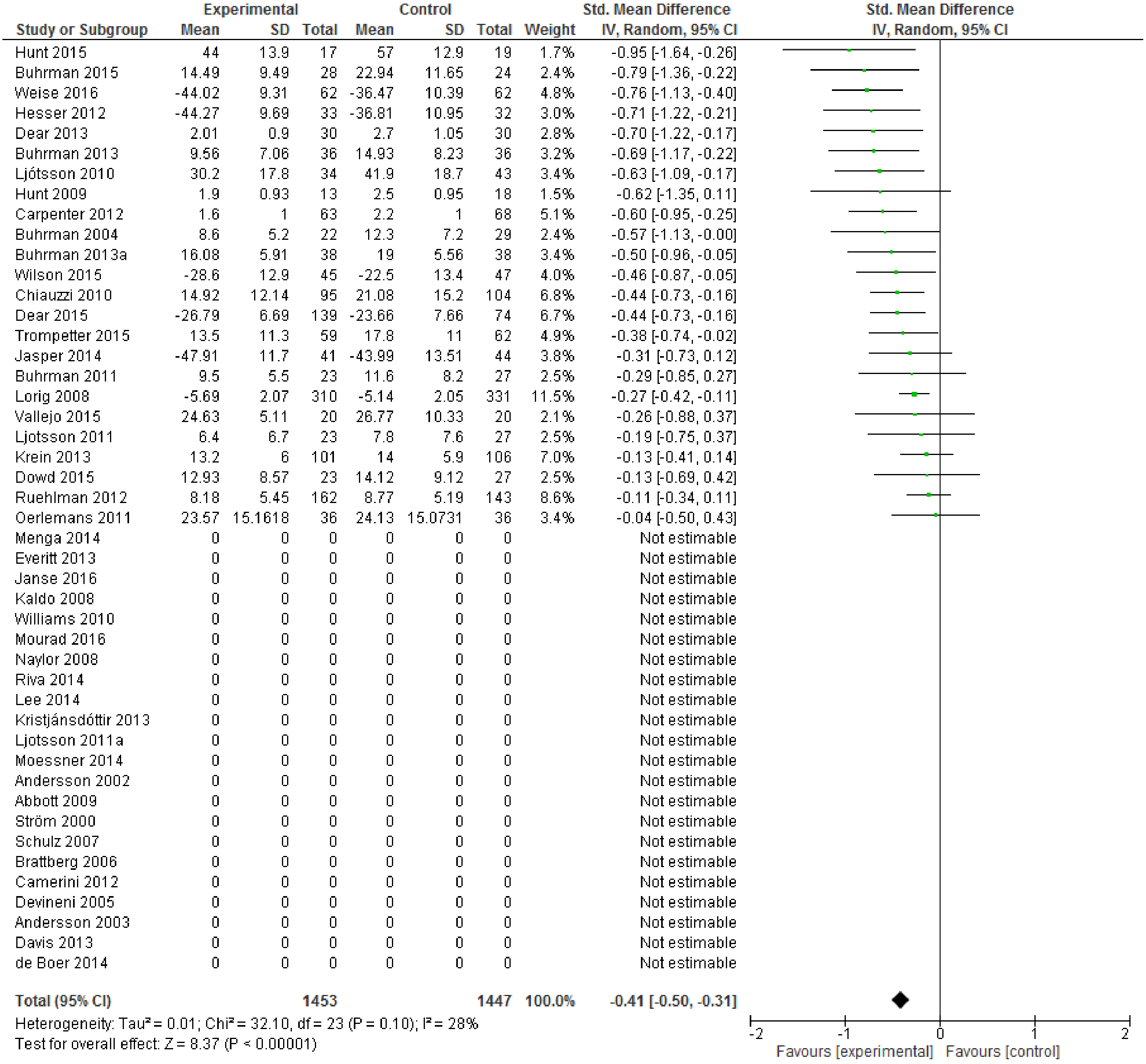

Figure H: Funnel plot all studies

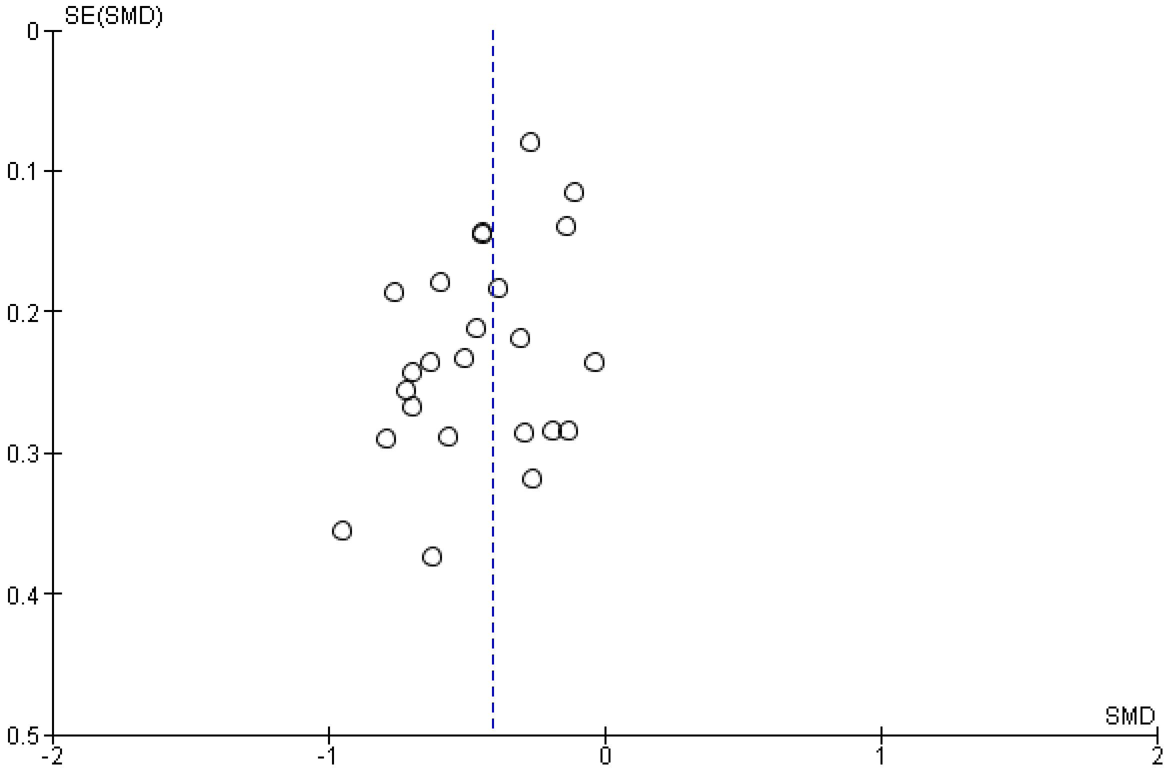

Outcome 1.5: Depression (post)
Number of eligible studies reporting the outcome: 24
Total number participants: 2221

Table J

	SMD	**95% CI**	**I^2**	***P***
All eligible studies (k = 24)	-.18	[-.28, -.07]	29%	.1
SENSITIVITY ANALYSES				
**Internal validity**				
Low risk selection bias (k = 3)	-.25	[-.65, .14]	61%	.08
Low risk attrition bias (k = 8)*	-.42	[-.59, -.26]	0%	.95
Low risk reporting bias (k = 8)	-.24	[-.45, -.02]	51%	.04
Low risk performance bias (k = 11)	-.22	[-.35, -.08]	3%	.41
Low risk due to incomplete data extracted (k = 10)	-.25	[-.4, -.11]	31%	.16
Low risk detection bias				
Low risk other bias (k = 21)	-.18	[-.29, -.07]	38%	.04
**External validity**				
Participants recruited from a general (open) population (k = 15)*	-.2	[-.32, -.08]	20%	.23
Participants recruited from a clinical (closed) population (k = 5)*	-.03	[-.4, .35]	65%	.02

SMD = Standardized mean difference, *CI* = Confidence interval, *P* = P-value for a Chi-square test for Tau; a measure of heterogeneity of standardized mean differences
* Test for subgroup differences: attrition bias (low vs. unclear or high risk) $Chi^2 = 13.27$, df = 1 (P = 0.0003), $I^2 = 92.5\%$; open vs. closed Test for subgroup differences: $Chi^2 = 0.76$, df = 1 (P = 0.38), $I^2 = 0\%$.

Figure I: Forest plot

	Experimental			Control				Std. Mean Difference
**Study or Subgroup**	**Mean**	**SD**	**Total**	**Mean**	**SD**	**Total**	**Weight**	**IV, Random, 95% CI**
Dear 2013	7.55	5.54	30	11.32	5.93	30	3.2%	-0.65 [-1.17, -0.13]
Brattberg 2006	13	10.7	27	19.5	11.2	28	3.0%	-0.58 [-1.13, -0.04]
Janse 2016	116.44	26.17	50	131.86	27.36	50	4.7%	-0.57 [-0.97, -0.17]
Ljótsson 2010	6.9	8.1	38	10.5	8.6	43	4.1%	-0.43 [-0.87, 0.02]
Buhrman 2013a	8.85	4.4	38	10.52	3.77	38	3.9%	-0.40 [-0.86, 0.05]
Hesser 2012	3.48	2.43	33	4.59	3.29	32	3.5%	-0.38 [-0.87, 0.11]
Weise 2016	5.27	3.72	62	6.66	3.98	62	5.5%	-0.36 [-0.71, -0.00]
Jasper 2014	4.41	3.72	41	5.88	4.41	44	4.3%	-0.36 [-0.78, 0.07]
Buhrman 2013	6.95	4.07	36	8.19	3.68	36	3.8%	-0.32 [-0.78, 0.15]
Buhrman 2011	4.9	3.6	23	6.3	5.2	27	2.8%	-0.30 [-0.86, 0.26]
Buhrman 2015	15.77	7.79	28	17.95	6.51	24	2.9%	-0.30 [-0.85, 0.25]
Andersson 2002	5.2	4.1	24	6	3.79	48	3.5%	-0.20 [-0.69, 0.29]
Trompetter 2015	5.1	3.7	59	5.8	3.5	62	5.5%	-0.19 [-0.55, 0.16]
Ström 2000	6.93	7.41	14	7.86	4.85	22	2.1%	-0.15 [-0.82, 0.52]
Dowd 2015	13.5	5.45	23	14.05	5.6	27	2.9%	-0.10 [-0.65, 0.46]
Mourad 2016	5	3.4	7	5.6	7.4	8	1.0%	-0.10 [-1.11, 0.92]
Williams 2010	16.4	11.9	59	17.5	11.5	59	5.4%	-0.09 [-0.45, 0.27]
Wilson 2015	10.1	6.4	45	10.6	5.7	47	4.6%	-0.08 [-0.49, 0.33]
Chiauzzi 2010	11.15	10.52	95	11.44	9.99	104	7.4%	-0.03 [-0.31, 0.25]
Lorig 2008	0	0	0	0	0	0		Not estimable
Hunt 2009	0	0	0	0	0	0		Not estimable
Menga 2014	0	0	0	0	0	0		Not estimable
Moessner 2014	0	0	0	0	0	0		Not estimable
Krein 2013	0	0	0	0	0	0		Not estimable
Hunt 2015	0	0	0	0	0	0		Not estimable
Kaldo 2008	0	0	0	0	0	0		Not estimable
Andersson 2003	0	0	0	0	0	0		Not estimable
Camerini 2012	0	0	0	0	0	0		Not estimable
Carpenter 2012	0	0	0	0	0	0		Not estimable
Devineni 2005	0	0	0	0	0	0		Not estimable
Kristjánsdóttir 2013	0	0	0	0	0	0		Not estimable
Lee 2014	0	0	0	0	0	0		Not estimable
Ljotsson 2011	0	0	0	0	0	0		Not estimable
Ljotsson 2011a	0	0	0	0	0	0		Not estimable
Oerlemans 2011	0	0	0	0	0	0		Not estimable
Riva 2014	0	0	0	0	0	0		Not estimable
Davis 2013	0	0	0	0	0	0		Not estimable
de Boer 2014	0	0	0	0	0	0		Not estimable
Vallejo 2015	0	0	0	0	0	0		Not estimable
Naylor 2008	0	0	0	0	0	0		Not estimable
Schulz 2007	0	0	0	0	0	0		Not estimable
Dear 2015	11.25	4.86	139	11.04	5.25	74	7.3%	0.04 [-0.24, 0.32]
Ruehlman 2012	22.37	12.51	162	21.49	12.61	143	9.0%	0.07 [-0.16, 0.29]
Buhrman 2004	6	4.7	22	5.4	4	29	2.9%	0.14 [-0.42, 0.69]
Everitt 2013	5.59	3.68	39	4.63	3.56	39	4.0%	0.26 [-0.18, 0.71]
Abbott 2009	4.61	5.3	28	2.43	4.3	23	2.8%	0.44 [-0.12, 1.00]
**Total (95% CI)**			**1122**			**1099**	**100.0%**	**-0.18 [-0.28, -0.07]**

Heterogeneity: Tau² = 0.02; Chi² = 32.21, df = 23 (P = 0.10); I² = 29%
Test for overall effect: Z = 3.30 (P = 0.0010)

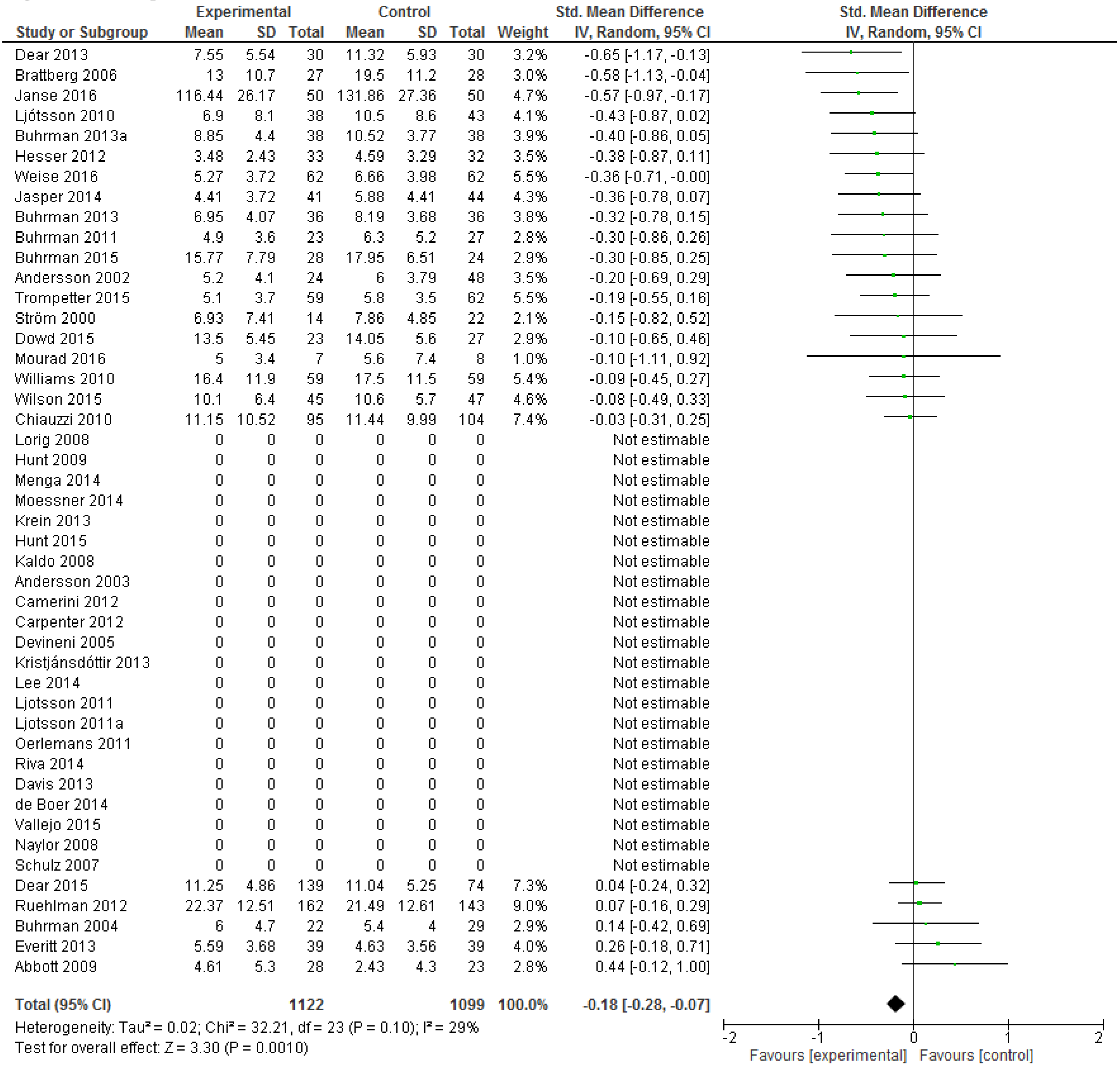

Figure J: Funnel plot all studies

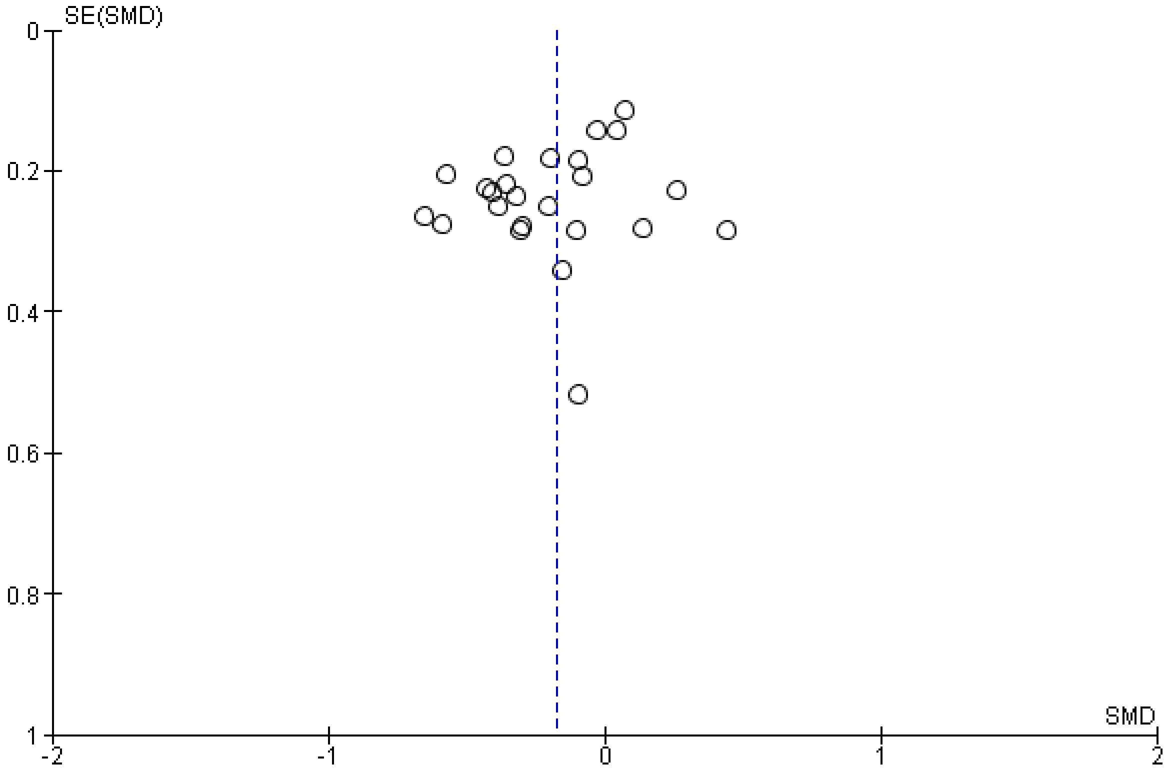

Outcome 1.6: Symptom intensity (6 or more months at follow-up)
Number of eligible studies reporting the outcome: 4
Total number participants: 1015

Table K

	SMD	95% CI	I^2	*P*
All eligible studies (k = 4)	-.18	[-.30, -.05]	0%	.52

SMD = Standardized mean difference, *CI* = Confidence interval, *P* = P-value for a Chi-square test for Tau; a measure of heterogeneity of standardized mean differences

Figure K: Forest plot all studies

	Experimental			Control				Std. Mean Difference
Study or Subgroup	Mean	SD	Total	Mean	SD	Total	Weight	IV, Random, 95% CI
Trompetter 2015	4.9	2.3	82	5.6	2.1	77	15.6%	-0.32 [-0.63, -0.00]
Chiauzzi 2010	4.39	3.02	88	5.26	2.86	67	15.0%	-0.29 [-0.61, 0.03]
Lorig 2008	5.77	2.53	307	6.1	2.35	344	64.5%	-0.14 [-0.29, 0.02]
Moessner 2014	0	0	0	0	0	0		Not estimable
Naylor 2010	0	0	0	0	0	0		Not estimable
Hesser 2012	0	0	0	0	0	0		Not estimable
Jasper 2014	0	0	0	0	0	0		Not estimable
Brattberg 2007	0	0	0	0	0	0		Not estimable
Vallejo 2015	0	0	0	0	0	0		Not estimable
Ljotsson 2011a	0	0	0	0	0	0		Not estimable
Kristjánsdóttir 2013a	0	0	0	0	0	0		Not estimable
Dowd 2015	4.81	2.01	23	4.69	1.97	27	4.9%	0.06 [-0.50, 0.62]
**Total (95% CI)**			**500**			**515**	**100.0%**	**-0.18 [-0.30, -0.05]**

Heterogeneity: Tau² = 0.00; Chi² = 2.24, df = 3 (P = 0.52); I² = 0%
Test for overall effect: Z = 2.81 (P = 0.005)

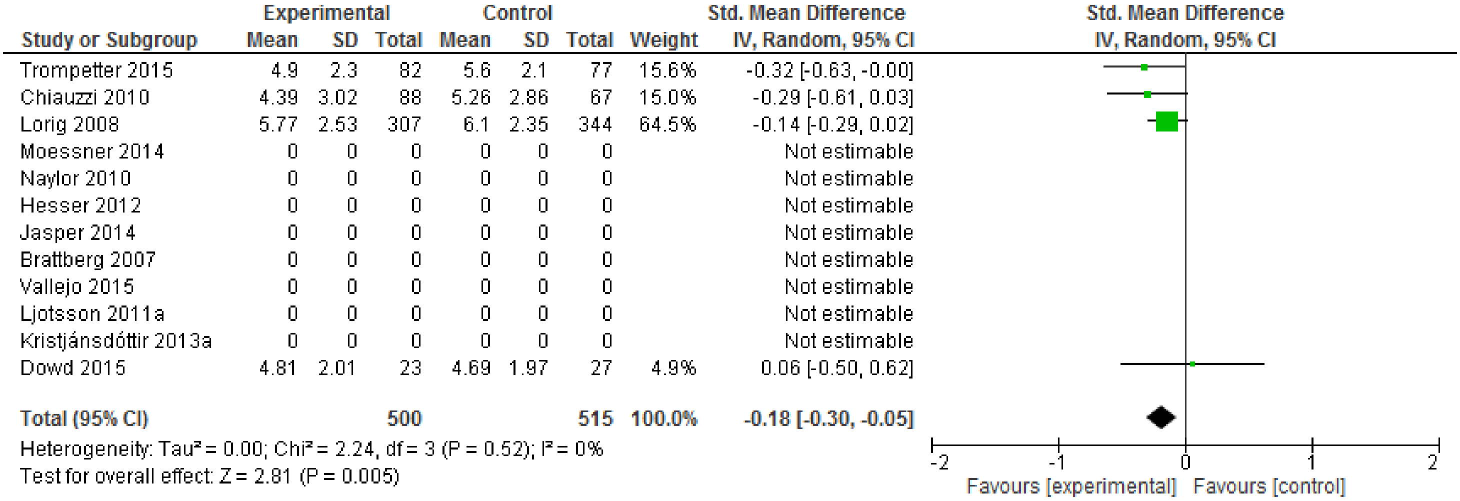

Outcome 1.7 HRQOL (6 or more months at follow-up)
Number of eligible studies reporting the outcome: 1
Total number participants: 651

Table L

	SMD	**95% CI**	I^2	***P***
All eligible studies (k = 1; Lorig 2008)	.13	[-.02, -.28]	/	/

SMD = Standardized mean difference, *CI* = Confidence interval, *P* = P-value for a Chi-square test for Tau; a measure of heterogeneity of standardized mean differences

Outcome 1.8 Functional interference (6 or more months at follow-up)
Number of eligible studies reporting the outcome: 4
Total number participants: 1015

Table M

	SMD	**95% CI**	**I^2**	***P***
All eligible studies (k = 4)	-.19	[-.31, -.06]	0%	.55

SMD = Standardized mean difference, *CI* = Confidence interval, *P* = P-value for a Chi-square test for Tau; a measure of heterogeneity of standardized mean differences

Figure L: Forrest plot all studies

	Experimental			Control				Std. Mean Difference
**Study or Subgroup**	**Mean**	**SD**	**Total**	**Mean**	**SD**	**Total**	**Weight**	**IV, Random, 95% CI**
Dowd 2015	30.71	14.39	23	35.47	11.9	27	4.9%	-0.36 [-0.92, 0.20]
Trompetter 2015	27.2	12	82	30.6	11.3	77	15.7%	-0.29 [-0.60, 0.02]
Lorig 2008	1.9	1.15	307	2.11	1.04	344	64.3%	-0.19 [-0.35, -0.04]
Chiauzzi 2010	44.51	17.03	67	44.53	17.54	88	15.2%	-0.00 [-0.32, 0.32]
Vallejo 2015	0	0	0	0	0	0		Not estimable
**Total (95% CI)**			**479**			**536**	**100.0%**	**-0.19 [-0.31, -0.06]**

Heterogeneity: Tau² = 0.00; Chi² = 2.09, df = 3 (P = 0.55); I² = 0%
Test for overall effect: Z = 2.95 (P = 0.003)

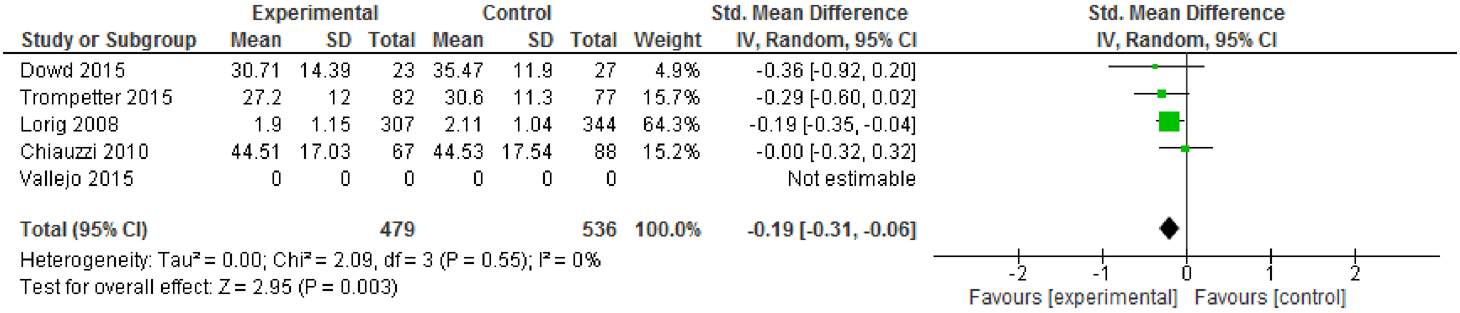

Outcome 1.9 Catastrophizing (6 or more months at follow-up)
Number of eligible studies reporting the outcome: 4
Total number participants: 1015

Table N

	SMD	**95% CI**	**I^2**	***P***
All eligible studies (k = 4)	-.32	[-.47, -.17]	19%	.30

SMD = Standardized mean difference, *CI* = Confidence interval, *P* = P-value for a Chi-square test for Tau; a measure of heterogeneity of standardized mean differences

Figure M: Forrest plot all studies

	Experimental			Control				Std. Mean Difference
**Study or Subgroup**	**Mean**	**SD**	**Total**	**Mean**	**SD**	**Total**	**Weight**	**IV, Random, 95% CI**
Chiauzzi 2010	5.05	4.69	67	7.56	4.58	88	19.0%	-0.54 [-0.86, -0.22]
Trompetter 2015	11.9	11.9	82	16.3	11.1	77	20.0%	-0.38 [-0.69, -0.07]
Lorig 2008	-5.89	2.09	307	-5.34	2.06	344	53.8%	-0.26 [-0.42, -0.11]
Vallejo 2015	0	0	0	0	0	0		Not estimable
Dowd 2015	13.28	8.44	23	13.22	8.37	27	7.2%	0.01 [-0.55, 0.56]
**Total (95% CI)**			**479**			**536**	**100.0%**	**-0.32 [-0.47, -0.17]**

Heterogeneity: Tau² = 0.01; Chi² = 3.70, df = 3 (P = 0.30); I² = 19%
Test for overall effect: Z = 4.07 (P < 0.0001)

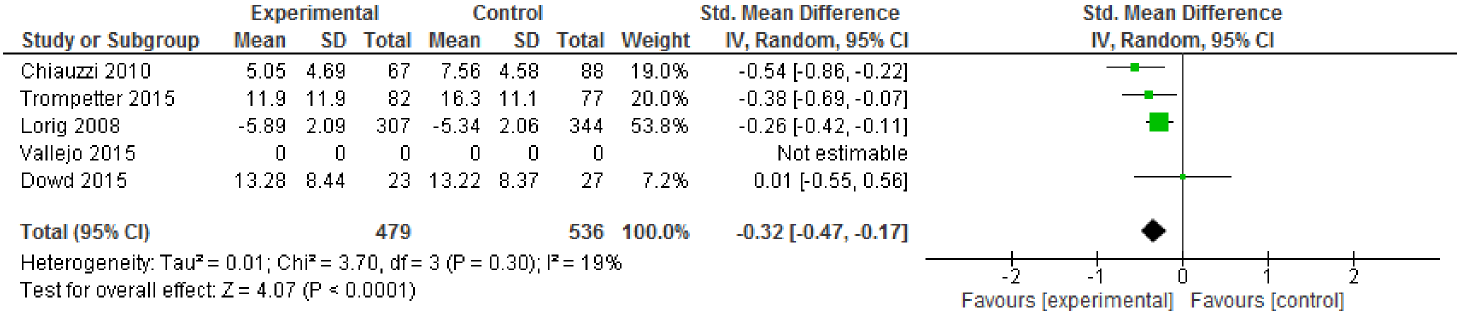

Outcome 1.10: Depression (6 or more months at follow-up)
Number of eligible studies reporting the outcome: 4
Total number participants: 416

Table O

	SMD	95% CI	I^2	*P*
All eligible studies (k = 4)	-.29	[-.48, -.10]	0%	.59

SMD = Standardized mean difference, *CI* = Confidence interval, *P* = P-value for a Chi-square test for Tau; a measure of heterogeneity of standardized mean differences

Figure N: Forrest plot all studies

	Experimental			Control				Std. Mean Difference
Study or Subgroup	Mean	SD	Total	Mean	SD	Total	Weight	IV, Random, 95% CI
Jasper 2014	0	0	0	0	0	0		Not estimable
Vallejo 2015	0	0	0	0	0	0		Not estimable
Hesser 2012	0	0	0	0	0	0		Not estimable
Lorig 2008	0	0	0	0	0	0		Not estimable
Ljotsson 2011a	0	0	0	0	0	0		Not estimable
Naylor 2010	0	0	0	0	0	0		Not estimable
Moessner 2014	0	0	0	0	0	0		Not estimable
Kristjánsdóttir 2013a	0	0	0	0	0	0		Not estimable
Dowd 2015	14.15	1.1	23	14.78	1	27	11.6%	-0.59 [-1.16, -0.02]
Brattberg 2007	6.7	3.8	25	7.8	4.8	25	12.1%	-0.25 [-0.81, 0.31]
Trompetter 2015	4.3	3.8	53	5.7	3.6	64	27.9%	-0.38 [-0.74, -0.01]
Chiauzzi 2010	10.55	12.09	95	12.65	11.42	104	48.4%	-0.18 [-0.46, 0.10]
**Total (95% CI)**			**196**			**220**	**100.0%**	**-0.29 [-0.48, -0.10]**

Heterogeneity: Tau² = 0.00; Chi² = 1.94, df = 3 (P = 0.59); I² = 0%
Test for overall effect: Z = 2.93 (P = 0.003)

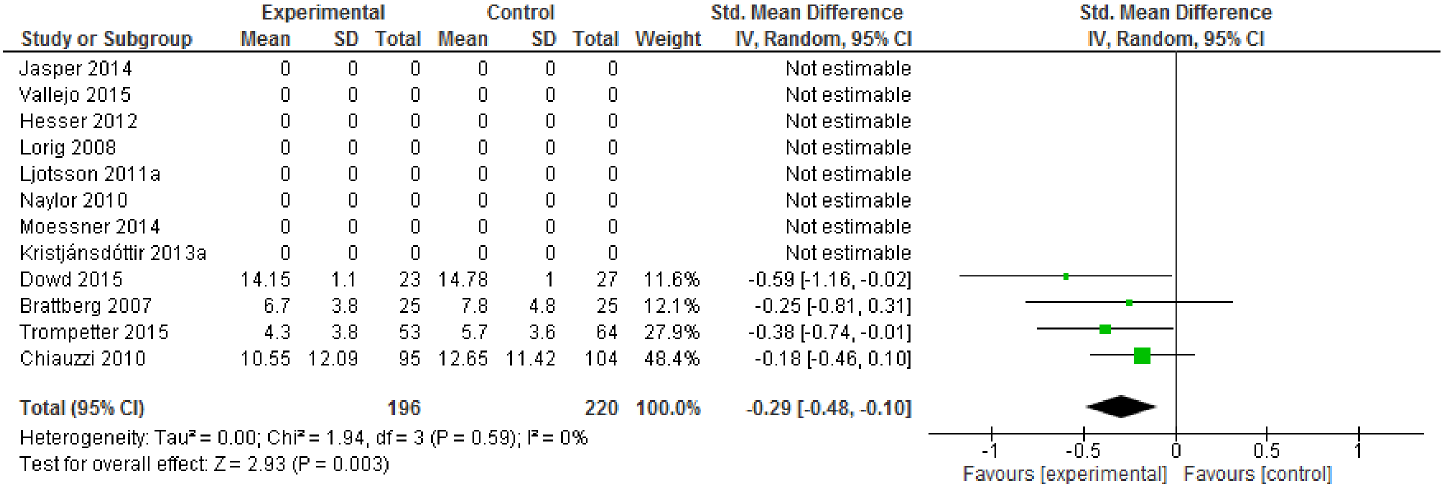

Comparison 2: Computer based versus active control (tables P-Y, and figs O-AB)

Outcome 2.1: Symptom intensity (post)

Number of eligible studies reporting the outcome: 11

Total number participants: 1292

Table P

	SMD	**95% CI**	**I^2**	***P***
All eligible studies (k = 11)	-.16	[-.35, .02]	56%	.01
SENSITIVITY ANALYSES				
**Internal validity**				
Low risk selection bias (k = 4)*[1]	-.33	[-.66, .01]	71%	.002
Low risk attrition bias (k = 5)	-.2	[-.61, .2]	73%	.005
Low risk reporting bias (k = 3)	-.26	[-.44, -.07]	0%	.38
Low risk performance bias (k = 5)	-.29	[-.63, .04]	60%	.04
Low risk due to incomplete data extracted (k = 2)	-.60	[-1.3, 0.1]	80%	.03
Low risk detection bias				
Low risk other bias (k = 8)	-.18	[-.39, .04]	68%	.003
**External validity**				
Participants recruited from a general (open) population (k = 3)	-.31	[-.51, -.12]	0%	.92
Participants recruited from a clinical (closed) population (k = 5)	-.08	[-.44, .28]	70%	.01

SMD = Standardized mean difference, *CI* = Confidence interval, *P* = P-value for a Chi-square test for Tau; a measure of heterogeneity of standardized mean differences

*[1] 1 study had risk of bias due to dissimilarity at baseline for symptom intensity (Naylor 2008)

Figure O: Forest plot all studies

	Experimental			Control				Std. Mean Difference
Study or Subgroup	Mean	SD	Total	Mean	SD	Total	Weight	IV, Random, 95% CI
Naylor 2008	3.3	1.7	26	5.2	2	25	6.3%	-1.01 [-1.60, -0.42]
Trompetter 2015	4.9	2.3	59	5.8	2.4	51	10.2%	-0.38 [-0.76, -0.00]
Ljotsson 2011a	36.5	12.7	96	41.1	12.4	90	12.5%	-0.36 [-0.65, -0.07]
Andersson 2003	2	1.1	17	2.3	0.8	13	4.7%	-0.30 [-1.02, 0.43]
Dear 2015	4.68	1.79	139	5.2	1.8	135	14.0%	-0.29 [-0.53, -0.05]
Kaldo 2008	4.2	1.5	24	4.5	2	20	6.2%	-0.17 [-0.76, 0.43]
Everitt 2013	237.3	100.78	45	237.44	85.36	43	9.3%	-0.00 [-0.42, 0.42]
Abbott 2009	0	0	0	0	0	0		Not estimable
Williams 2010	0	0	0	0	0	0		Not estimable
Weise 2016	0	0	0	0	0	0		Not estimable
Wilson 2015	0	0	0	0	0	0		Not estimable
Vallejo 2015	0	0	0	0	0	0		Not estimable
Schulz 2007	0	0	0	0	0	0		Not estimable
Mourad 2016	0	0	0	0	0	0		Not estimable
Ruehlman 2012	0	0	0	0	0	0		Not estimable
Ström 2000	0	0	0	0	0	0		Not estimable
Menga 2014	0	0	0	0	0	0		Not estimable
Oerlemans 2011	0	0	0	0	0	0		Not estimable
Lee 2014	0	0	0	0	0	0		Not estimable
Lorig 2008	0	0	0	0	0	0		Not estimable
Krein 2013	0	0	0	0	0	0		Not estimable
Ljótsson 2010	0	0	0	0	0	0		Not estimable
Hunt 2015	0	0	0	0	0	0		Not estimable
Ljotsson 2011	0	0	0	0	0	0		Not estimable
Hesser 2012	0	0	0	0	0	0		Not estimable
Jasper 2014	0	0	0	0	0	0		Not estimable
Dowd 2015	0	0	0	0	0	0		Not estimable
Janse 2016	0	0	0	0	0	0		Not estimable
Dear 2013	0	0	0	0	0	0		Not estimable
Hunt 2009	0	0	0	0	0	0		Not estimable
Chiauzzi 2010	0	0	0	0	0	0		Not estimable
Devineni 2005	0	0	0	0	0	0		Not estimable
Davis 2013	0	0	0	0	0	0		Not estimable
Buhrman 2015	0	0	0	0	0	0		Not estimable
Buhrman 2013a	0	0	0	0	0	0		Not estimable
Carpenter 2012	0	0	0	0	0	0		Not estimable
Camerini 2012	0	0	0	0	0	0		Not estimable
Buhrman 2004	0	0	0	0	0	0		Not estimable
Brattberg 2006	0	0	0	0	0	0		Not estimable
Buhrman 2013	0	0	0	0	0	0		Not estimable
Buhrman 2011	0	0	0	0	0	0		Not estimable
Andersson 2002	0	0	0	0	0	0		Not estimable
Moessner 2014	3.74	2.09	167	3.64	2.03	161	14.6%	0.05 [-0.17, 0.26]
de Boer 2014	5.53	2.19	20	5.32	2.18	23	6.1%	0.09 [-0.51, 0.69]
Kristjánsdóttir 2013	54.14	24.06	47	50.56	23.37	40	9.2%	0.15 [-0.27, 0.57]
Riva 2014	2.8	2	27	2.1	2	24	6.8%	0.34 [-0.21, 0.90]
**Total (95% CI)**			**667**			**625**	**100.0%**	**-0.16 [-0.35, 0.02]**

Heterogeneity: $Tau^2 = 0.05$; $Chi^2 = 22.62$, df = 10 ($P = 0.01$); $I^2 = 56\%$
Test for overall effect: $Z = 1.76$ ($P = 0.08$)

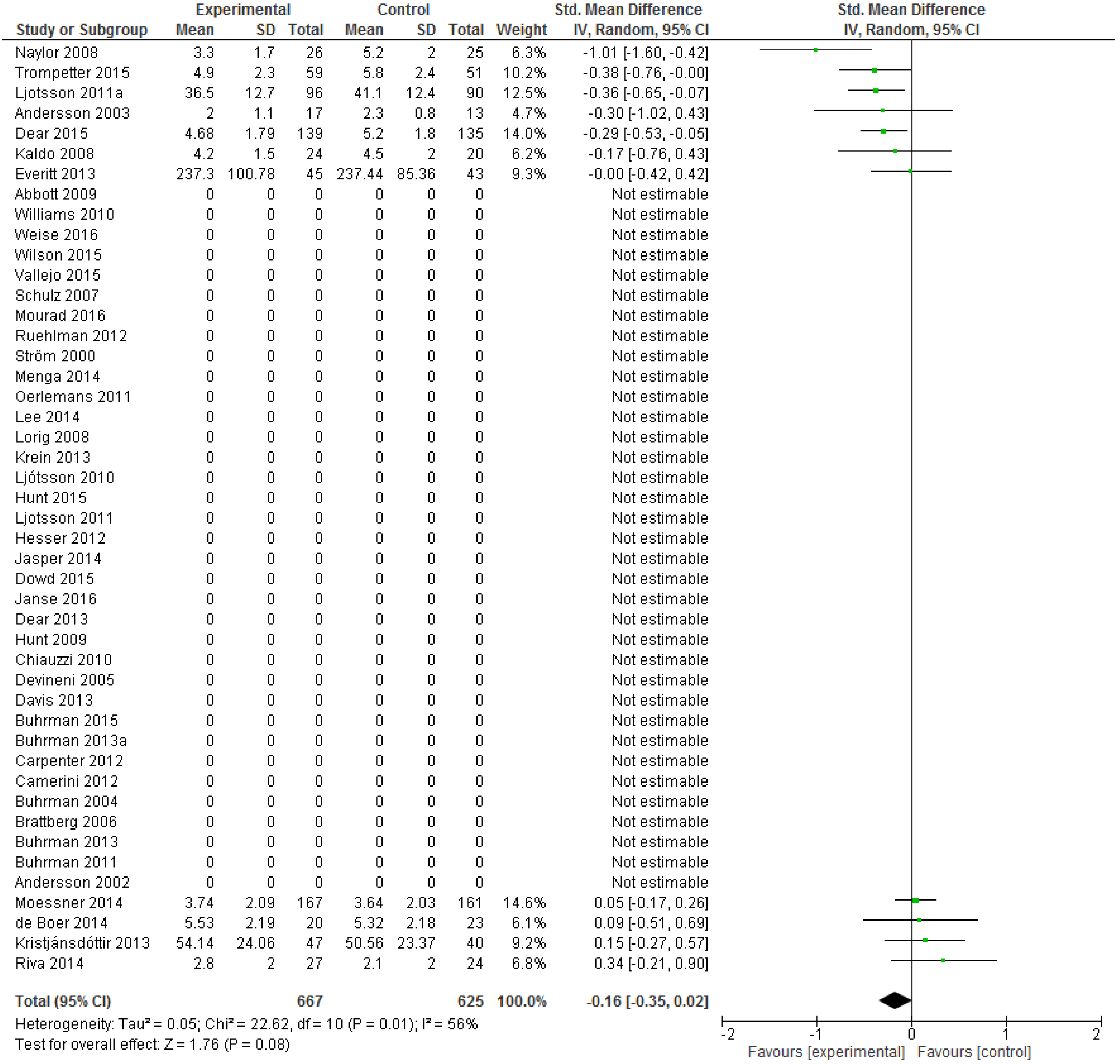

Figure P: Funnel plot

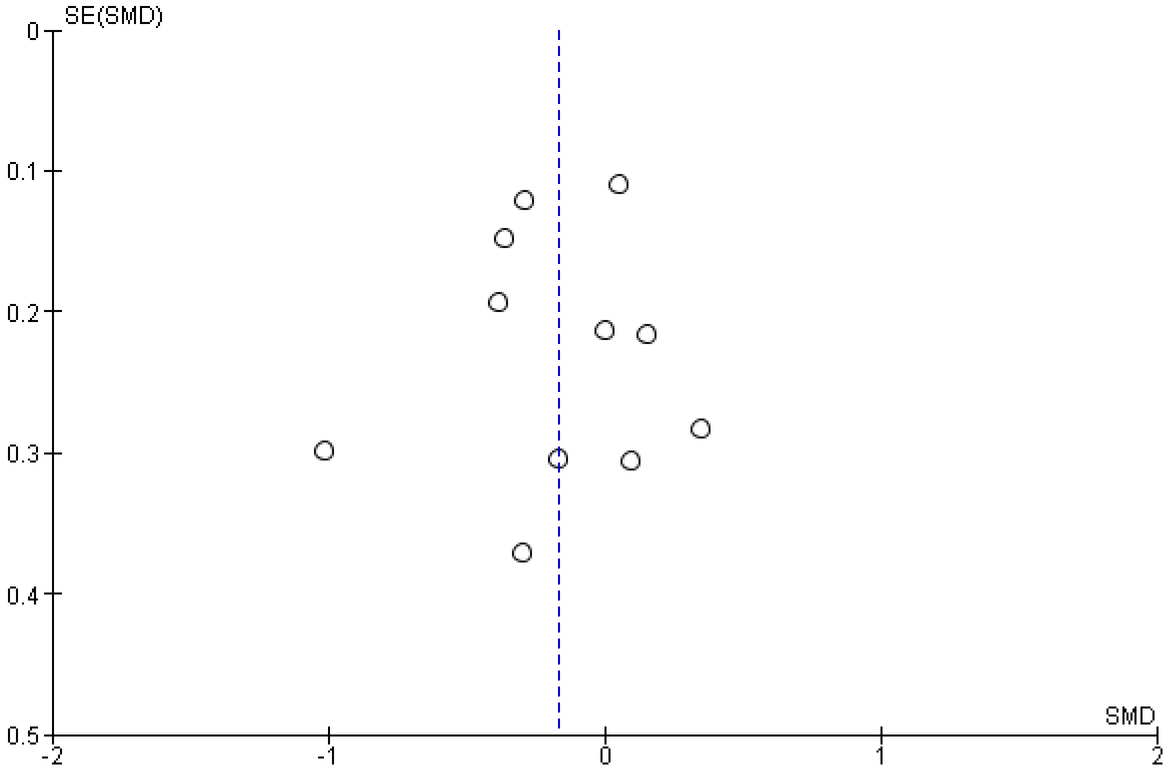

Outcome 2.2: Health-related Quality Of Life (post)
Number of eligible studies reporting the outcome: 6
Total number participants: 761

Table Q

	SMD	**95% CI**	**I^2**	***P***
All eligible studies (k = 6)	-.17	[-.48, .14]	74%	.002
SENSITIVITY ANALYSES				
**Internal validity**				
Low risk selection bias (k = 3)	-.34	[-.89, .21]	78%	.01
Low risk attrition bias (k = 3)	-.34	[-.89, .21]	78%	.01
Low risk reporting bias (k = 1)	/	/	/	/
Low risk performance bias (k = 2)	-.24	[-1.27, .78]	86%	.007
Low risk due to incomplete data extracted (k = 1)	/	/	/	/
Low risk detection bias				
Low risk other bias (k = 5)	-.15	[-.50, .21]	79%	<.001
**External validity**				
Participants recruited from a general (open) population (k = 1)	/	/	/	/
Participants recruited from a clinical (closed) population (k = 3)	-.17	[-.55, .22]	69%	.02

SMD = Standardized mean difference, *CI* = Confidence interval, *P* = P-value for a Chi-square test for Tau; a measure of heterogeneity of standardized mean differences

Figure Q: Forest plot all studies

Study or Subgroup	Experimental Mean	Experimental SD	Experimental Total	Control Mean	Control SD	Control Total	Weight	Std. Mean Difference IV, Random, 95% CI
Naylor 2008	-48.8	13.4	26	-38.9	11.6	25	13.4%	-0.78 [-1.35, -0.21]
Ljotsson 2011a	-75.7	17.7	97	-65.7	21.1	94	20.1%	-0.51 [-0.80, -0.22]
de Boer 2014	-53.18	22.69	20	-45.63	21.58	24	12.8%	-0.34 [-0.93, 0.26]
Moessner 2014	-41.91	10.42	166	-41.25	11.29	158	21.8%	-0.06 [-0.28, 0.16]
Oerlemans 2011	0	0	0	0	0	0		Not estimable
Weise 2016	0	0	0	0	0	0		Not estimable
Ström 2000	0	0	0	0	0	0		Not estimable
Ljotsson 2011	0	0	0	0	0	0		Not estimable
Andersson 2002	0	0	0	0	0	0		Not estimable
Trompetter 2015	0	0	0	0	0	0		Not estimable
Williams 2010	0	0	0	0	0	0		Not estimable
Abbott 2009	0	0	0	0	0	0		Not estimable
Vallejo 2015	0	0	0	0	0	0		Not estimable
Wilson 2015	0	0	0	0	0	0		Not estimable
Hunt 2015	0	0	0	0	0	0		Not estimable
Ruehlman 2012	0	0	0	0	0	0		Not estimable
Krein 2013	0	0	0	0	0	0		Not estimable
Hunt 2009	0	0	0	0	0	0		Not estimable
Mourad 2016	0	0	0	0	0	0		Not estimable
Kristjánsdóttir 2013	0	0	0	0	0	0		Not estimable
Schulz 2007	0	0	0	0	0	0		Not estimable
Riva 2014	0	0	0	0	0	0		Not estimable
Dowd 2015	0	0	0	0	0	0		Not estimable
Ljótsson 2010	0	0	0	0	0	0		Not estimable
Dear 2015	0	0	0	0	0	0		Not estimable
Carpenter 2012	0	0	0	0	0	0		Not estimable
Menga 2014	0	0	0	0	0	0		Not estimable
Kaldo 2008	0	0	0	0	0	0		Not estimable
Lorig 2008	0	0	0	0	0	0		Not estimable
Lee 2014	0	0	0	0	0	0		Not estimable
Davis 2013	0	0	0	0	0	0		Not estimable
Buhrman 2015	0	0	0	0	0	0		Not estimable
Buhrman 2013a	0	0	0	0	0	0		Not estimable
Andersson 2003	0	0	0	0	0	0		Not estimable
Devineni 2005	0	0	0	0	0	0		Not estimable
Jasper 2014	0	0	0	0	0	0		Not estimable
Janse 2016	0	0	0	0	0	0		Not estimable
Chiauzzi 2010	0	0	0	0	0	0		Not estimable
Buhrman 2011	0	0	0	0	0	0		Not estimable
Buhrman 2013	0	0	0	0	0	0		Not estimable
Camerini 2012	0	0	0	0	0	0		Not estimable
Buhrman 2004	0	0	0	0	0	0		Not estimable
Brattberg 2006	0	0	0	0	0	0		Not estimable
Dear 2013	0	0	0	0	0	0		Not estimable
Hesser 2012	-2.12	1.47	33	-2.53	1.55	30	15.0%	0.27 [-0.23, 0.77]
Everitt 2013	-67.7	12.6484	45	-71.6	12.3155	43	16.9%	0.31 [-0.11, 0.73]
**Total (95% CI)**			**387**			**374**	**100.0%**	**-0.17 [-0.48, 0.14]**

Heterogeneity: $Tau^2 = 0.10$; $Chi^2 = 18.98$, df = 5 (P = 0.002); $I^2 = 74\%$

Test for overall effect: Z = 1.08 (P = 0.28)

Std. Mean Difference
IV, Random, 95% CI

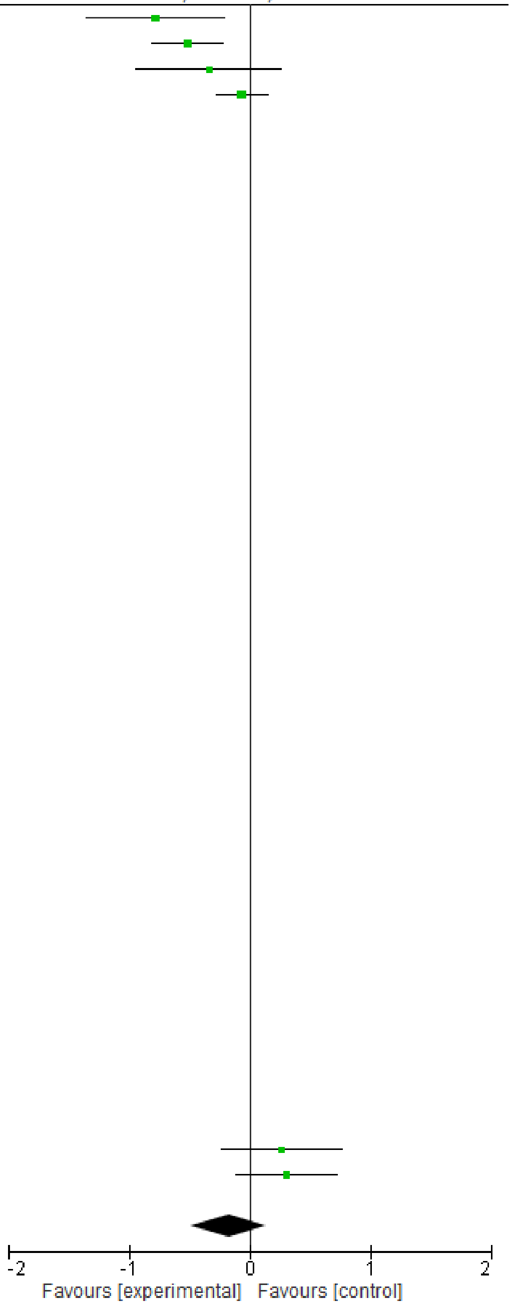

Figure R: Funnel plot

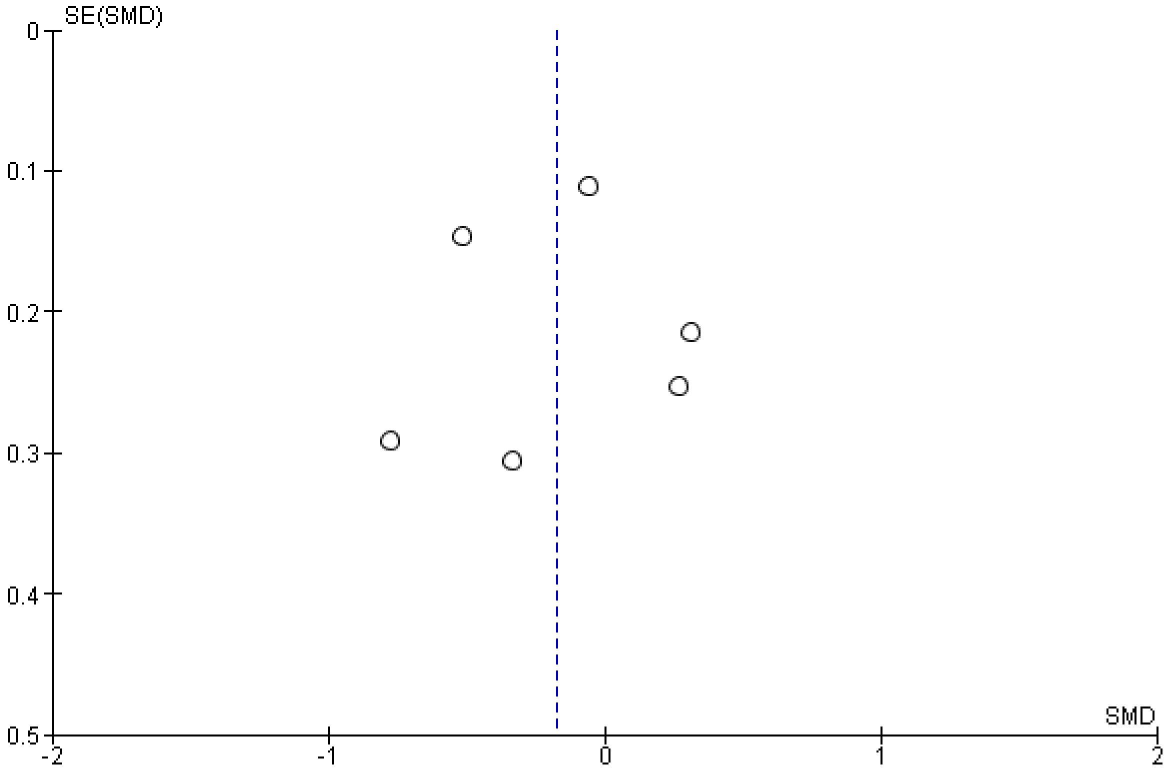

Outcome 2.3: Functional interference (post)

Number of eligible studies reporting the outcome: 10
Total number participants: 1097

Table R

	SMD	**95% CI**	**I^2**	***P*[1]**
All eligible studies (k = 10)	-.15	[-.27, -.03]	0%	.7
SENSITIVITY ANALYSES				
**Internal validity**				
Low risk selection bias (k = 4)	-.17	[-.35, .02]	0%	.52
Low risk attrition bias (k = 4)	-.21	[-.46, .04]	0%	.62
Low risk reporting bias (k = 3)	-.15	[-.37, .08]	0%	.60
Low risk performance bias (k = 8)	-.13	[-.28, .03]	0%	.87
Low risk due to incomplete data extracted (k = 4)	-.09	[-.28, .09]	0%	.75
Low risk detection bias				
Low risk other bias (k = 7)	-.16	[-.29, -.04]	0%	.47
**External validity**				
Participants recruited from a general (open) population (k = 5)	-.18	[-.38, .02]	20%	.29
Participants recruited from a clinical (closed) population (k = 3)	-.1	[-.29, .08]	0%	.68

SMD = Standardized mean difference, *CI* = Confidence interval, *P* = P-value for a Chi-square test for Tau; a measure of heterogeneity of standardized mean differences

Figure S: Forest plot

	Experimental			Control				Std. Mean Difference
Study or Subgroup	Mean	SD	Total	Mean	SD	Total	Weight	IV, Random, 95% CI
Trompetter 2015	27.2	12	59	32.9	12.2	51	9.8%	-0.47 [-0.85, -0.09]
Hesser 2012	31.94	14.54	33	38.93	19.72	30	5.7%	-0.40 [-0.90, 0.10]
Naylor 2008	50.1	12.1	26	54.9	12.2	25	4.6%	-0.39 [-0.94, 0.17]
Kristjánsdóttir 2013	49.12	19.65	47	53.07	18.68	39	7.8%	-0.20 [-0.63, 0.22]
Moessner 2014	11.4	5.67	167	11.87	5.68	159	30.0%	-0.08 [-0.30, 0.13]
Vallejo 2015	56.99	18.17	20	58.2	18.57	20	3.7%	-0.06 [-0.68, 0.56]
Kaldo 2008	28.9	13.5	26	29.9	18.5	25	4.7%	-0.06 [-0.61, 0.49]
Dear 2015	11.05	5.63	139	11.36	5.22	123	24.0%	-0.06 [-0.30, 0.19]
Jasper 2014	26.67	20.75	41	27.7	21.93	43	7.7%	-0.05 [-0.48, 0.38]
Janse 2016	0	0	0	0	0	0		Not estimable
Buhrman 2004	0	0	0	0	0	0		Not estimable
Ljotsson 2011a	0	0	0	0	0	0		Not estimable
Menga 2014	0	0	0	0	0	0		Not estimable
Buhrman 2013a	0	0	0	0	0	0		Not estimable
Carpenter 2012	0	0	0	0	0	0		Not estimable
Buhrman 2013	0	0	0	0	0	0		Not estimable
Camerini 2012	0	0	0	0	0	0		Not estimable
Davis 2013	0	0	0	0	0	0		Not estimable
Lorig 2008	0	0	0	0	0	0		Not estimable
de Boer 2014	0	0	0	0	0	0		Not estimable
Devineni 2005	0	0	0	0	0	0		Not estimable
Krein 2013	0	0	0	0	0	0		Not estimable
Chiauzzi 2010	0	0	0	0	0	0		Not estimable
Ljotsson 2011	0	0	0	0	0	0		Not estimable
Hunt 2015	0	0	0	0	0	0		Not estimable
Williams 2010	0	0	0	0	0	0		Not estimable
Dear 2013	0	0	0	0	0	0		Not estimable
Wilson 2015	0	0	0	0	0	0		Not estimable
Buhrman 2015	0	0	0	0	0	0		Not estimable
Hunt 2009	0	0	0	0	0	0		Not estimable
Ljótsson 2010	0	0	0	0	0	0		Not estimable
Dowd 2015	0	0	0	0	0	0		Not estimable
Brattberg 2007	0	0	0	0	0	0		Not estimable
Brattberg 2006	0	0	0	0	0	0		Not estimable
Lee 2014	0	0	0	0	0	0		Not estimable
Abbott 2009	0	0	0	0	0	0		Not estimable
Andersson 2002	0	0	0	0	0	0		Not estimable
Schulz 2007	0	0	0	0	0	0		Not estimable
Weise 2016	0	0	0	0	0	0		Not estimable
Everitt 2013	0	0	0	0	0	0		Not estimable
Buhrman 2011	0	0	0	0	0	0		Not estimable
Ruehlman 2012	0	0	0	0	0	0		Not estimable
Mourad 2016	0	0	0	0	0	0		Not estimable
Ström 2000	0	0	0	0	0	0		Not estimable
Riva 2014	0	0	0	0	0	0		Not estimable
Oerlemans 2011	0	0	0	0	0	0		Not estimable
Andersson 2003	7.9	5.1	15	7	4.8	9	2.1%	0.17 [-0.65, 1.00]
**Total (95% CI)**			**573**			**524**	**100.0%**	**-0.15 [-0.27, -0.03]**

Heterogeneity: $Tau^2 = 0.00$; $Chi^2 = 6.35$, $df = 9$ ($P = 0.70$); $I^2 = 0\%$

Test for overall effect: $Z = 2.41$ ($P = 0.02$)

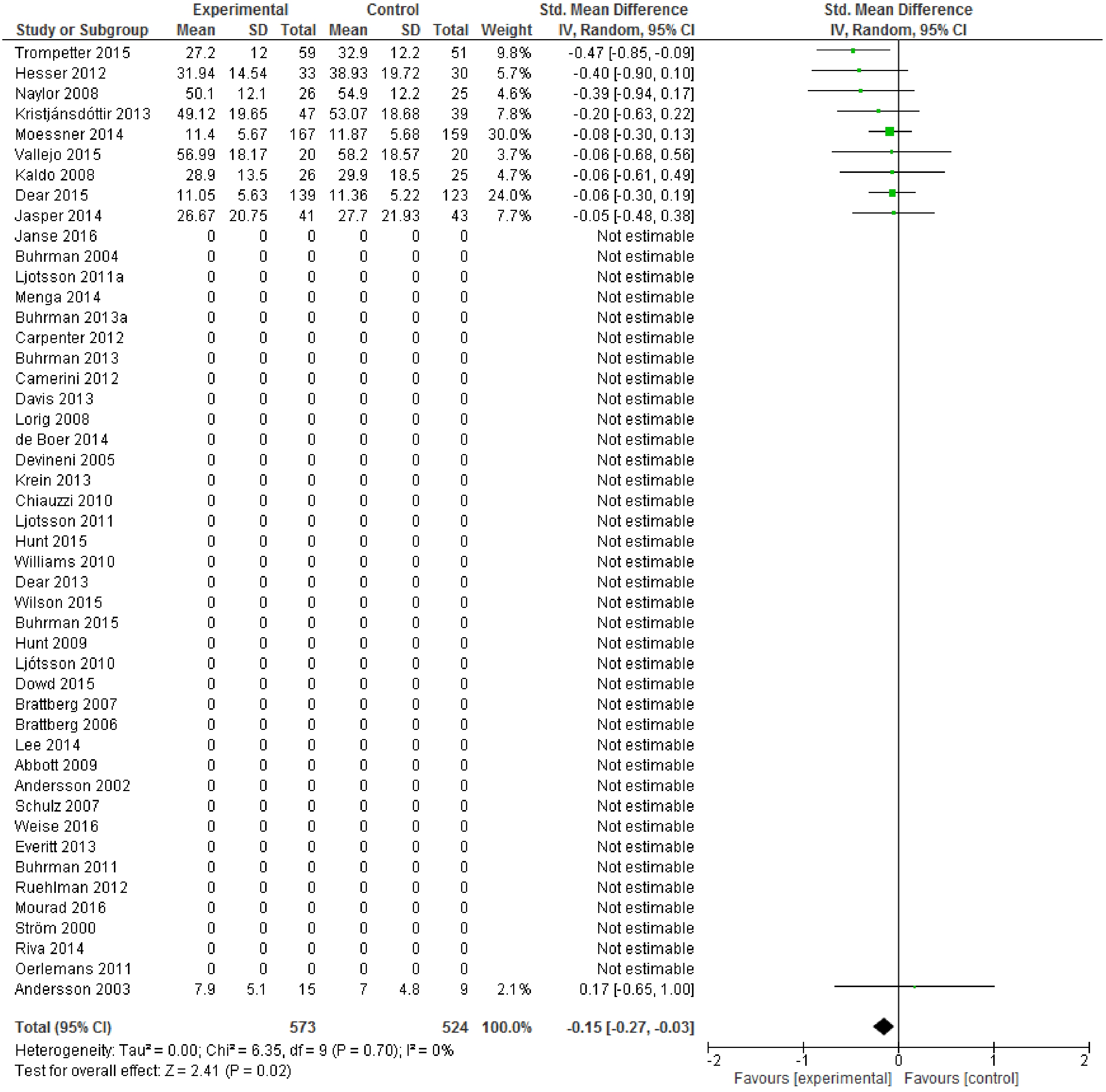

Figure T: Funnel plot

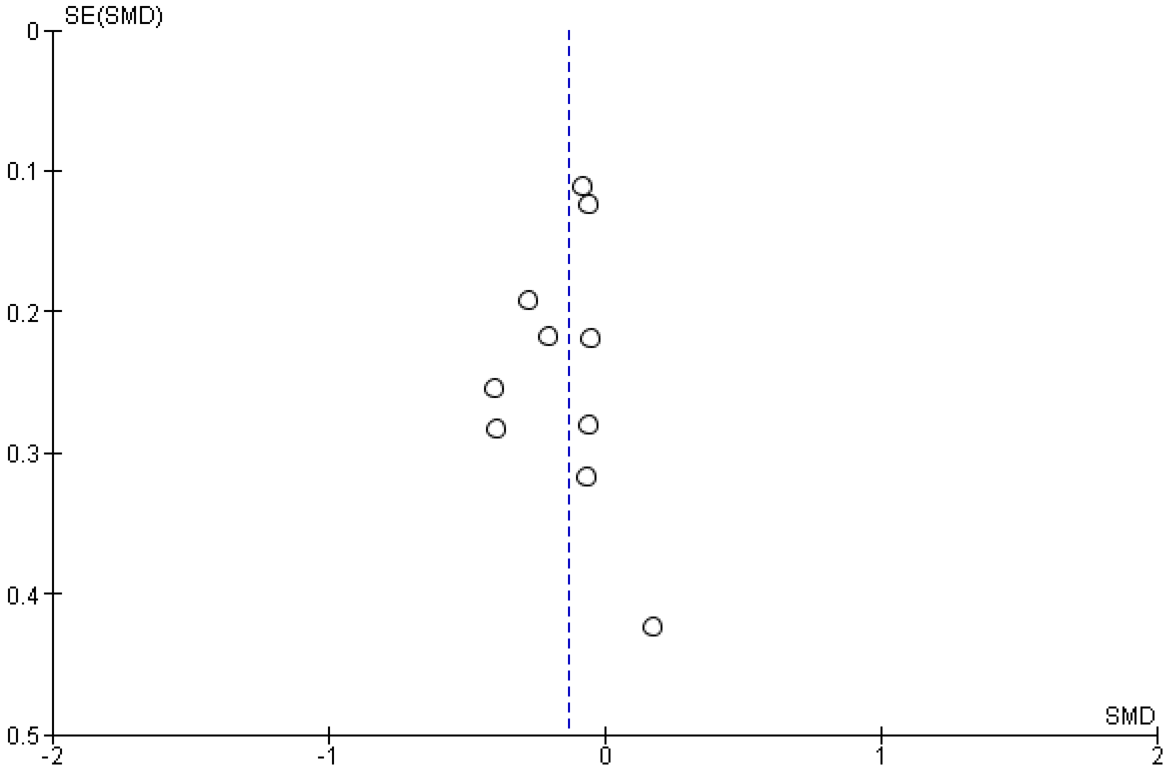

Outcome 2.4: Catastrophizing (post)
Number of eligible studies reporting the outcome: 10
Total number participants: 946

Table S

	SMD	**95% CI**	**I^2**	***P***
All eligible studies (k = 10)	-.26	[-.41, -.10]	21%	.25
SENSITIVITY ANALYSES				
**Internal validity**				
Low risk selection bias (k = 5)	-.33	[-.49, -.17]	5%	.38
Low risk attrition bias (k = 4)	-.33	[-.54, -.13]	0%	.61
Low risk reporting bias (k = 3)	-.16	[-.35, .02]	0%	.99
Low risk performance bias (k = 6)	-.21	[-.49, .06]	52%	.07
Low risk due to incomplete data extracted (k = 4)	-.13	[-.46, .20]	56%	.08
Low risk detection bias				
Low risk other bias (k = 7)	-.29	[-.42, -.15]	0%	.51
**External validity**				
Participants recruited from a general (open) population (k = 5)	-.19	[-.36, -.02]	0%	.93
Participants recruited from a clinical (closed) population (k = 3)	-.17	[-.83, .49]	72%	.03

SMD = Standardized mean difference, *CI* = Confidence interval, *P* = P-value for a Chi-square test for Tau; a measure of heterogeneity of standardized mean differences

Figure U: Forest plot all studies

Study or Subgroup	Experimental Mean	Experimental SD	Experimental Total	Control Mean	Control SD	Control Total	Weight	Std. Mean Difference IV, Random, 95% CI
Naylor 2008	6	9.4	25	11.2	7.2	25	6.3%	-0.61 [-1.18, -0.04]
Kristjánsdóttir 2013	-72.5	15.67	44	-63.55	13.33	38	9.5%	-0.61 [-1.05, -0.16]
Hesser 2012	-44.27	9.69	33	-40.47	8.86	30	7.9%	-0.40 [-0.90, 0.10]
de Boer 2014	12.55	11.53	20	17.13	12.49	24	5.7%	-0.37 [-0.97, 0.23]
Ljotsson 2011a	24.9	16.9	96	30.5	16.8	91	17.8%	-0.33 [-0.62, -0.04]
Trompetter 2015	13.5	11.3	59	15.6	11.7	51	12.4%	-0.18 [-0.56, 0.19]
Dear 2015	-26.79	6.69	139	-25.67	6.65	123	21.9%	-0.17 [-0.41, 0.08]
Jasper 2014	-47.91	11.7	41	-46.31	11.96	43	10.1%	-0.13 [-0.56, 0.29]
Andersson 2003	17.1	7.5	15	17.9	8.8	9	3.2%	-0.10 [-0.92, 0.73]
Williams 2010	0	0	0	0	0	0		Not estimable
Wilson 2015	0	0	0	0	0	0		Not estimable
Ström 2000	0	0	0	0	0	0		Not estimable
Weise 2016	0	0	0	0	0	0		Not estimable
Oerlemans 2011	0	0	0	0	0	0		Not estimable
Riva 2014	0	0	0	0	0	0		Not estimable
Ruehlman 2012	0	0	0	0	0	0		Not estimable
Schulz 2007	0	0	0	0	0	0		Not estimable
Menga 2014	0	0	0	0	0	0		Not estimable
Moessner 2014	0	0	0	0	0	0		Not estimable
Mourad 2016	0	0	0	0	0	0		Not estimable
Carpenter 2012	0	0	0	0	0	0		Not estimable
Chiauzzi 2010	0	0	0	0	0	0		Not estimable
Buhrman 2015	0	0	0	0	0	0		Not estimable
Camerini 2012	0	0	0	0	0	0		Not estimable
Dear 2013	0	0	0	0	0	0		Not estimable
Davis 2013	0	0	0	0	0	0		Not estimable
Brattberg 2006	0	0	0	0	0	0		Not estimable
Abbott 2009	0	0	0	0	0	0		Not estimable
Andersson 2002	0	0	0	0	0	0		Not estimable
Buhrman 2013	0	0	0	0	0	0		Not estimable
Buhrman 2013a	0	0	0	0	0	0		Not estimable
Buhrman 2004	0	0	0	0	0	0		Not estimable
Buhrman 2011	0	0	0	0	0	0		Not estimable
Lee 2014	0	0	0	0	0	0		Not estimable
Krein 2013	0	0	0	0	0	0		Not estimable
Kaldo 2008	0	0	0	0	0	0		Not estimable
Lorig 2008	0	0	0	0	0	0		Not estimable
Ljótsson 2010	0	0	0	0	0	0		Not estimable
Ljotsson 2011	0	0	0	0	0	0		Not estimable
Everitt 2013	0	0	0	0	0	0		Not estimable
Dowd 2015	0	0	0	0	0	0		Not estimable
Devineni 2005	0	0	0	0	0	0		Not estimable
Janse 2016	0	0	0	0	0	0		Not estimable
Hunt 2015	0	0	0	0	0	0		Not estimable
Hunt 2009	0	0	0	0	0	0		Not estimable
Vallejo 2015	24.63	5.11	20	20.9	8.7	20	5.2%	0.51 [-0.12, 1.14]
**Total (95% CI)**			**492**			**454**	**100.0%**	**-0.26 [-0.41, -0.10]**

Heterogeneity: $Tau^2 = 0.01$; $Chi^2 = 11.43$, df = 9 (P = 0.25); $I^2 = 21\%$
Test for overall effect: Z = 3.30 (P = 0.0010)

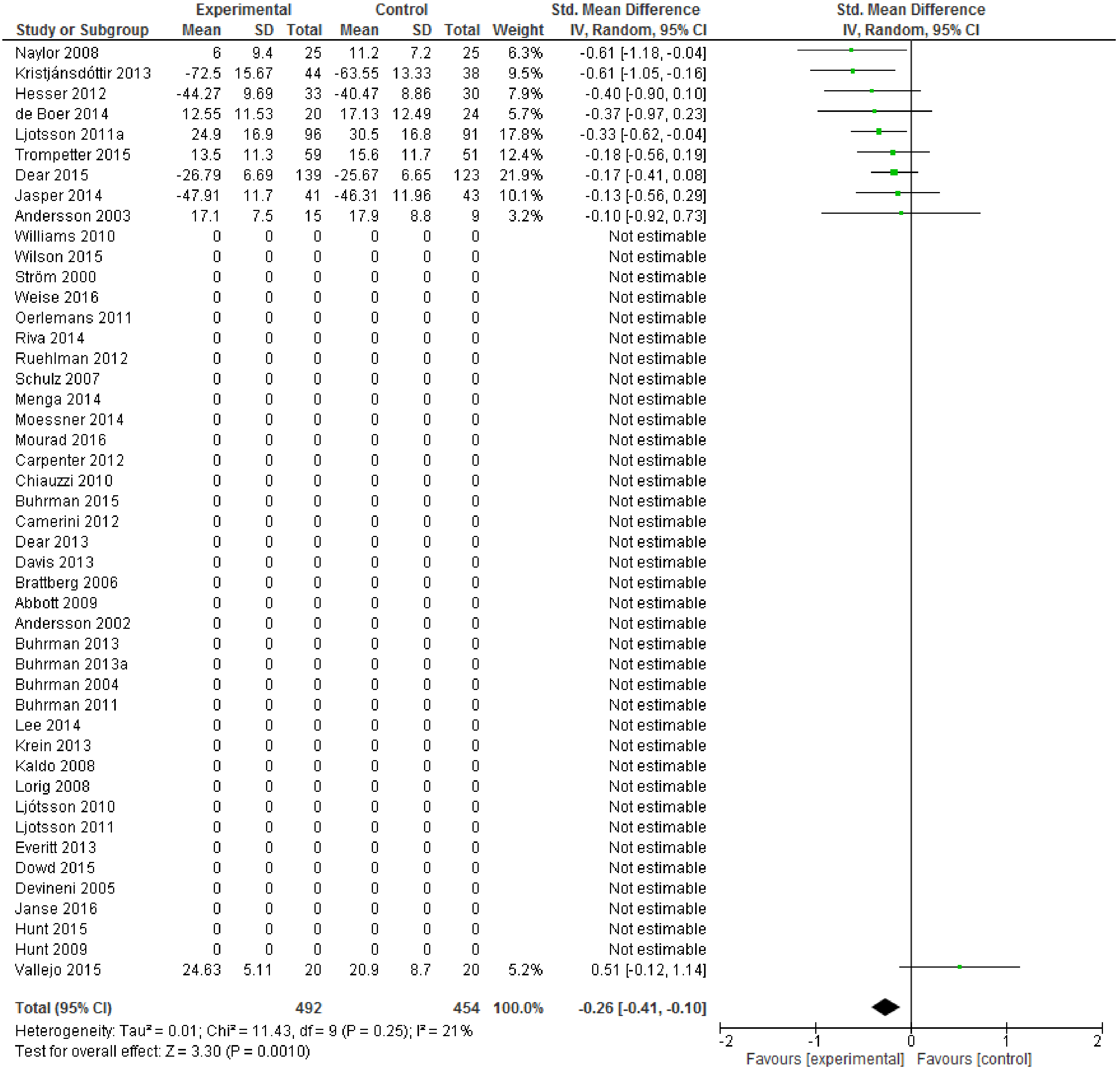

Figure V: Funnel plot

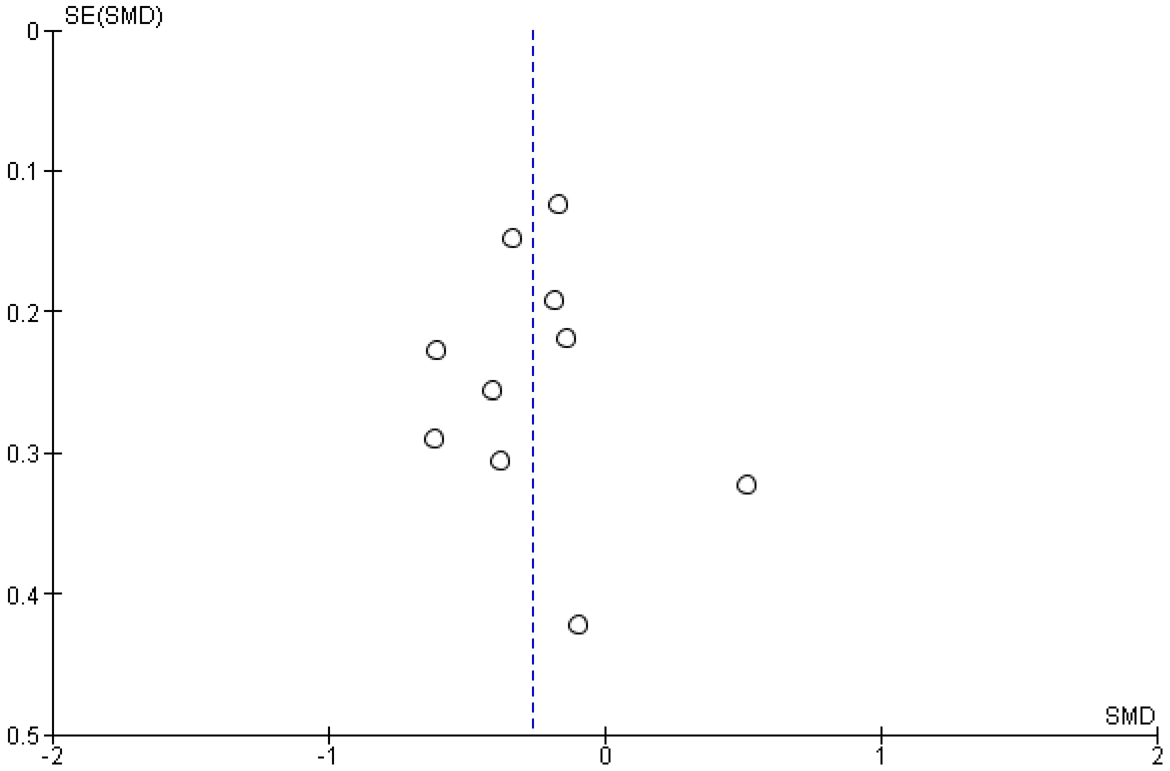

Outcome 2.5: Depression (post)
Number of eligible studies reporting the outcome: 8
Total number participants: 646

Table T

	SMD	95% CI	I^2	*P*
All eligible studies (k = 8)	-.14	[-.37, .09]	47%	.07
SENSITIVITY ANALYSES				
**Internal validity**				
Low risk selection bias (k = 3)	0	[-.22, .21]	0%	.98
Low risk attrition bias (k = 4)	0	[-.20, 20]	0%	.99
Low risk reporting bias (k = 2)	-.09	[-.37, .19]	0%	.58
Low risk performance bias (k = 6)	-.2	[-.55, .15]	60%	.03
Low risk due to incomplete data extracted (k = 2)	-.63	[-1.9, .66]	90%	.001
Low risk detection bias				
Low risk other bias (k = 5)	-.03	[-.2, .14]	0%	.96
**External validity**				
Participants recruited from a general (open) population (k = 4)	-.06	[-.3, .17]	0%	.92
Participants recruited from a clinical (closed) population (k = 1)	/	/	/	/

SMD = Standardized mean difference, *CI* = Confidence interval, *P* = P-value for a Chi-square test for Tau; a measure of heterogeneity of standardized mean differences

Figure W: Forest plot all studies

Study or Subgroup	Experimental Mean	Experimental SD	Experimental Total	Control Mean	Control SD	Control Total	Weight	Std. Mean Difference IV, Random, 95% CI
Vallejo 2015	11.32	3.33	20	16.38	4.15	20	7.8%	-1.32 [-2.01, -0.63]
Trompetter 2015	5.1	3.7	59	5.7	3.8	51	15.9%	-0.16 [-0.53, 0.22]
Andersson 2003	4.5	3.9	15	4.9	3.3	9	5.9%	-0.10 [-0.93, 0.72]
Kaldo 2008	4.3	3	26	4.5	3.2	25	10.6%	-0.06 [-0.61, 0.49]
Kristjánsdóttir 2013	1.78	2.51	46	1.86	2.07	37	13.9%	-0.03 [-0.47, 0.40]
Dear 2015	0	0	0	0	0	0		Not estimable
Jasper 2014	4.41	3.72	41	4.41	3.92	43	14.1%	0.00 [-0.43, 0.43]
Chiauzzi 2010	0	0	0	0	0	0		Not estimable
Camerini 2012	0	0	0	0	0	0		Not estimable
Williams 2010	0	0	0	0	0	0		Not estimable
Janse 2016	0	0	0	0	0	0		Not estimable
Dowd 2015	0	0	0	0	0	0		Not estimable
Hunt 2015	0	0	0	0	0	0		Not estimable
Buhrman 2004	0	0	0	0	0	0		Not estimable
Hunt 2009	0	0	0	0	0	0		Not estimable
Moessner 2014	0	0	0	0	0	0		Not estimable
Ljotsson 2011a	4.4	4.3	97	4.4	4	94	19.6%	0.00 [-0.28, 0.28]
Abbott 2009	0	0	0	0	0	0		Not estimable
Weise 2016	0	0	0	0	0	0		Not estimable
Carpenter 2012	0	0	0	0	0	0		Not estimable
Buhrman 2013a	0	0	0	0	0	0		Not estimable
Davis 2013	0	0	0	0	0	0		Not estimable
Devineni 2005	0	0	0	0	0	0		Not estimable
Andersson 2002	0	0	0	0	0	0		Not estimable
Dear 2013	0	0	0	0	0	0		Not estimable
Mourad 2016	0	0	0	0	0	0		Not estimable
Oerlemans 2011	0	0	0	0	0	0		Not estimable
Buhrman 2011	0	0	0	0	0	0		Not estimable
Lee 2014	0	0	0	0	0	0		Not estimable
Brattberg 2006	0	0	0	0	0	0		Not estimable
Krein 2013	0	0	0	0	0	0		Not estimable
Naylor 2008	0	0	0	0	0	0		Not estimable
Riva 2014	0	0	0	0	0	0		Not estimable
Ljótsson 2010	0	0	0	0	0	0		Not estimable
Lorig 2008	0	0	0	0	0	0		Not estimable
Buhrman 2013	0	0	0	0	0	0		Not estimable
Wilson 2015	0	0	0	0	0	0		Not estimable
Everitt 2013	0	0	0	0	0	0		Not estimable
Ruehlman 2012	0	0	0	0	0	0		Not estimable
Ljotsson 2011	0	0	0	0	0	0		Not estimable
Buhrman 2015	0	0	0	0	0	0		Not estimable
Menga 2014	0	0	0	0	0	0		Not estimable
de Boer 2014	0	0	0	0	0	0		Not estimable
Ström 2000	0	0	0	0	0	0		Not estimable
Schulz 2007	0	0	0	0	0	0		Not estimable
Hesser 2012	3.48	2.43	33	3.37	3.25	30	12.1%	0.04 [-0.46, 0.53]
**Total (95% CI)**			**337**			**309**	**100.0%**	**-0.14 [-0.37, 0.09]**

Heterogeneity: $Tau^2 = 0.05$; $Chi^2 = 13.14$, df = 7 ($P = 0.07$); $I^2 = 47\%$

Test for overall effect: $Z = 1.22$ ($P = 0.22$)

Std. Mean Difference

IV, Random, 95% CI

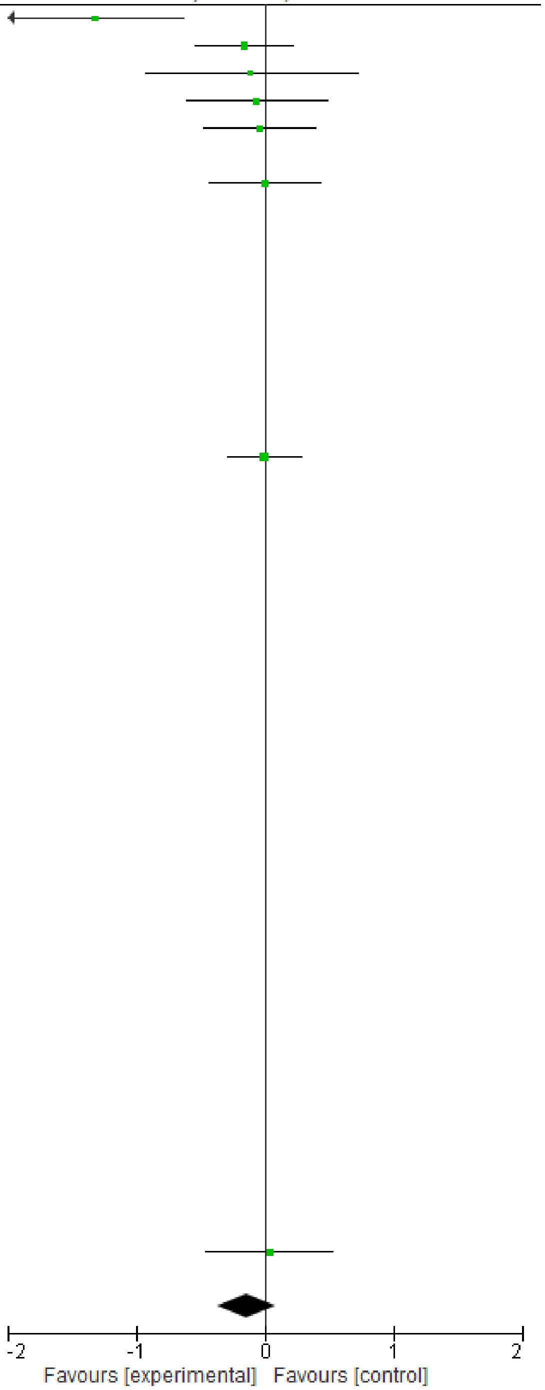

Figure X: Funnel plot

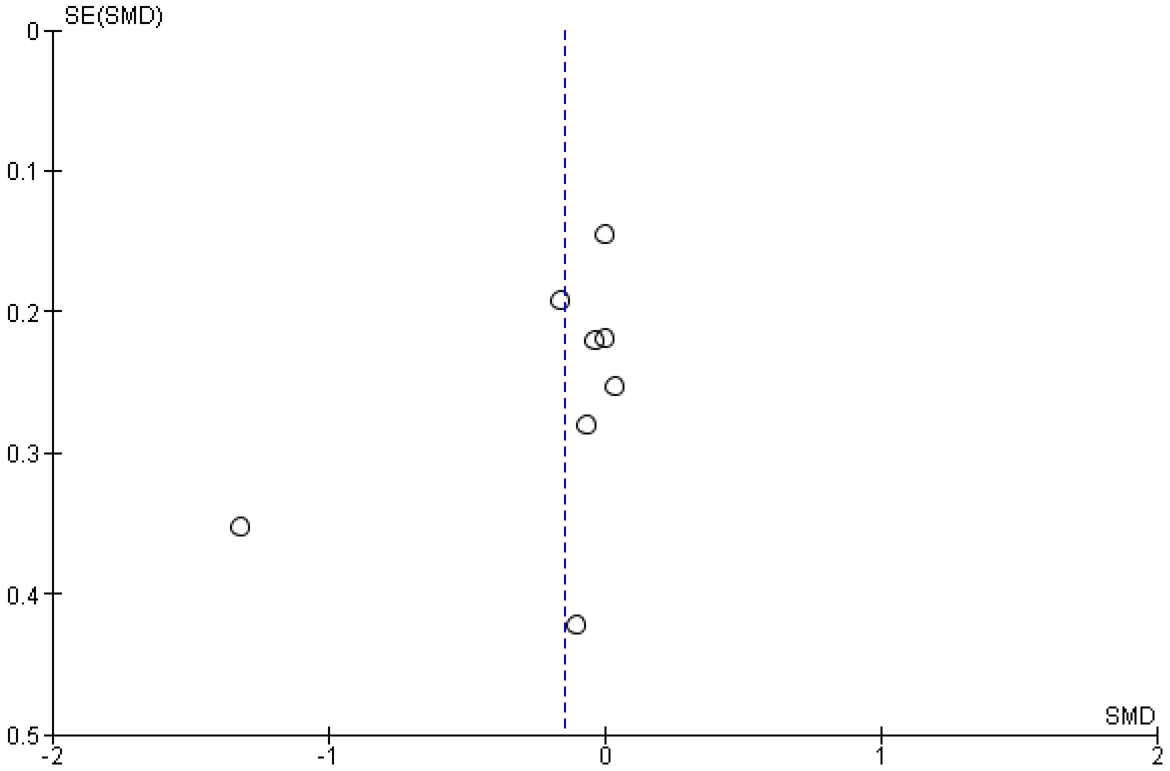

Outcome 2.6: Symptom intensity (6 or more months at follow-up)
Number of eligible studies reporting the outcome: 5
Total number participants: 683

Table U

	SMD	95% CI	I^2	*P*
All eligible studies (k = 5)	-.15	[-.40, .10]	60%	.04

SMD = Standardized mean difference, *CI* = Confidence interval, *P* = P-value for a Chi-square test for Tau; a measure of heterogeneity of standardized mean differences

Figure Y: Forest plot all studies

	Experimental			Control				Std. Mean Difference
Study or Subgroup	Mean	SD	Total	Mean	SD	Total	Weight	IV, Random, 95% CI
Ljotsson 2011a	33.4	13.4	87	39.3	13.3	82	22.5%	-0.44 [-0.75, -0.13]
Trompetter 2015	4.9	2.3	53	5.8	2.4	50	18.5%	-0.38 [-0.77, 0.01]
Jasper 2014	24.56	34.09	41	26.96	21.79	43	16.9%	-0.08 [-0.51, 0.34]
Hesser 2012	0	0	0	0	0	0		Not estimable
Dowd 2015	0	0	0	0	0	0		Not estimable
Chiauzzi 2010	0	0	0	0	0	0		Not estimable
Lorig 2008	0	0	0	0	0	0		Not estimable
Vallejo 2015	0	0	0	0	0	0		Not estimable
Naylor 2010	0	0	0	0	0	0		Not estimable
Brattberg 2007	0	0	0	0	0	0		Not estimable
Kristjánsdóttir 2013a	56.28	28.24	38	55.85	22.73	45	16.8%	0.02 [-0.42, 0.45]
Moessner 2014	4.33	2.32	128	4.03	2.54	116	25.3%	0.12 [-0.13, 0.37]
**Total (95% CI)**			**347**			**336**	**100.0%**	**-0.15 [-0.40, 0.10]**

Heterogeneity: Tau² = 0.05; Chi² = 9.92, df = 4 (P = 0.04); I² = 60%
Test for overall effect: Z = 1.19 (P = 0.24)

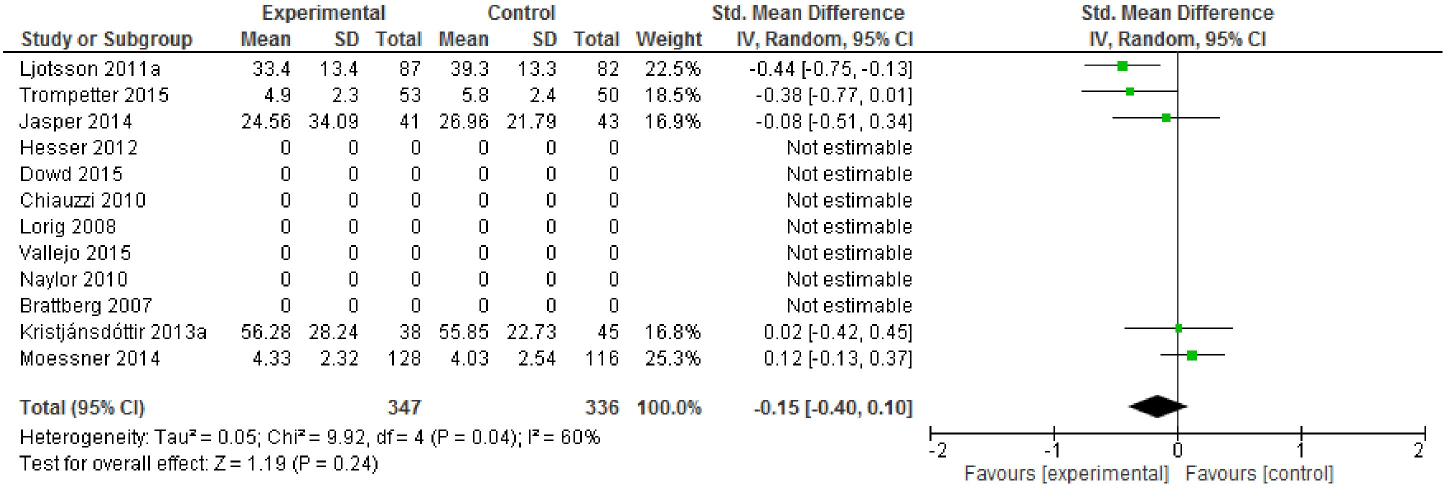

Outcome 2.7: HRQOL (6 or more months at follow-up)
Number of eligible studies reporting the outcome: 3
Total number participants: 461

Table V

	SMD	95% CI	I^2	*P*
All eligible studies (k = 3)	-.04	[-.37, .30]	66%	.05

SMD = Standardized mean difference, *CI* = Confidence interval, *P* = P-value for a Chi-square test for Tau; a measure of heterogeneity of standardized mean differences

Figure Z: Forest plot

	Experimental			Control				Std. Mean Difference
**Study or Subgroup**	**Mean**	**SD**	**Total**	**Mean**	**SD**	**Total**	**Weight**	**IV, Random, 95% CI**
Hesser 2012	-1.84	1.87	31	-2.48	1	30	23.9%	0.42 [-0.09, 0.93]
Ljotsson 2011	-74.9	20.8	87	-68.7	19	82	36.5%	-0.31 [-0.61, -0.01]
Moessner 2014	-42.28	10.54	122	-41.61	11.81	109	39.7%	-0.06 [-0.32, 0.20]
**Total (95% CI)**			**240**			**221**	**100.0%**	**-0.04 [-0.37, 0.30]**

Heterogeneity: Tau² = 0.06; Chi² = 5.93, df = 2 (P = 0.05); I² = 66%
Test for overall effect: Z = 0.21 (P = 0.83)

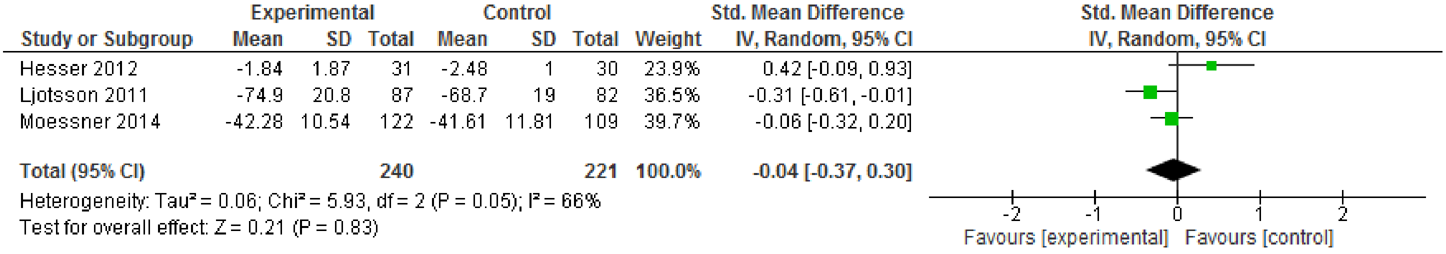

Outcome 2.8: Functional interference (6 or more months at follow-up)
Number of eligible studies reporting the outcome: 6
Total number participants: 672

Table W

	SMD	**95% CI**	**I^2**	***P***
All eligible studies (k = 5)	-.20	[-.44, .05]	56%	.05

SMD = Standardized mean difference, *CI* = Confidence interval, *P* = P-value for a Chi-square test for Tau; a measure of heterogeneity of standardized mean differences

Figure AA: Forrest plot

	Experimental			Control				Std. Mean Difference
**Study or Subgroup**	**Mean**	**SD**	**Total**	**Mean**	**SD**	**Total**	**Weight**	**IV, Random, 95% CI**
Vallejo 2015	51.52	15.25	20	65.14	17.91	20	9.9%	-0.80 [-1.45, -0.16]
Trompetter 2015	27.2	12	82	32.9	12.2	79	20.8%	-0.47 [-0.78, -0.16]
Kristjánsdóttir 2013a	49.24	21.34	38	53.75	17.73	45	15.9%	-0.23 [-0.66, 0.20]
Jasper 2014	24.56	34.09	41	26.96	21.79	43	16.1%	-0.08 [-0.51, 0.34]
Moessner 2014	11.1	6	128	10.99	6.79	115	23.7%	0.02 [-0.23, 0.27]
Hesser 2012	44.26	22.25	31	40.47	21.45	30	13.6%	0.17 [-0.33, 0.67]
**Total (95% CI)**			**340**			**332**	**100.0%**	**-0.20 [-0.44, 0.05]**

Heterogeneity: Tau² = 0.05; Chi² = 11.29, df = 5 (P = 0.05); I² = 56%
Test for overall effect: Z = 1.60 (P = 0.11)

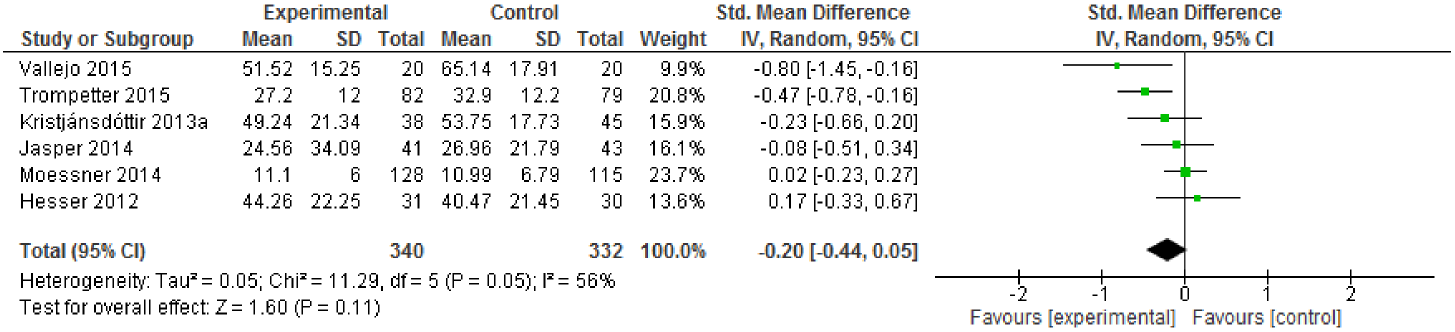

Outcome 2.9: Catastrophizing (6 or more months at follow-up)

Number of eligible studies reporting the outcome: 5

Total number participants: 432

Table X

	SMD	**95% CI**	**I^2**	***P***
All eligible studies (k = 5)	-.27	[-.56, .02]	53%	.08

SMD = Standardized mean difference, *CI* = Confidence interval, *P* = P-value for a Chi-square test for Tau; a measure of heterogeneity of standardized mean differences

Figure AB: Forest plot

	Experimental			Control				Std. Mean Difference
**Study or Subgroup**	**Mean**	**SD**	**Total**	**Mean**	**SD**	**Total**	**Weight**	**IV, Random, 95% CI**
Vallejo 2015	17.79	4.41	20	27.33	13.38	20	13.0%	-0.94 [-1.60, -0.28]
Ljotsson 2011a	23.1	16.8	85	29.4	17.5	81	27.2%	-0.37 [-0.67, -0.06]
Kristjánsdóttir 2013a	-71.62	14.11	39	-67.05	15.18	42	20.7%	-0.31 [-0.75, 0.13]
Jasper 2014	-49.14	12.04	41	-48.24	13.07	43	21.1%	-0.07 [-0.50, 0.36]
Hesser 2012	-37.1	8.11	31	-38.73	8.74	30	18.0%	0.19 [-0.31, 0.69]
**Total (95% CI)**			**216**			**216**	**100.0%**	**-0.27 [-0.56, 0.02]**

Heterogeneity: Tau² = 0.06; Chi² = 8.44, df = 4 (P = 0.08); I² = 53%

Test for overall effect: Z = 1.80 (P = 0.07)

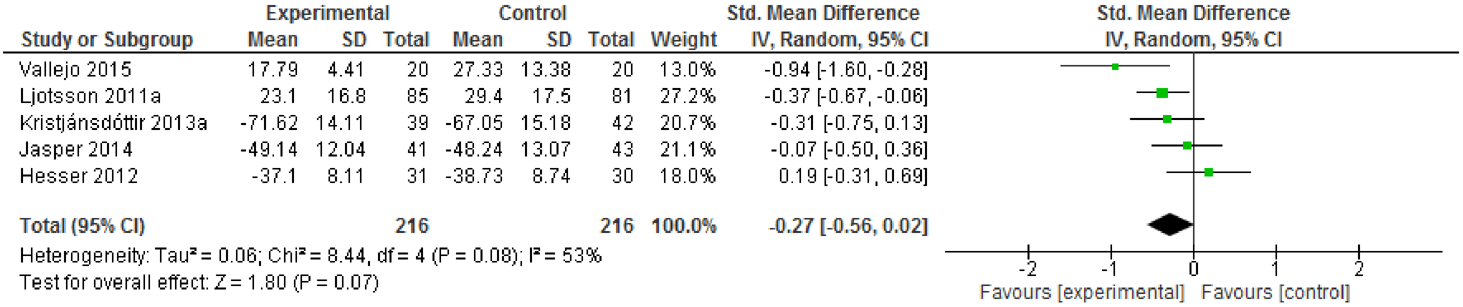

Outcome 2.10: Depression (6 or more months at follow-up)
Number of eligible studies reporting the outcome: 6
Total number participants: 517

Table Y

	SMD	95% CI	I^2	*P*
All eligible studies (k = 6)	-.31	[-.78, .16]	85%	<.001

SMD = Standardized mean difference, *CI* = Confidence interval, *P* = P-value for a Chi-square test for Tau; a measure of heterogeneity of standardized mean differences

Figure 29: Forest plot

	Experimental			Control				Std. Mean Difference
Study or Subgroup	Mean	SD	Total	Mean	SD	Total	Weight	IV, Random, 95% CI
Vallejo 2015	10.94	2.72	20	18.95	5.03	20	13.2%	-1.94 [-2.71, -1.18]
Trompetter 2015	4.3	3.8	53	6.2	3.9	50	17.8%	-0.49 [-0.88, -0.10]
Jasper 2014	4	4.3	31	4.96	4.74	30	16.5%	-0.21 [-0.71, 0.29]
Kristjánsdóttir 2013a	1.95	2.64	38	2.2	2.82	45	17.4%	-0.09 [-0.52, 0.34]
Ljotsson 2011a	4.4	4.1	87	4.7	3.9	82	18.8%	-0.07 [-0.38, 0.23]
Lorig 2008	0	0	0	0	0	0		Not estimable
Brattberg 2007	0	0	0	0	0	0		Not estimable
Dowd 2015	0	0	0	0	0	0		Not estimable
Naylor 2010	0	0	0	0	0	0		Not estimable
Chiauzzi 2010	0	0	0	0	0	0		Not estimable
Moessner 2014	0	0	0	0	0	0		Not estimable
Hesser 2012	5.03	3.36	31	3.07	2.95	30	16.4%	0.61 [0.10, 1.13]
**Total (95% CI)**			**260**			**257**	**100.0%**	**-0.31 [-0.78, 0.16]**

Heterogeneity: Tau² = 0.28; Chi² = 32.49, df = 5 (P < 0.00001); I² = 85%
Test for overall effect: Z = 1.29 (P = 0.20)

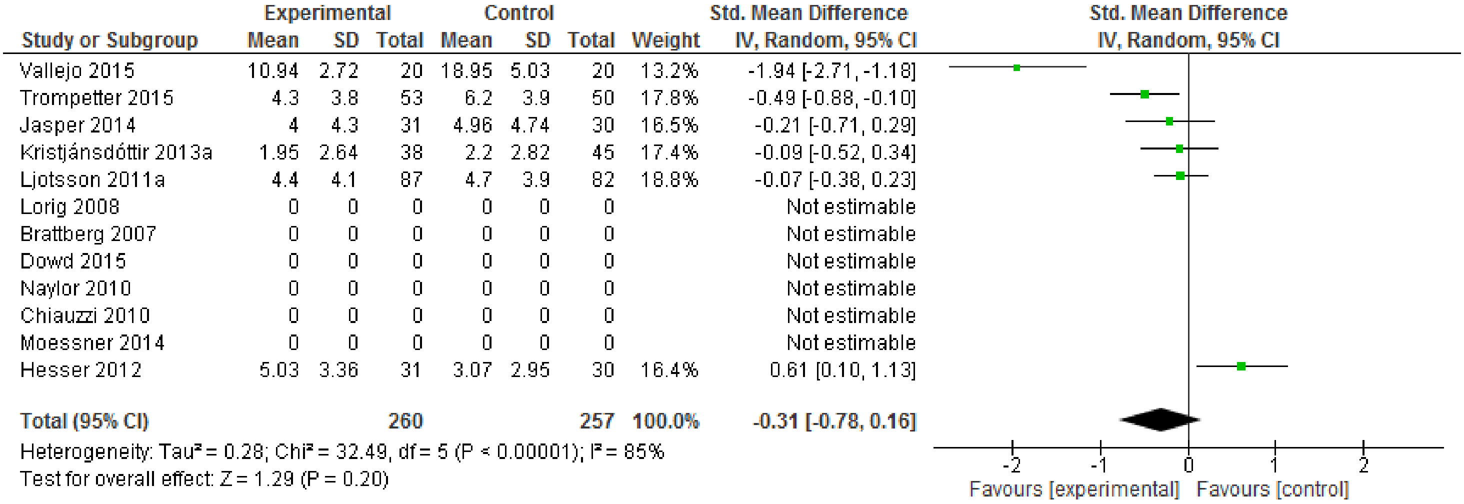

Table Z: Characteristics of sub-sets of studies with the 25% highest and 25% lowest SMD estimates

**Comparison category**	**CBI* vs. passive controls**							**CBI vs. active**		
**Outcome category**	**Any**	**Somatic symptoms**		**HRQOL[2]**		**Functional interference**		**Any**	**Somatic symptoms**	
Definition of study set	All	High 25%	Low 25%	High 25%	Low 25%	High 25%	Low 25%	All	High 25%	Low 25%
Size study set (k)	37	7	7	3	3	7	7	15	2	2
Type of control condition (k)[1]										
Wait-List	14	4	2	2	0	2	2	n.a.	n.a.	n.a.
Usual/standard care	9	1	2	0	1	0	3	n.a.	n.a.	n.a.
Message board	8	2	1	1	0	4	2	n.a.	n.a.	n.a.
Information	6	0	2	0	2	1	0	n.a.	n.a.	n.a.
Other CBI version	n.a.*	n.a.	n.a.	n.a.	n.a.	n.a.	n.a.	8	1	1
No CBI component	n.a.	n.a.	n.a.	n.a.	n.a.	n.a.	n.a.	3	1	1
Face-to-face group therapy	n.a.	n.a.	n.a.	n.a.	n.a.	n.a.	n.a.	4	0	0
Intervention										
Treatment duration in weeks (mean, *SD*)	10.43, *9.54*	9.86, *7.49*	6.71[3], *0.95*	7.00, *2.65*	6.67, *1.16*	10.00, *5.86*	9.14, *7.56*	10.87, *6.37*	21.50, *6.36*	6.00, *2.83*
Theory/model mentioned (k)[4]										
Traditional CBT* model	14	3	3	2	2	2	2	4	0	0
Traditional CBT with other model(s)	3	0	0	0	0	1	0	3	1	0
"Third wave" of CBT approach	5	1	1	1	0	2	1	4	1	1
Traditional CBT inspired by "third wave"	6	1	1	0	0	1	0	0	0	0
No reference, author constructed, or other	9	2	2	0	1	1	4	4	0	1
Theory used to select techniques (item 5)	17	5	4	3	0	2	2	5	2	1
Explicit link between targeted construct and intervention (items 7-11)	13	5	3	3	1	2	3	7	2	1
Behavioral change techniques (k)										
Antecedents	26	4	7	2	2	6	4	1	0	0
Association	12	4	1	3	1	4	1	2	0	0
Comparison of outcome	24	4	5	2	2	5	2	6	1	1
Feedback and monitoring	23	6	6	2	3	5	4	3	1	1
Goals and planning	24	3	5	1	2	6	3	2	0	1
Identity	28	6	7	3	2	6	4	4	1	1
Natural consequences	30	7	7	3	3	6	5	1	0	0
Regulation	31	5	7	3	2	5	6	2	1	1
Repetition and substitution	33	6	7	3	2	6	5	6	2	1
Social support	26	4	5	2	2	6	2	4	0	2
Total amount of techniques (mean, *SD*)	11.92, *4.12*	11.14, *3.85*	14.43, *1.40*	13.00, *1.00*	11.67, *7.51*	13.29, *5.27*	9.71, *5.02*	3.13, *3.40*	5.00, *2.83*	8.50[5], *6.36*
Delivery modes										
Automated functions 0-3 (mean, *SD*)	2.11, *0.77*	1.71, *0.49*	2.29, *0.76*	1.67, *0.58*	2.00, *0.00*	2.14, *0.90*	2.43, *0.53*	1.07, *1.03*	1.00, *0.00*	1.50, *0.71*
Communicative functions 0-3 (mean, *SD*)	0.84, *0.65*	0.86, *0.90*	1.00, 0.00	1.00, *1.00*	0.67, *0.58*	1.00, *0.58*	0.57, *0.53*	0.73, *0.70*	0.50, *0.71*	0.00, *0.00*
Supplementary modes 0-5 (mean, *SD*)	2.13, *0.75*	1.86, *0.38*	2.43, *0.53*	2.00, *0.00*	2.00, *0.00*	2.29, *0.76*	2.29, *0.49*	1.13, *0.99*	0.50, *0.71*	1.50, *2.12*
Total amount of MODs* 0-11 (mean, *SD*)	5.08, *1.34*	*4.43*[6], *1.27*	5.71, *1.25*	4.67, *1.53*	4.67, *0.58*	5.43, *1.62*	5.29, *1.25*	2.93, *1.98*	2.00, 0.00	3.00[6], *2.83*
Provider contact (k)										
No provider contact	13	3	2	1	1	1	5	2	0	1
No psychologist	4	0	1	0	1	0	0	0	0	0

Masters level psychologist	8	1	1	1	1	2	2	7	2	0
Clinical training	11	2	3	0	0	4	0	5	0	1
Unclear expertise	1	1	0	1	0	0	0	1	0	0
Completers intervention group (mean proportion, *SD*)	0.59, *0.25*	0.52, *0.14*	0.34[7], *0.12*	0.58, *0.16*	0.28, *0.06*	0.74, *0.26*	0.39, *0.16*	0.63, *0.24*	0.58, *0.15*	0.70, /
Patients										
Participant age (mean, *SD*)	45.25, *5.53*	38.39[8], *5.14*	47.91, *2.68*	36.53, *2.25*	47.40, *2.71*	48.67, *1.28*	46.79, 5.12	47.39, *4.16*	49.40, *4.81*	45.75, *2.19*
Female (mean proportion, *SD*)	0.70, *0.23*	0.82[9], *0.10*	0.57, *0.26*	0.82, *0.03*	0.61, *0.50*	0.70, *0.19*	0.65, *0.30*	0.72, *0.20*	0.80, *0.06*	0.75, *0.35*
Completed tertiary education (mean proportion, *SD*)	0.41, *0.14*	0.54[10], *0.08*	0.28, *0.09*	0.51, *0.01*	0.41, *0.10*	0.50, *0.09*	0.36, *0.16*	0.47, *0.18*	0.70, /	0.31, *0.11*
Participants employed (mean proportion, *SD*)	0.65, *0.21*	0.64, *0.22*	0.74, *0.37*	0.48 /	1.00 /	0.70, *0.21*	0.68, *0.29*	0.66, *0.12*	0.40, /	0.72, /
Participants on sick leave (mean proportion, *SD*)	0.38, *0.27*	/	0.42, *0.36*	/	/	0.54, *0.44*	0.27, *0.07*	0.25, *0.18*	/	0.55, /
Complaint duration in months (mean, *SD*)	110.73, *29.65*	93.00[11], *39.09*	127.16, *40.08*	75.60, /	116.84, *17.93*	125.37, *45.78*	104.17, /	122.57, *28.29*	133.80, /	136.94, *48.41*
HADS* depression (mean proportion, *SD*)	6.95, *1.38*	/	6.70, *2.02*	/	4.90 /	8.08, *1.47*	6.2 /	5.83[12], *0.52*	6.20, /	/
Patient condition (k)										
Chronic pain	13	0	3	0	0	4	3	4	2	0
Chronic (low) back pain	4	0	1	0	0	0	1	2	0	1
Fibromyalgia/ chronic widespread pain	4	0	0	0	0	1	0	3	0	1
Headache	2	1	0	0	0	0	1	1	0	0
Chronic fatigue	1	1	0	0	0	0	0	0	0	0
Irritable Bowel Syndrome	6	4	1	3	1	0	0	2	0	0
Interstitial cystitis	1	1	0	0	1	0	1	0	0	0
Non-cardiac chest pain	1	0	0	0	0	0	0	0	0	0
Tinnitus	5	0	2	0	1	2	1	3	0	0
Computer literacy selection criteria (k)										
Implicit	7	4[13]	1	3	1	0	0	3	0	1
Explicit (able to use required technology)	27	1	6	0	1	7	6	11	2	0
Requires other platform or run-in period	3	2	0	0	1	0	1	1	0	1

Comment: table only includes outcomes reported in 10 or more studies, and for which heterogeneity in pooled SMDs was statistically significant and I^2 more than 40%.

*Abbreviations: CBI = Computer-based intervention, HRQOL = Health-related quality of life, N.A. = Not applicable, / = no data, CBT = Cognitive Behavioral Therapy, BCT = Behavioral Change Technique, MOD = Mode of Delivery, HADS = Hospital Anxiety and Depression Scale.

[1] Sub-groups for type of control were made in accordance with the categories in the table.

[2] Two markedly small studies were in the set of studies with high post-treatment SMDs for HRQOL (k = 3). Replacement of these studies by two other studies with relatively high SMDs did not affect the selection of potentially distinctive characteristics for sub-group analysis.

[3] Treatment duration: subsequent sub-group analyses distinguished between studies with a duration of up to 6 weeks, 7-10 weeks, or more than 10 weeks.

[4] Sub-groups for "use of theory" are: no CBT model, traditional CBT, 3rd wave model (Mindfulness-based or Acceptance and commitment therapy), or 3rd wave inspired.

[5] Number of BCTs sub-groups are: 0 or unclear, 1-3, more than 3

[6] Number of MODs sub-groups are: (for intervention versus passive controls) 0-4, 5, or more than 5, and (for intervention versus active controls) 0-2, or more than 2. Number of automated, communicative, supplementary modes groups are: 0, 1, and more than 1.

[7] Compliance: 50% of intervention group treatment completers was the cut-off point used for sub-group analyses.

[8] Average participant age groups are: up to 42.5, between 42.5 and 49, or more than 49 years of age.

[9] Average female proportion groups are: less than 2/3, between 2/3 and 4/5, and more than 4/5.

[10] Average proportion of participants with tertiary education groups are: up to 40%, and more than 40%.

[11] Symptom duration groups are: up to 100 months on average, and more than 100 months on average.

[12] Average (baseline) HADS scores were categorized as: up to 7 (probably not depressed), or higher than 7 (depression is probable)

[13] Computer literacy selection criteria groups are: explicit vs. other.

Table AA: Overview of sub-group analyses

		CBI vs. active			CBI vs. active
**Outcome category**	**Sub-group definition**	**Somatic symptoms**	**HRQOL[2]**	**Functional interference**	**Somatic symptoms**
Type of control condition within passive controls	- Waiting list<br>- Usual/standard care<br>- Message board<br>- Information<br>- Other CBI version<br>- No CBI component<br>- Face-to-face group therapy	Chi² = 12.79, (P = 0.005), I² = 76.5%	Chi² = 19.37, (P = 0.0002), I² = 84.5%	Chi² = 22.73, (P < 0.0001), I² = 86.8%	
<u>Intervention</u>					
Treatment duration in weeks (mean, *SD*)	- <= 6 weeks<br>- 7-10 weeks<br>- >10 weeks	Chi² = 1.29, (P = 0.52), I² = 0%		Chi² = 5.51, (P = 0.06), I² = 63.7%	Chi² = 2.08 (P = 0.35), I² = 3.6%
Theory/model mentioned	- Traditional CBT* model<br>- Traditional CBT with other model(s)<br>- “Third wave” of CBT approach<br>- Traditional CBT inspired by “third wave”<br>- No reference, author constructed, or other		Chi² = 8.10, (P = 0.04), I² = 63.0%	Chi² = 1.45, (P = 0.69), I² = 0%	
Theory used to select techniques (item 5)			Chi² = 5.79, (P = 0.02), I² = 82.7%		
Explicit link between targeted construct and intervention (items 7-11)			Chi² = 1.90, (P = 0.17), I² = 47.5%		
Antecedents		Chi² = 1.68, (P = 0.19), I² = 40.6%		Chi² = 1.13, (P = 0.29), I² = 11.5%	
Association (i.e. 7.7 exposure)	- Present<br>- Not present	Chi² = 6.26, (P = 0.01), I² = 84.0%	Chi² = 2.24, (P = 0.13), I² = 55.4%	Chi² = 3.72, (P = 0.05), I² = 73.1%	
Comparison of outcome				Chi² = 1.99, (P = 0.16), I² = 49.7%	
Goals and planning				Chi² = 0.95, (P = 0.33), I² = 0%	
Identity				Chi² = 3.60, (P = 0.06), I² = 72.3%	
Repetition and substitution					Chi² = 0.59, (P = 0.44), I² = 0%
Social support				Chi² = 3.62, (P = 0.06), I² = 72.4%	Chi² = 1.76, (P = 0.18), I² = 43.3%
Total amount of techniques (versus active controls)	- 0 or unclear<br>- 1-3<br>- > 3				Chi² = 0.97 (P = 0.62), I² = 0%
Automated functions	- *0*<br>- *1*<br>- *> 1*				Chi² = 0.86, (P = 0.35), I² = 0%
Communicative functions	- *0*<br>- *1*<br>- *> 1*		Chi² = 2.87, (P = 0.24), I² = 30.4%		
Supplementary modes	- *0*<br>- *1*				Chi² = 2.13

	- *> 1*				(P = 0.34),<br>I^2 = 6.2%
Peer-to-peer access	- *Present*<br>- *Not present*		Chi^2 = 1.96,<br>(P = 0.16),<br>I^2 = 48.9%		
Total amount of MODs (versus passive controls)	- 0-4<br>- 5<br>- > 5	Chi^2 = 6.34,<br>(P = 0.04),<br>I^2 = 68.4%			
Total amount of MODs (versus active controls)	- 0-2<br>- > 2				Chi^2 = 5.11,<br>(P = 0.02),<br>I^2 = 80.4%
Provider presence and training level	- No psychologist<br>- Other<br>- Masters level psychologist<br>- Clinical training			Chi^2 = 9.84,<br>(P = 0.02),<br>I^2 = 69.5%	
Completers intervention group	- < 50%<br>- =< 50%	Chi^2 = 4.55,<br>(P = 0.03)<br>I^2 = 78.0%		Chi^2 = 2.30,<br>(P = 0.13),<br>I^2 = 56.6%	
Participant age (years)	- *< 42.5*<br>- *=> 42.5 & =< 49*<br>- *>49*	Chi^2 = 11.45,<br>(P = 0.003),<br>I^2 = 82.5%	Chi^2 = 15.11,<br>(P = 0.0001),<br>I^2 = 93.4%		
Female (mean proportion, *SD*)	- *< 2/3*<br>- *=> 2/3 & =< 4/5*<br>- *>4/5*	Chi^2 = 9.19,<br>(P = 0.01), I^2 = 78.2%	Chi^2 = 4.30,<br>(P = 0.12),<br>I^2 = 53.5%		
Completed tertiary education	- *=<40%*<br>- *>40%*	Chi^2 = 5.46<br>(P = 0.02), I^2 = 81.7%	*>10*		
Participants on sick leave	- *< 50%*<br>- *=> 50%*			Chi^2 = 3.73,<br>(P = 0.05),<br>I^2 = 73.2%	
Complaint duration in months	- < 100 months<br>- 100 months or more	Chi^2 = 2.13,<br>(P = 0.14),<br>I^2 = 53.0%			
HADS* depression	- *=< 7*<br>- *>7*			Chi^2 = 5.32,<br>(P = 0.02), I^2 = 81.2%	
Patient condition	- Chronic pain<br>- Chronic (low) back pain<br>- Fibromyalgia/ chronic widespread pain<br>- Headache<br>- Chronic fatigue<br>- Irritable Bowel Syndrome<br>- Interstitial cystitis<br>- Non-cardiac chest pain<br>- Tinnitus	Chi^2 = 15.62,<br>(P = 0.03),<br>I^2 = 55.2%	Chi^2 = 8.36,<br>(P = 0.08),<br>I^2 = 52.2%		
Computer literacy selection criteria	- Explicit<br>- Other (able to use required technology / other platform / run-in period?	Chi^2 = 4.79,<br>(P = 0.03),<br>I^2 = 79.1%			

Symbols and abbreviations: CBI = Computer-based Intervention, HRQOL = Health Related Quality of Life, Chi^2 = Chi-Square test statistic, P = P-Value, I^2 = Heterogeneity statistic, CBT = Cognitive Behavioural Therapy
