# Supplementary material for: The effectiveness of various computer-based interventions for patients with chronic pain or functional somatic syndromes: A systematic review and meta-analysis
Source: PLoS One. 2018 May 16;13(5):e0196467. doi: 10.1371/journal.pone.0196467 (PMC5955495; doi:10.1371/journal.pone.0196467)
Supplement: S1 Protocol — (PDF) [file pone.0196467.s003.pdf]

UNIVERSITY of York
Centre for Reviews and Dissemination

NHS
National Institute for
Health Research

PROSPERO International prospective register of systematic reviews

Which computer-based behavioral interventions reduce symptoms in which patients with chronic pain or functional somatic syndromes?

Miel Vugts, Margot Joosen, Aglaia Zedlitz, Jessica van der Geer, Hubertus Vrijhoef

Citation

Miel Vugts, Margot Joosen, Aglaia Zedlitz, Jessica van der Geer, Hubertus Vrijhoef. Which computer-based behavioral interventions reduce symptoms in which patients with chronic pain or functional somatic syndromes?. PROSPERO 2016:CRD42016050839 Available from
http://www.crd.york.ac.uk/PROSPERO_REBRANDING/display_record.asp?ID=CRD42016050839

Review question(s)

Which computer-based interventions are effective for which patients with chronic pain (CP) or functional somatic syndromes (FSS)?

Sub-question 1: What are effective computer-based interventions for health outcomes in patients with CP or FSS compared to wait-list conditions, usual care or standard care?

Sub-question 2: What are effective computer-based interventions for patient health outcomes compared to other active treatment conditions?

Sub-question 3: What are the characteristics of patients for whom computer-based interventions are most/least effective?

Differences in effect size between studies will be weighed after the considering methodological factors that may affect the internal (risk of bias) and external validity (i.e. participant selection methods).

Sub-question 4: What are the characteristics (duration, theoretical approach, behavioral change techniques, and delivery modes) of the most/least effective computer-based interventions?

Searches

The following electronic bibliographic databases were searched (in June 2016): MEDLINE, EMBASE, PsycINFO, the Cochrane Central Register of Controlled Trials (CENTRAL), and Web of Science (Science and Social Science Citation Index). PubMed was also searched.

The full search strategy, as used for our search in EMBASE, is available as an attachment. The strategy combines search terms that were copied from previously published Cochrane reviews relating to or describing the population (Functional Somatic Syndromes), (and) intervention (computer-based and behavioral intervention), and study type. Adapted search strategies were used with other bibliographic databases, depending on the interface available (OVID, EBSCOhost).

Just before the study inclusion date (June 2016), we sought studies that had been published since January 1990 (without language restrictions).

Finally, citation networks of eligible studies (forward and backward citations) were checked.

Types of study to be included

Included are randomized controlled trials (RCTs), quasi-experimental studies, or mixed-methods studies published in peer-reviewed scientific journals.

Studies are excluded that are not empirical evaluations, only make use of qualitative data, have no control group, or are not published as peer reviewed journal articles.

Condition or domain being studied
Chronic pain (CP): Patients reporting (specific or non-specific) pain persisting beyond a usual 3 to 6 month duration of organic recovery.

Functional somatic syndromes (FSS): Individuals seeking medical help for functional disturbance and chronic somatic symptoms without a satisfactory explanation by organ pathology or disease.

Participants/ population
Inclusion criteria: Adults, CP or FSS condition (this also includes 'somatoform or somatic symptom disorder' (DSM/ICD), 'medically unexplained physical symptoms' (MUPS), identified either by clinician assessment or case-finding using validated diagnostic instruments.

Exclusion criteria: Condition is not chronic (<3 months duration), primary prevention, pediatric or geriatric populations, all patients in the study are diagnosed with a specific organic disease (i.e. migraine, multiple sclerosis, osteoarthritis).

Intervention(s), exposure(s)
Inclusion criteria: Computer-based interventions, which are programs used by patients via computer technology (the use of a computer is required to follow the program), i.e. the program uses the internet as a delivery route, interactive television, interactive voice response, mobile telephone/smartphone, CD-ROM/DVD and handheld computers. The program includes (a) feature(s) that (is) are directly available to users. The program is directed at self-management, education or psychological treatment, and/or health behavior change. More specifically, the program should include the provision of information and at least some sort of behavioral change support (i.e. skills training, cognitive behavior therapy, self-monitoring, feedback, stress-management, biofeedback, counselling, hypnotherapy, conditioning, etc.).

Exclusion criteria: The program does not target patients themselves, is designed to be used exclusively with professional assistance, participants are passive recipients, or if the intervention only provides means for (distant) communication with care providers.

Comparator(s)/ control
Any comparison is eligible.

Context
No other criteria are relevant to this review.

Outcome(s)
Primary outcomes
Mean difference in symptom severity. Different instruments may be used across studies. If multiple measures are available within one included study, more general measures (visual analogue or numerical rating scales for pain or fatigue intensity) are preferred over specific measures (i.e. tinnitus, abdominal symptoms, headache).

Timing: Post-treatment (regardless of program duration).

Mean difference in change scores will be used instead of post-treatment mean difference if there are severe distributional issues.

Secondary outcomes
Mean difference in symptom severity at follow-up (more than 6 months after baseline).

Post-treatment mean difference of:

Symptomatic interference (i.e. validated measures of disability or disease impact);

Coping and cognition (i.e. validated measures of catastrophizing, acceptance, fear avoidance beliefs, self-efficacy);

Depression (preferably measured with the Hospital Anxiety and Depression Scale);

Health-related quality of life (preferably measured with the SF-36);

Treatment adherence (measured as the number of intervention participants completing the program).

For follow-up measures: the last observations will be used.

Mean difference in change scores will be used instead of post-treatment mean difference if there are problematic distributional issues.

Data extraction, (selection and coding)

Half of the study titles and/or abstracts were screened independently in order to identify potentially eligible articles. After three iterations of sub-sample screenings, three authors (MV, MJ, & HV) aligned the identifications of potentially eligible study types, patient conditions, and interventions. The final half of the titles and/or abstracts were screened by MV. Full-text eligibility assessment of the remaining studies was performed independently by MV & MJ, and discrepancies were resolved by discussions involving HV.

A data extraction form was created, discussed, and piloted before use. This form consisted of standard items; study year, design, recruitment, comparisons, dose and duration, outcome measures, participant flow, and missing data handling. Emphasis was placed on extracting characteristics of patient participants, interventions and external validity information. Patient items include demographics (mean age, proportion of female participants, proportion of respondents with secondary and tertiary education), diagnosis, and baseline health conditions (somatic symptoms, functional interference, and depression). Existing taxonomies are used to categorize the theoretical frameworks, behavioral change techniques, and delivery modes for the interventions. Relevant items for information on external validity were taken form the Ehealth-CONSORT statement (human involvement, prompts/reminders used, co-interventions, and author comments on external validity). The intervention characteristics were extracted independently by two authors who are trained for applying the Behavioral Change Taxonomy v1 (MV & JG). The remaining items were extracted by MV, and checked by AZ. Data were extracted from the included studies or from published studies that authors refer to for particular information.

Risk of bias (quality) assessment

Independent quality assessment was performed by two authors (MV, JG), using the risk of bias criteria of the Cochrane Low Back Pain group, as follows:

Selection bias due to: (1) inadequate generation of a randomized sequence; (2) inadequate concealment of allocations prior to assignment; or (8) dissimilarity at baseline for the most important prognostic indicators.

Performance bias due to knowledge of the allocated interventions by (3) participants or (4) personnel/care-providers during the study, (9) because co-interventions were different across groups, or (10) due to inappropriate compliance with interventions across groups.

Detection bias (5) due to knowledge of the allocated interventions by outcome assessors, or (12) because important outcomes were not measured at the same time across groups.

Attrition bias (6) due to amount, nature or handling of incomplete outcome data.

Reporting bias (7) due to selective outcome reporting.

Bias (11) due to incomplete reporting and analysis according to group allocation.

Bias (13) due to problems not covered elsewhere.

HV was involved in discussions with quality assessors to resolve discrepancies and to explicate the use of the criteria objectively and consistently.

Strategy for data synthesis

Data will be synthesized as follows:

Firstly, by providing a description of:

- Variation in the characteristics of computer-based interventions for patients with CP or FSS in the published peer reviewed scientific literature.

- Variation in the characteristics of patients who participated in the included studies.

- Variation in risk of bias.

- Variation in items relevant for externalizing the results to patients treated in regular outpatient clinics.

Secondly, chi-square tests will be run to explore associations between various sources of variation between studies.

Thirdly, meta-analysis software of the Cochrane collaboration (RevMan 5.3) will be used to pool the results for two sorts of comparisons: 1) computer-based vs. passive (control group described as 'wait-list', 'care as usual', or 'standard care'), or 2) computer-based vs. active (i.e. the control group receives a similar active intervention face-to-face, another version of a computer-based intervention, or active face-to-face treatment without additional computer-based component). RevMan operations will be set for random-effects meta-analyses with weighted standardized mean differences for the (continuous) primary and secondary outcomes. Heterogeneity of effect measures between studies will be assessed using the chi-square test and the I-squared statistic (>50% is substantial) that are incorporated in the software.

Within each comparison, patient and intervention characteristics of the studies with the highest (25%), and lowest (25%) effect sizes will be summarized.

The sensitivity of the results will be analysed by determining how they change if the studies included in the meta-analyses are restricted to those:

- With low instead of high or uncertain risk for various potential sources of bias;

- That have had methods that facilitate the generalization of findings to patients that are regularly treated in outpatient clinics applied to them.

Analysis of subgroups or subsets

Subgroup analyses will not be specified in advance, but may be undertaken for further exploration. The results of such analyses will be cautiously interpreted.

Dissemination plans

A paper will be submitted to a leading journal in the field.

Contact details for further information

Miel Vugts

Warandelaan 2, P.O. Box 90153, Tilburg 5000 LE, The Netherlands

m.a.p.vugts@tilburguniversity.edu

Organisational affiliation of the review

Tranzo, Tilburg School of Social and Behavioral Sciences, Tilburg University

https://www.tilburguniversity.edu/research/institutes-and-research-groups/tranzo/

Review team

Mr Miel Vugts, Tranzo, Tilburg University
Dr Margot Joosen, Tranzo, Tilburg University
Dr Aglaia Zedlitz, Leiden University
Ms Jessica van der Geer, Tranzo, Tilburg University
Professor Hubertus Vrijhoef, Maastricht University Medical Center

Anticipated or actual start date
01 July 2016

Anticipated completion date
14 April 2017

Funding sources/sponsors
Tranzo, Tilburg University; Ciran Rehabilitation Centers, The Netherlands

Conflicts of interest
None known

Language
English

Country
Netherlands

Subject index terms status
Subject indexing assigned by CRD

Subject index terms
Adult; Behavior Therapy; Chronic Pain; Computers; Humans; Internet; Medically Unexplained Symptoms; Pain Management; Somatoform Disorders; Syndrome; Telemedicine; Treatment Outcome

Stage of review
Ongoing

Date of registration in PROSPERO
13 December 2016

Date of publication of this revision
13 December 2016

Stage of review at time of this submission	Started	Completed
Preliminary searches	Yes	Yes
Piloting of the study selection process	Yes	Yes
Formal screening of search results against eligibility criteria	Yes	Yes
Data extraction	Yes	No
Risk of bias (quality) assessment	Yes	Yes
Data analysis	No	No

PROSPERO
International prospective register of systematic reviews
The information in this record has been provided by the named contact for this review. CRD has accepted this information in good faith and registered the review in PROSPERO. CRD bears no responsibility or liability for the content of this registration record, any associated files or external websites.
